# Genotyping, sequencing and analysis of 140,000 adults from Mexico City

**DOI:** 10.1038/s41586-023-06595-3

**Published:** 2023-10-11

**Authors:** Andrey Ziyatdinov, Jason Torres, Jesús Alegre-Díaz, Joshua Backman, Joelle Mbatchou, Michael Turner, Sheila M. Gaynor, Tyler Joseph, Yuxin Zou, Daren Liu, Rachel Wade, Jeffrey Staples, Razvan Panea, Alex Popov, Xiaodong Bai, Suganthi Balasubramanian, Lukas Habegger, Rouel Lanche, Alex Lopez, Evan Maxwell, Marcus Jones, Humberto García-Ortiz, Raul Ramirez-Reyes, Rogelio Santacruz-Benítez, Abhishek Nag, Katherine R. Smith, Amy Damask, Nan Lin, Charles Paulding, Mark Reppell, Sebastian Zöllner, Eric Jorgenson, William Salerno, Slavé Petrovski, John Overton, Jeffrey Reid, Timothy A. Thornton, Gonçalo Abecasis, Jaime Berumen, Lorena Orozco-Orozco, Rory Collins, Regeneron Genetics Center, Gonçalo Abecasis, Adolfo Ferrando, Michael Cantor, Giovanni Coppola, Andrew Deubler, Aris Economides, Katia Karalis, Luca A. Lotta, Lyndon J. Mitnaul, John D. Overton, Jeffrey G. Reid, Alan Shuldiner, Katherine Siminovitch, Christina Beechert, Erin D. Brian, Laura M. Cremona, Hang Du, Caitlin Forsythe, Zhenhua Gu, Kristy Guevara, Michael Lattari, Alexander Lopez, Kia Manoochehri, Manasi Pradhan, Raymond Reynoso, Ricardo Schiavo, Maria Sotiropoulos Padilla, Chenggu Wang, Sarah E. Wolf, Amelia Averitt, Nilanjana Banerjee, Dadong Li, Sameer Malhotra, Justin Mower, Mudasar Sarwar, Deepika Sharma, Jeffrey C. Staples, Jay Sundaram, Sean Yu, Aaron Zhang, Mona Nafde, George Mitra, Sujit Gokhale, Andrew Bunyea, Janice Clauer, Krishna Pawan Punuru, Sanjay Sreeram, Gisu Eom, Benjamin Sultan, Vrushali Mahajan, Eliot Austin, Koteswararao Makkena, Sean O’Keeffe, Tommy Polanco, Ayesha Rasool, William J. Salerno, Lance Zhang, Boris Boutkov, Evan Edelstein, Alexander Gorovits, Ju Guan, Alicia Hawes, Olga Krasheninina, Adam J. Mansfield, Evan K. Maxwell, Suying Bao, Kathie Sun, Chuanyi Zhang, Manuel Allen Revez Ferreira, Kathy Burch, Adrian Campos, Lei Chen, Sam Choi, Liron Ganel, Sheila Gaynor, Benjamin Geraghty, Akropravo Ghosh, Salvador Romero Martinez, Christopher Gillies, Lauren Gurski, Joseph Herman, Michael Kessler, Jack Kosmicki, Adam Locke, Priyanka Nakka, Anthony Marcketta, Arden Moscati, Aditeya Pandey, Anita Pandit, Jonathan Ross, Carlo Sidore, Eli Stahl, Maria Suciu, Peter VandeHaar, Sailaja Vedantam, Scott Vrieze, Rujin Wang, Kuan-Han Wu, Bin Ye, Blair Zhang, Olivier Delaneau, Maya Ghoussaini, Jingning Zhang, Brian Hobbs, Jon Silver, William Palmer, Rita Guerreiro, Jan Freudenberg, Amit Joshi, Antoine Baldassari, Cristen Willer, Sarah Graham, Jonas Bille Nielsen, Mary Hass, Niek Verweij, George Hindy, Jonas Bovijn, Tanima De, Parsa Akbari, Luanluan Sun, Olukayode Sosina, Arthur Gilly, Peter Dornbos, Juan Rodriguez-Flores, Moeen Riaz, Manav Kapoor, Gannie Tzoneva, Momodou W. Jallow, Anna Alkelai, Ariane Ayer, Veera Rajagopal, Sahar Gelfman, Vijay Kumar, Jacqueline Otto, Neel Parikshak, Aysegul Guvenek, Jose Bras, Silvia Alvarez, Jessie Brown, Jin He, Hossein Khiabanian, Marcus B. Jones, Esteban Chen, Jaimee Hernandez, Michelle G. LeBlanc, Jason Mighty, Nirupama Nishtala, Nadia Rana, Jennifer Rico-Varela, Mexico City Prospective Study, Jonathan R. Emberson, Richard Peto, Abraham Garduño-Martinez, Abril Garcia-Lopez, Adrian Abarca-Cardoso, Adriana Caballero-Mondragon, Adriana Gutierrez-Parra, Adriana Leticia Diaz-Avila, Alan Emiliano Bautista-Hernandez, Alberto Méndez-Villalba, Aldo Shaid Ramos-Hernandez, Alejandra Alejo-Salazar, Alejandra Angelica Perez-Moncada, Alejandra Martinez, Alejandra Peralta-Gallardo, Alejandro Flores-Magana, Alfa Izamar Benitez-Garcia, Alicia González-Castillo, Alicia Villegas-Esparza, Alma Delia Morales-Bravo, Alma Fernanda Mora-Negrete, Alma Hernandez-Galicia, Alma Rosa Arenas-García, Alma Rosa Valentin-Martinez, Amalia Paredes-Rojas, Ambar Nayeli Flores-Sanchez, Amelia Ortiz-Jaen, America Juarez-Salazar, América Victoria Cervantes-Torres, Amparo Luviano-Martínez, Ana del Carmen Alejandro-Perez, Ana Dominguez-Alvarado, Ana Isabel Fuentes-Alvarado, Ana Karen Arreola-Olvera, Ana Laura Bautista-Sanchez, Ana Lilia Enríquez-Álvarez, Ana Lilia Reynoso-Valverde, Ana María Isidro-Cid, Ana Montserrat Lechuga-Mendoza, Andrea Esquivel-Mejía, Andrea Galvino-Antonio, Andrea Gomez-Luna, Andres Martinez-Martinez, Anel Aragón-Domínguez, Angelica Gamboa-Romero, Angelica Guerrero, Angelica Ruiz-Hernandez, Antonia González-María, Araceli Martínez-Santana, Araceli Rojas-Vásquez, Arcelia Rojas-Santamaría, Armida Sánchez-Corral, Athzin Berenice Rosas-Avila, Beatriz Cruz-Acevedo, Beatriz Gonzalez-Ibañes, Beatriz Rojas, Beatriz Velázquez-Mancilla, Belen Escalona-Franco, Bernardo Ochoa-Morales, Braulio Rivera-Cortés, Brenda Castañeda-Gazpar, Brenda J. Calderon-Garcia, Brenda Jimena Jimenez-Gutierrez, Brian Orlando Sanchez-Martin, Carlos Alberto Toxqui-Rico, Carlos Antonio Clemente-Montano, Carlos Daniel Jimenez-Gutierrez, Casandra Alvarez-Meneses, Catalina Gasca-Velázquez, Cecilia Luna-Barroso, César Marín-Pérez, Cinthia Calderon-Camacho, Cinthia Hernandez-Perez, Cinthia Xóchitl Hernández-Peralta, Clarinet Castillo-Rioja, Claudia Bustamante-Durán, Claudia Elizabeth Espinosa-Quintana, Claudia Lilia Galicia-Flores, Claudia Lizbeth Villagomez-Piña, Cynthya Berenice Sierra-Martinez, Daniel Fernández-Corona, Daniel Ordaz-Jiménez, Daniela Oreli Hernandez-Castillo, Daniela Ramirez-Aranda, Dante Zazhil Lopez-Guzman, Diana del Monte-Homobono, Diana Isabel Gonzalez-Enciso, Diana Laura Bolanos-Hernandez, Edith Elizabeth Valdez-Solano, Edith Gonzalez-Torres, Edson Alfonso Mercado-Hernández, Eduardo Alvarado-Valle, Elisa Morales-Martinez, Elizabet Gonzalez, Elsa Yadira Díaz-Martínez, Elvia Isabel Vázquez-Torres, Elvira Ramos-Mendoza, Emiliano del Rio-Gonzalez, Erika Alpizar-Flores, Erika García-García, Erika Pérez-Romero, Esmeralda Sanchez-Martinez, Estefania Perez-Perez, Estela Beatriz López-García, Estela Elisabeth Moran-De Los Santos, Esther Jerónimo-Hernández, Eva María Estefes-Hernández, Evelin Sanchez-Alvarez, Felipe de Jesus Ramirez-Tinajero, Felipe Rivera-Cortés, Francisca Ana Yetzy Lopez-Tellez, Francisco Barajas-Soto, Francisco Javier Garcia-Gonzalez, Francisco Javier Ruvalcaba-López, Gabriel Enrique Jimenez-Vasquez, Gabriela López-Villaseca, Gabriela Paredes-Cruz, Gabriela Rivera-Arredondo, Gardenia Nieto-Valenciano, Genaro Balderas-Martinez, Genoveva Limon, Gerardo Álvarez-Mancilla, Gerardo Fernando Gómez-Dorantes, Gladis Villegas-Ramirez, Gloria Cruz-Angeles, Gloria Hernández-Buendía, Grecia Jimenez-Perez, Guadalupe América Juárez-Salazar, Guadalupe Garduño-Loyola, Hector Hugo Villaseñor-Flores, Hector M. Velasco, Hector Valentin Villanueva-Cervantes, Hectorchavez Mendiola, Hilda Nelly Rodríguez-Neria, Hipatia Lobato-Garcia, Hortencia Torres-Morales, Idith Fabiola Hernández-Peralta, Ingrid Alejandra Ochoa-Ramos, Irais Morales-Casillas, Irene Abuhatab, Irma Garduño-Medina, Irma Palacios-Rivas, Irving Hernandez-Machuca, Irving Israel Ramirez-Ramirez, Isabel Dominguez-Ursula, Isamar Prado-Morales, Israel Adrian Barrios-Custodia, Ivan Abrajan-García, Ivonne Jazmín Aguilar-Flores, Jaime Alfonso Rodriguez-Castro, Jaime Lee Alvarado-Lopez, Jaqueline Guadarrama-Fernández, Jaqueline Lopez-Lopez, Jaredhia Nathaly Pablo-Bautista, Jedini Paola Martinez-Ramirez, Jennifer Mendoza-Mendoza, Jessica Elena Vázquez-Bustamamnte, Joaquín Edmundo Ramírez-Gonzalez, Jorge Hernández-Arellano, Jorge L. Ocana-Monroy, Jorge Ricardo Medina-Torres, Jose Alberto Zavala-Barrera, Jose Cristian Alexis Lemus-Enciso, José Juan Barajas-Gónzalez, José Juan Castañeda-Dorantes, Jose Luis Ocana-Monroy, Josefina Alvarado-Calderón, Josefina Sanchez-Escudero, Joselyn Adali Garcia-Pantoja, Juan Adan Hernandez-Salinas, Juan Carlos Cruz-Hernandez, Juan Carlos Medina-Hernández, Juan Carlos Rodríguez-Ramírez, Juan Gabriel Pérez-Álvarez, Juan Pablo Hernandez-Canales, Juan Rubén Marines-Álvarez, Juana Patricia Romero-Becerril, Julio César Gómez-Dorantes, Julio Ortiz-Sanchez, Karina Adriana Ramos-Perez, Karina Ayala-Escamilla, Karina Sánchez-Ramírez, Karla Patricia Zárate-Barrios, Laura Arroyo-Garfias, Laura Cordoba-Barrios, Laura Limon-Espinoza, Laura Magallón-Nava, Lesley Geraldine Rodriguez-Camacho, Leslie Andrea Avendano-Baltierra, Leslie Nancy Rubio-Rojas, Leticia Cruz-Castañeda, Leticia Martínez-Morales, Lezly Fernanda Arias-Lezama, Lilia Reséndiz-Galván, Liliana Rodríguez-Ayala, Liliana Solano-Vazquez, Lina Velazco-Valdez, Lizbeth Armendáriz-Zahuantitla, Lizbeth Castro, Lucía Torres-Vázquez, Luis Antonio Loa-Orellana, Luis Arturo Vazquez-Padilla, Luis Brandon Toriz-Nava, Luis Ivan Salcedo-Sandoval, Luis Manuel Valdez-Rivera, Luz Xochiquetzalit Morales-Torres, Maciel Areli Camacho-Estrella, Macrina Tapia-Gómez, Magali Abigail Caballero-Sanchez, Magaly Lizbeth Martínez-López, Magdalena Sánchez-Salinas, Marco Antonio Gonzalez-Carranza, Marco Antonio Montes-Mérida, Marco Antonio Salazar-Giron, Margarita Mirta Torres-Rodríguez, María Alejandra Meléndez-Hernández, María Alejandra Ramos-Mendoza, Maria Alexandra Dominguez-Romero, María Antonia-González, María Aurora Pérez-Vargas, María Beatriz Rojas-Aguilar, María Cristina Ruiz-Flores, Maria de los A ngeles Chavez-Corona, María del Carmen Montiel-Pérez, María del Carmen Novelo-Aguilar, María Elena Espinoza-Pérez, María Elena González-Ruiz, María Estela Maya-Colin, Maria Fernanda Kennedy-Vazquez, Maria Hernandez-Soler, María Isabel Medina-Torales, María Olvera-González, Maria Priscila Hernandez-Melendez, María Teresa Villa-Botello, Mariana Andrea Labastida-Luna, Mariana Bolanos-Orduna, Maribel Rodríguez-Ledezma, Marisol Gomez-Collado, Marisol López-Arredondo, Marissa Villa-Ayala, Martha Alvarez-Marin, Martha Decimo-Canales, Martha Flores-Hernández, Martin Flores-Ortiz, Martin Linas-Sanchez, Mauricio Marin-Sanchez, Mayeli Salado-Bazán, Mayra Chagolla-Reyes, Mayranni Marquez-Jimenez, Miguel Angel Martinez-Medina, Miguel Salgado-Martinez, Misael Olivos-Rivera, Moisés Sánchez-Cejudo, Mónica Ernestina Gónzalez-Ramos, Monica Gomez-Abad, Mónica Irineo-Ugarte, Mónica Martínez, Mónica Martínez-Márquez, Nancy Abigail Castillo-Ramos, Nancy Patricia Hernández-Galicia, Natalia Guadalupe Elizarraras-Torres, Natalia Tinoco-Hernandez, Neri Reyna-Salgado, Noé Velázquez-Mandujano, Noemí Zurita-Morán, Norma Alicia Esteban-Cruz, Norma Angelica Orbe-Sierra, Norma Patricia Solís-Calvillo, Oliverio Rivera-Cortez, Omar Santiago-Perez, Oswaldo Hernandez-Camacho, Oswaldo Israel Gómez-Dorantes, Patricia Andrés-Gutiérrez, Patricia Cuarenta-Medina, Patricia Rez, Patricio Marquez-Espino, Paula Morales-Godinez, Paulina Monserrat Montano-Rojas, Ramses Alejandro Bravo-Juarez, Reyna Aurora Garza-Zepeda, Reyna Margarita Contreras-Hernández, Ricardo Manuel Ruiz-Zepeda, Ricardo Marquez-Nunez, Roberto Fabian Pelaez-Granados, Roberto Solera-Calvo, Rocío Hernández-López, Rosalinda García-Anaya, Rosario Dafne Lujan-Velazquez, Rosario Pérez Rul-Rivero, Rosaura Vazquez-Reyes, Rubén Espinoza-Peña, Ruperto García-Pérez, Salomón González-Garrido, Samantha Nayeli-De la Rosa Rodríguez, Sandra Lizbet Colon-Serrano, Sanjuana García-Hernández, Santiago Olvera-Arriaga, Santos Pérez-Gallardo, Sara Heras-Santiago, Sara Yazmin Flores-Jimenez, Sarahi Montiel-Sanchez, Sérgio César Bruno-Baltazar, Sheila Cruz-Martinez, Sibyl Nadir Luna-Ramírez, Silvia Ávila-Jaen, Silvia Cervantes-Saldívar, Socrates Cardenas-Valencia, Sonia Angélica Saldívar-Sánchez, Tania Michelle Sanchez-Damiz, Tomás Dorantes-Rosas, Vera Lopez-Sanchez, Verónica Colín-Hernández, Veronica Perez-Elizalde, Veronica Sanchez-Ortega, Verónica Santos-Sánchez, Veronica Velasco-Nava, Vianey Hernandez-Piña, Violeta Flores-Ramírez, Viridiana Ruiz-Gonzalez, Xiadani Paulina Mejia-Villegas, Xóchitl Cano-Goméz, Yacquelín Mondragón-Martínez, Yamili Evaristo-Montes, Yaquelinne Carcia-Muñoz, Yaxum Mendoza-Rocafuerte, Yazmin Parra-Ortega, Yeni Guadalupe Guadarrama-Fernández, Yojahira Martinez-Morales, Zaira Rebeca Martinez-Vite, Zoraida Lucio-Olmedo, Fernando Rivas-Reyes, Raúl Ramírez-Reyes, Adrián Garcilazo-Ávila, Carlos Gonzáles-Carballo, Diego Aguilar-Ramírez, Doreen Zhu, Eirini Trichia, Erwin Chiquete, Fiona Bragg, Gary Whitlock, Louisa Gnatiuc Friedrichs, Natalie Staplin, Omar Yaxmehen Bello-Chavolla, Richard Haynes, Robert Clarke, Sarah Lewington, William Herrington, Alejandra Vergara, Elizabeth Barrera-Sánchez, Georgina Del Vecchyo-Tenorio, Margarita González-Ruiz, Paulina Baca-Peynado, Tianshu Liu, Yunhe Wang, Adriana Lucrecia Wong y. Wong, Clementina Magos, Fredrik Romer, Herendira Zambrano Martínez, James Wheeler, Kathleen Emmens, Linda Youngman, Martin Radley, Martha Solano Sanchez, Matthew Lacey, Michael R. Hill, Nigel Plunkett, Paul Taylor, Richard Shellard, Sarah Clark, Tim Williams, Gustavo Olaiz Fernandez, Lisa Holland, Malaquias López Cervantes, Aris Baras, Michael R. Hill, Jonathan R. Emberson, Jonathan Marchini, Pablo Kuri-Morales, Roberto Tapia-Conyer

**Affiliations:** 1 Regeneron Genetics Center, Tarrytown, NY USA; 2 Clinical Trial Service Unit and Epidemiological Studies Unit, Nuffield Department of Population Health, University of Oxford, Oxford, UK; 3 MRC Population Health Research Unit, Nuffield Department of Population Health, University of Oxford, Oxford, UK; 4 Experimental Research Unit from the Faculty of Medicine (UIME), National Autonomous University of Mexico (UNAM), Mexico City, Mexico; 5 Oxford Kidney Unit, Churchill Hospital, Oxford, UK; 6 Instituto Nacional de Medicina Genómica, Tlalpan, Mexico City, Mexico; 7 Centre for Genomics Research, Discovery Sciences, Research and Development Biopharmaceuticals, AstraZeneca, Cambridge, UK; 8 AbbVie, North Chicago, IL USA; 9 Department of Biostatistics, University of Michigan, Ann Arbor, MI USA; 10 Instituto Tecnológico y de Estudios Superiores de Monterrey, Monterrey, Mexico; 11 Faculty of Medicine, National Autonomous University of Mexico, Mexico City, Mexico; 12 Instituto Nacional de Ciencias Médicas y de la Nutrición, Salvador Zubirán Hospital, Mexico City, Mexico; 13 Research Division, Instituto Nacional de Geriatría, Mexico City, Mexico

**Keywords:** Genetic variation, DNA sequencing, Evolutionary genetics, Genetic association study

## Abstract

The Mexico City Prospective Study is a prospective cohort of more than 150,000 adults recruited two decades ago from the urban districts of Coyoacán and Iztapalapa in Mexico City^1^. Here we generated genotype and exome-sequencing data for all individuals and whole-genome sequencing data for 9,950 selected individuals. We describe high levels of relatedness and substantial heterogeneity in ancestry composition across individuals. Most sequenced individuals had admixed Indigenous American, European and African ancestry, with extensive admixture from Indigenous populations in central, southern and southeastern Mexico. Indigenous Mexican segments of the genome had lower levels of coding variation but an excess of homozygous loss-of-function variants compared with segments of African and European origin. We estimated ancestry-specific allele frequencies at 142 million genomic variants, with an effective sample size of 91,856 for Indigenous Mexican ancestry at exome variants, all available through a public browser. Using whole-genome sequencing, we developed an imputation reference panel that outperforms existing panels at common variants in individuals with high proportions of central, southern and southeastern Indigenous Mexican ancestry. Our work illustrates the value of genetic studies in diverse populations and provides foundational imputation and allele frequency resources for future genetic studies in Mexico and in the United States, where the Hispanic/Latino population is predominantly of Mexican descent.

## Abstract (web summary)

Genotype and exome sequencing of 150,000 participants and whole-genome sequencing of 9,950 selected individuals recruited into the Mexico City Prospective Study constitute a valuable, publicly available resource of non-European sequencing data.

## Main

Latin American populations harbour extensive genetic diversity that reflects a complex history of migration throughout the Americas, post-Colonial admixture between continents and more recent population growth^2,3^. The distinct patterns of genomic variation that exist in these populations have led to key insights into the genetic architecture of rare and common diseases. Founder populations are prevalent throughout Latin America, and analyses of deleterious variants that segregate at higher frequency in these populations have identified clinically relevant new variants^4,5^. Moreover, Latin American populations include a significant proportion of Indigenous American subpopulations that have mostly remained genetically uncharacterized. Admixture among European, Indigenous American and African ancestry populations can result in allele frequency distributions that substantially diverge from ancestral populations. Variants that are rare in one ancestry population but common in another may therefore segregate at a higher frequency in an admixed population. This leads to opportunities for new discoveries in these populations that may be missed when studying single ancestry populations^6^. For example, in a study of Mexican adults^7^, a haplotype in the *SLC16A11* locus that is common in Indigenous Americans but rare in Europeans was strongly associated with type 2 diabetes. In addition to increasing opportunities for variant discovery, genetic analyses of admixed populations can result in improvements in fine-mapping owing to differences in patterns of linkage disequilibrium (LD)^8^.

Unfortunately, despite the numerous opportunities afforded from studying Latin American populations, Hispanic/Latino individuals from such populations constitute less than 1% of all individuals in genetic population research despite forming nearly 10% of the global population. By contrast, European populations constitute more than 80% of participants in genomic databases but account for less than 20% of people worldwide^9^. Recent initiatives that target specific populations^10,11^ or involve large biobanks (such as the Million Veterans Program (https://www.research.va.gov/mvp) and TOPMed (https://imputation.biodatacatalyst.nhlbi.nih.gov)) have increased the number of Hispanic/Latino individuals included in genetic research, but a sizable gap remains. Additional large genetic studies of Latin American populations are therefore needed to help bridge this gap and enable the implementation of precision medicine for these populations.

Between 1998 and 2004, 159,755 participants aged at least 35 years from two contiguous urban districts of Mexico City (63,833 from Coyoacán and 95,922 from Iztapalapa) were recruited into the Mexico City Prospective Study (MCPS)^1^. Here we describe genome-wide array genotyping and whole-exome sequencing (WES) on the entire MCPS cohort, and high-coverage whole-genome sequencing (WGS) on a subset of 9,950 participants. We provide a comprehensive genetic profile of the MCPS cohort that reveals patterns of relatedness, identical-by-descent (IBD) sharing and runs of homozygosity (ROH). By incorporating genotypes from 716 Indigenous individuals from 60 out of the 68 recognized ethnic populations in Mexico, we apply a range of scalable techniques to finely characterize population structure, continental admixture and local ancestry in the MCPS cohort.

We also provide a survey of variants according to annotation and frequency, with a particular emphasis on genes that exhibit homozygous loss-of-function variation. Moreover, we estimate ancestry-specific allele frequencies from America, Africa and Europe at 142 million variants, a 10-fold increase over existing resources, made available through a public browser (https://rgc-mcps.regeneron.com/). Last, we use the phased WGS dataset as a reference panel to impute genotypes into the full cohort and examine the quality of this imputed dataset compared with the exome sequencing dataset and a TOPMed-imputed version of the cohort.

## Overview of genetic datasets

Of the 159,755 MCPS participants, a blood sample was successfully taken, processed and stored for 155,453 (97.3%). Of these samples, DNA was successfully extracted for 146,068 (94.0%) participants and sent for genotyping and exome sequencing. After initial quality control (QC) procedures (Methods), genotyping array data were available for 138,511 participants and exome data were available for 141,046 (Supplementary Table 1 provides key baseline characteristics of the 141,046 participants with exome data). Of the exomes sequenced, 98.7% of the samples had 90% of the targeted bases covered at 20× or higher. After applying machine-learning methods to filter out low-quality variants, we identified a total of 9.3 million variants, including 4.0 million variants across the coding regions of 19,110 genes. In total, 98.7% of the coding variants were rare (minor allele frequency (MAF) < 1%) (Table 1, Supplementary Table 2 and Methods), and 1.4 million were specific to the MCPS cohort when compared with variants discovered by the UK Biobank exome sequencing study^8^, TOPMed^11^ and gnomAD^12^ (Supplementary Table 3). Among the coding variants identified were 1,233,054 (median of 14,900 alleles per individual) synonymous, 2,526,776 (13,585 alleles per individual) missense and 233,650 (354 alleles per individual) putative loss-of-function (pLOF) variants (Table 1). The proportion of singletons (30.9%) was much lower than observed in other datasets (for example, 46.8% in the UK Biobank exome study^8^) owing to the way in which households of participants in close neighbourhoods were recruited, which resulted in extensive familial relatedness (as described in the next section). As expected, the proportion of singletons increased to 36.5% when we restricted the analysis to individuals related less than first degree. The proportion was further increased to 39.2% when we restricted the analysis to individuals related less than third degree. In addition, we observed more homozygous pLOF variants in the MCPS cohort compared with a sample-size-matched version of the UK Biobank exome dataset (Supplementary Table 4).

**Table 1:** Number of coding variants discovered in exome sequencing of 141,046 MCPS participants

Variant category (all transcripts)	*N* variants (% with MAC = 1)	Median number of alternative alleles per participant (IQR)	Mean number of alternative alleles per participant (s.d.)	Median number of variants per participant (IQR)	Mean number of variants per participant (s.d.)
Coding regions	4,037,949 (30.87)	29,119 (291)	29,126 (235)	20,849 (628)	20,795 (454)
Predicted function					
In-frame indels	44,469 (30.97)	281 (16)	281 (12)	207 (14)	207 (10)
Synonymous	1,233,054 (28.04)	14,900 (169)	14,902 (134)	10,641 (320)	10,615 (234)
Missense	2,526,776 (31.4)	13,585 (163)	13,588 (127)	9,722 (300)	9,699 (217)
Likely benign	535,622 (27.94)	9,908 (121)	9,910 (93)	6,748 (191)	6,735 (138)
Possibly deleterious	1,441,180 (31.17)	3,564 (74)	3,564 (56)	2,857 (113)	2,853 (82)
Likely deleterious	549,974 (35.38)	114 (16)	114 (12)	111 (15)	112 (12)
pLOF	233,650 (40.06)	354 (20)	354 (15)	273 (19)	273 (14)
Start lost	9,768 (36.1)	27 (5)	27 (4)	21 (4)	21 (3)
Stop gain	77,589 (39.05)	85 (9)	85 (7)	67 (8)	67 (6)
Stop lost	3,539 (35.21)	13 (3)	13 (3)	10 (2)	10 (2)
Splice donor	26,364 (40.06)	38 (6)	38 (5)	30 (5)	30 (4)
Frameshift	96,098 (41.29)	146 (14)	147 (10)	113 (13)	114 (9)
Splice acceptor	20,292 (40.81)	44 (6)	44 (5)	32 (5)	32 (4)

Variants were annotated using Ensembl variant effect predictor. The predicted function for each variant was defined as the most deleterious consequence spanning all protein-coding transcripts in Ensembl (v.100). Indels, insertions and deletions; IQR, interquartile range; MAC, minor allele count.

A subset of 9,950 individuals from the MCPS also underwent WGS, with a mean sequencing depth of 38.5×. After filtering, we identified 131.9 million variants in total, of which 1.5 million were coding variants (Supplementary Tables 5 and 6 and Methods). Moreover, 96.2% of the variants were rare variants (MAF < 1%). There were 31.5 million distinct WGS variants compared with variants discovered in the TOPMed^11^ and gnomAD^12^ WGS datasets (Supplementary Table 7).

We compared the WGS and WES data in the overlapping set of 9,950 individuals to examine the amount of coding variation called. The WGS dataset led to a 2.3% absolute increase in the amount of coding variation when using the canonical gene transcript to annotate variants in a matched set of 9,950 samples **(**Extended Data Table 1). In detail, 93.2%, 4.5% and 2.3% of the union set of sites were called in both datasets, in the WGS-only dataset and in the WES-only dataset, respectively (Supplementary Table 8). When variants were annotated by the most deleterious consequence across all transcripts of a gene, the WGS dataset had 4.6% more coding variants (Supplementary Table 9). In detail, 91.1%, 6.6% and 2.3% of the union set of sites were called in both datasets, the WGS-only dataset and the WES-only dataset, respectively (Supplementary Table 10). When restricted to exome sequencing capture regions only, the differences between WGS and WES were much smaller (Supplementary Tables 11–14). Supplementary Tables 15–18 compare WGS and WES results for variants with an alternative allele frequency value of <1%. The number of variants that were specific to the WGS and WES datasets and overlapped with TOPMed were similar to the number of variants that overlapped with gnomAD (Supplementary Tables 19–22). Concordance of genotype calls between the WGS and WES datasets in 9,950 overlapping samples was high, with a mean SNP discordance of 0.0085% (Supplementary Table 23).

A total of 138,511 individuals from the MCPS were genotyped using an Illumina Global Screening Array v.2 beadchip and passed QC control (Methods and Supplementary Table 24). Array genotypes were highly concordant with WGS and WES genotypes in overlapping samples (mean biallelic SNP discordance of 0.03–0.04% for both datasets) (Supplementary Table 23).

## Relatedness

The genotyping array dataset enabled us to investigate familial relatedness within the cohort, which was expected to be high owing to the household recruitment strategy used (Methods). We used shared IBD segments to infer relatedness to avoid estimation biases in samples from admixed populations that can occur when using methods based on population allele frequency estimates. We applied KING software to unphased data and the hap-IBD and IBDkin methods to a phased array dataset (Methods). Both unphased and phased approaches produced comparable relatedness results (Supplementary Fig. 1).

**Fig. 1:**
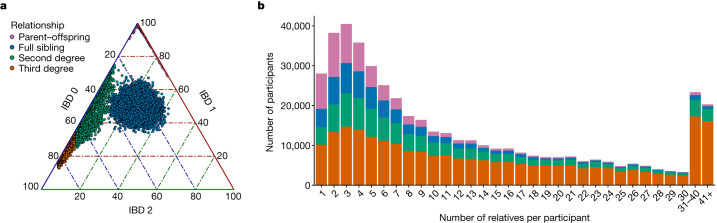
Familial relatedness. **a**, Percentage of the genome estimated to have zero, one or two alleles identical-by-descent (IBD). **b**, Distribution of the number of relatives that participants have in the MCPS cohort. The height of each bar shows the count of participants with the stated number of relatives. The colours indicate the proportions of each relatedness class within each bar.

**Extended Data Fig. 1:**
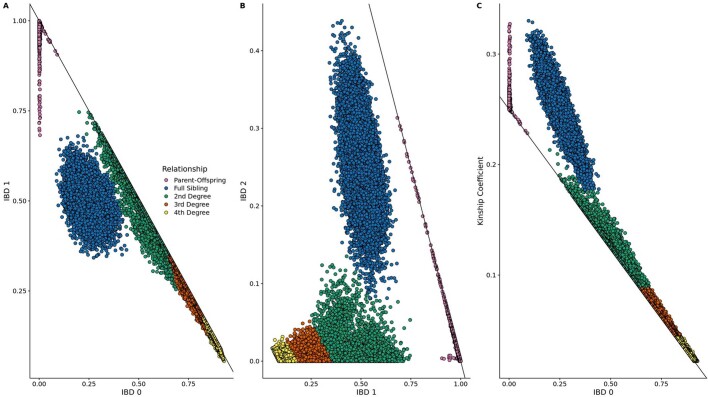
Pairwise measures of relatedness. (A) IBD0 vs IBD1, (B) IBD1 vs IBD2, and (C) IBD0 vs Kinship coefficient.

**Extended Data Fig. 2:**
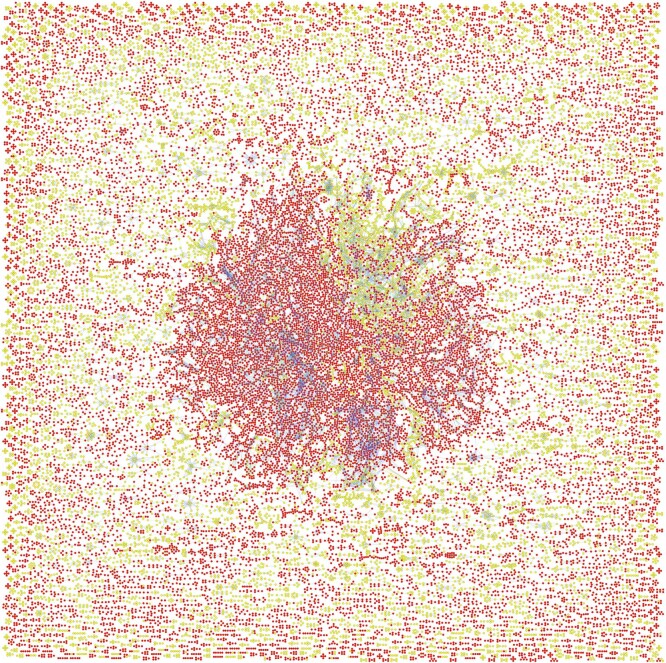
Graph of second-degree family networks of size four or greater. Plot created using the Graphviz software with the sfdp layout engine which uses a “spring” model that relies on a force-directed approach to minimize edge length.

Figure 1a and Extended Data Fig. 1 illustrate the extensive relatedness identified in the MCPS cohort. There are 31,597 parent–offspring, 29,482 full sibling, 47,080 second-degree relative and 120,180 third-degree relative pairs. Nearly 71% (97,953 individuals) in the MCPS have at least one relative in the study who is third-degree or closer, and many of the MCPS participants have multiple close relatives (Fig. 1b). The largest connected component in a graph of individuals with third-degree relationships or closer involved 22% of the cohort (30,682 individuals) (Supplementary Fig. 2). These levels of relatedness are much higher than those observed in the UK Biobank but are comparable to the Geisinger Health Study^13^ (both the MCPS and the Geisinger Health Study recruited participants from regions with families living in close proximity) (Supplementary Table 25). We used PRIMUS to reconstruct 22,766 first-degree family networks containing a total of 65,777 individuals with a median size of 2.9, up to a maximum size of 48 people, including 3,595 nuclear families (Supplementary Fig. 3 and Supplementary Table 26). A graph of 14,428 individuals with second-degree family networks of a size greater than four highlights the complexity of the patterns of relatedness and partial clustering of relationships within districts of Coyoacán and Iztapalapa (Extended Data Fig. 2). The largest connected component in this graph contained 9,180 individuals. We also investigated relationships within and across the two districts (Supplementary Table 27). With reconstruction of pedigree networks in the MCPS, we were able to investigate the proportion of relatives who cross boundaries and have residences in different districts. Among the first-degree relatives, only 3% of parent–child pairs and 7% of full sibling pairs lived in different districts. The percentages of second-degree and third-degree relative pairs with residences in different districts was 13% and 17%, respectively, which is much lower than would be expected if there was random mixing of individuals from the contiguous districts. Notably, although there was a marked 10–15% decrease in the percentages of second-degree or third-degree relative pairs who both had a residence in the Coyoacán district compared with first-degree relationship types, the percentages of relative pairs who had a residence in the Iztapalapa district remained relatively consistent across relationship types (Supplementary Table 27). These results provide insight into patterns of migration (or lack thereof) within families between the Coyoacán and Iztapalapa districts.

## Population structure

**Fig. 2:**
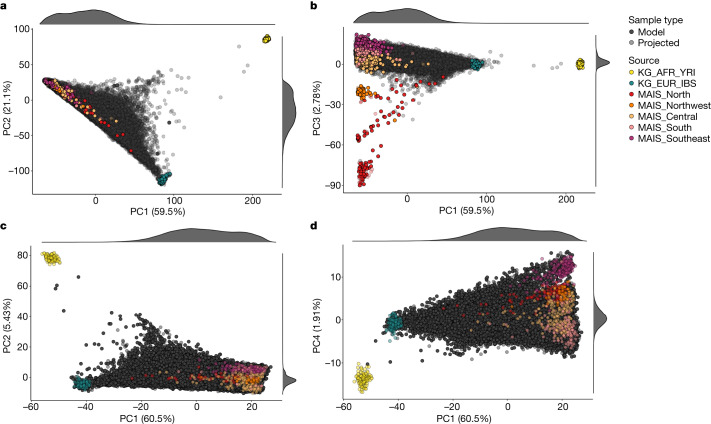
PCA analysis of the MCPS data together with Indigenous Mexican, European and African datasets. **a**,**b**, A total of 500 MCPS samples were used for analyses, together with 108 African Yoruba (KG_AFR_YRI) and 107 European Iberian (KG_EUR_IBS) samples from the 1000 Genomes project (KG) dataset, and 591 unrelated samples from 60 Indigenous Mexican populations corresponding to central, southern, southeastern, northern and northwestern regions of Mexico from the MAIS. **c**,**d**, These analyses used an unrelated set of 58,051 samples together with the 1000 Genomes and MAIS samples. All other MCPS samples are projected onto the axes.

We used a variety of complementary analysis approaches to characterize the ancestry composition and heterogeneity of MCPS individuals relative to the pre-Columbian population structure in Mexico. First, we applied principal component analysis (PCA) to a reference dataset of 108 African (Yoruba) and 107 European (Iberian) samples from the 1000 Genomes dataset. We also analysed 591 unrelated samples from 60 Indigenous Mexican populations corresponding to central, southern, southeastern, northern and northwestern regions of Mexico from the Metabolic Analysis of an Indigenous Sample (MAIS) dataset^3^ (Methods, Fig. 2 and Supplementary Fig. 4). We included a representative set of unrelated MCPS samples (*n* = 500) in the PCA model-fitting procedure and projected the remaining 138,011 MCPS samples onto the inferred principal component (PC) axes. Figure 2a shows that PC1 and PC2 separate Indigenous Mexican, African and European samples, and that MCPS samples lie on the axis between Indigenous Mexican and European samples. Figure 2b shows that PC3 differentiates Indigenous Mexican geographical subpopulations and suggests that the majority of MCPS samples have ancestry from central, southern and southeastern Mexico.

To provide more focus on the genetic variation within the MCPS dataset, we applied PCA to a filtered array dataset of 58,051 unrelated MCPS samples, with all other MCPS samples and 1000 Genomes, Human Genome Diversity Panel (HGDP) and MAIS samples projected onto the inferred PC axes (Fig. 2c,d and Supplementary Fig. 5). This analysis further highlighted that ancestry from Indigenous groups in central, southern and southeastern Mexico was largely represented within the cohort. These regions correspond to Mesoamerica, a geographical and cultural area of rich biodiversity that was inhabited by sedentary agricultural societies during the pre-Hispanic era^3^. By contrast, ancestry from Indigenous populations in the northern and more arid regions of the country was sparsely represented in the MCPS cohort.

**Extended Data Fig. 3:**
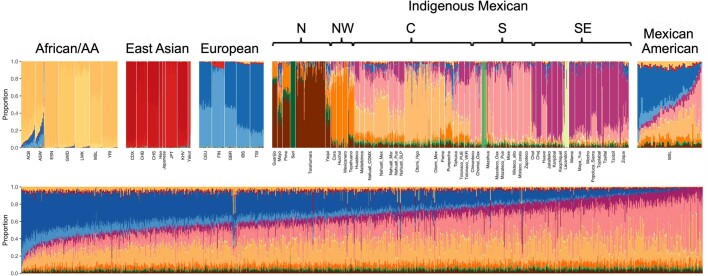
ADMIXTURE ancestry proportion estimates. The program ADMIXTURE was used to estimate per-individual ancestry proportions and population-specific allele frequencies in a panel of 3,964 reference samples, including 1,000 MCPS samples. The remaining set of 137,511 MCPS samples were projected into the admixture model using parameter estimates from the reference sample. Results are shown for the K = 18 model that attained the lowest cross-validation error. Ancestry proportion estimates for reference samples of African, European, and Indigenous American ancestry from the 1KG, HGDP, and MAIS datasets are shown in the top row and estimates for MCPS participants are shown in the bottom row. AA=African American.

We identified that stringent LD filtering was needed to avoid localized genomic regions that had increased PCA SNP loadings owing to long-range LD consistent with recent admixture (Supplementary Figs. 6–8). Parametric admixture estimation also corroborated significant ancestry proportions from Mesoamerican ancestry populations among MCPS participants (Extended Data Fig. 3 and Methods).

We applied two different haplotype-based approaches that can utilize LD between SNPs and have been shown to uncover much finer scale population structure^14,15^. The first approach used identical-by-descent (IBD) segments^16^, and the second approach measured the extent of haplotype sharing using a scalable implementation of a haplotype-copying hidden Markov model^17^ (Methods). Both of these approaches produced low-dimensional representations with a notably more ‘star-like’ structure than PCA (Supplementary Figs. 9 and 10). Combined with the ancestry proportions from the local ancestry inference (LAI; see the next section), this result highlights the ability of these approaches to more clearly delineate the contributions of Mesoamerican and European ancestry.

## Local ancestry estimation

**Fig. 3:**
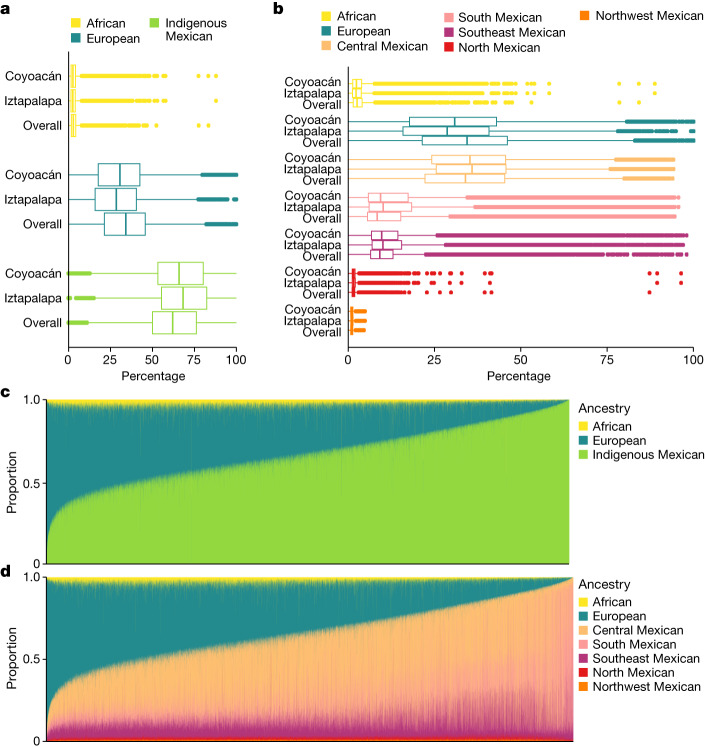
Global ancestry proportions estimated from LAI. **a**,**b**, Distributions of LAI-based global ancestry proportions for *n* = 138,511 MCPS individuals from a 7-way analysis (**b**) and reduced to 3 continental populations (**a**). **c**,**d**, Stacked bar plots of three-way (**c**) and seven-way (**d**) global ancestry proportions for *n* = 138,511 MCPS individuals.

We carried out a supervised population structure analysis by applying LAI with RFMix using a reference panel of haplotypes from Africa, Europe and America (Methods). Supplementary Fig. 11 shows local ancestry at segments genome-wide for 12 representative MCPS individuals estimated from the LAI results. Figure 3 shows population distributions of LAI-based ancestry proportion estimates, including Indigenous American ancestry from five geographical regions within Mexico. Overall, we estimated that 66.0% of autosomal ancestry was attributable to Indigenous Mexican populations, with the majority coming from central Mexico (35.6%). Southern Mexico and southeastern Mexico accounted for 15.9% and 11.8%, respectively, with much smaller amounts of ancestry attributable to northern Mexico (1.6%) and northwestern Mexico (1.1%). In addition, 2.9% and 31.1% of ancestry were attributable to African and European populations, respectively. We observed that MCPS individuals with the most Indigenous Mexican ancestry seemed to have a greater relative contribution from Indigenous populations from southern Mexico (that is, from the states of Oaxaca and Veracruz) (Supplementary Fig. 12). Moreover, lower amounts of Indigenous Mexican ancestry and higher amounts of European ancestry were observed in Coyoacán than in Iztapalapa, a result consistent with the sociodemographic characteristics of these districts.

Using 3,595 parent couples inferred from the genetic relatedness analysis, we observed significant correlation in ancestry between partner pairs (Supplementary Fig. 13), as has been observed in other studies in admixed populations^18^. Education and district explained between 0.5 and 5% of the variation in ancestry, whereas spousal ancestry explained between 15 and 26% of the variation in ancestry. This result suggests that genomic ancestry is a better predictor of the ancestry of partners than these sociodemographic factors.

**Extended Data Fig. 4:**
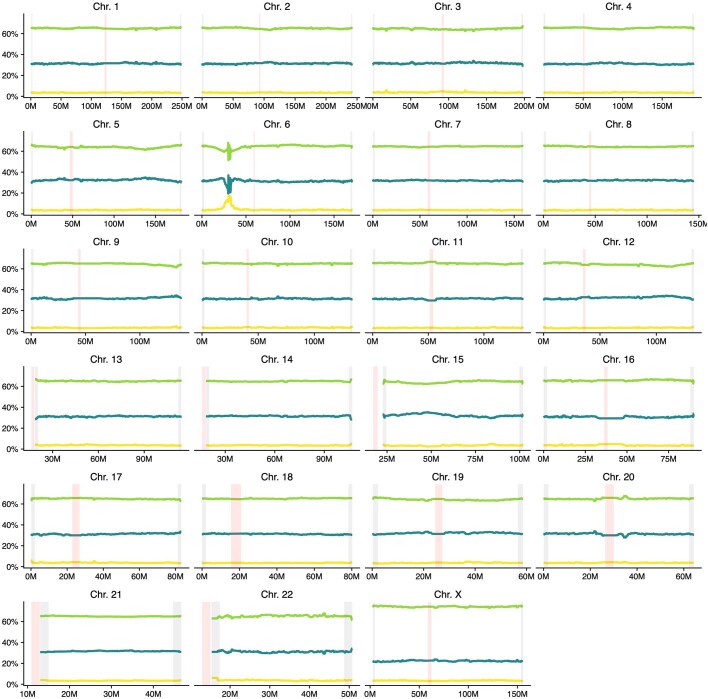
Genome-wide distribution of local ancestry proportions. The ancestry dosages inferred by RFMix are averaged across 78,833 unrelated MCPS samples and plotted along the genome. For each panel (or Chromosome) two gray rectangles denote terminal 2Mbp-length regions (of analyzed sites) at the beginning and end of Chromosome, while the red rectangle denotes the centromere region.

Extended Data Fig. 4 shows the proportion of ancestry across each chromosome from a three-way LAI analysis, and Supplementary Fig. 14 shows per-ancestry tests for departures from genome-wide ancestry proportions (Methods). This result highlighted an excess of African ancestry in and around the MHC locus on chromosome 6 (African 17.3%, *P* = 2.9 × 10^–14^; Supplementary Fig. 15), a result consistent with previous observations^19^. An additional signal on chromosome 15 showed increased European ancestry of 35.2% at position 48.38 Mb (*P* = 3.8 × 10^–8^) and spanned a region between 45.09 Mb and 52.31 Mb in and around *SLC24A5*, a gene with known function in human skin pigmentation. Variant rs1426654 in *SLC24A5* explains roughly one-third of the variation in pigmentation between Europeans and West Africans, probably being under selection in Europeans^20^.

We also observed ancestry proportions on chromosome X that exhibited increased levels of Indigenous Mexican ancestry compared with the autosomes (African 3.2%, Indigenous Mexican 73.8%, European 22.7%), a finding consistent with an imbalance of male and female contributions to admixture. Using a simplified population mixture event model^21,22^ that best fit the observed chromosome X ancestry proportions, we estimated that the proportion of Indigenous Mexican ancestry explained by female contribution was 71.3%. By contrast, for Europeans, the female contribution accounted for 7.5% (Supplementary Table 28).

## Homozygosity

**Extended Data Fig. 5:**
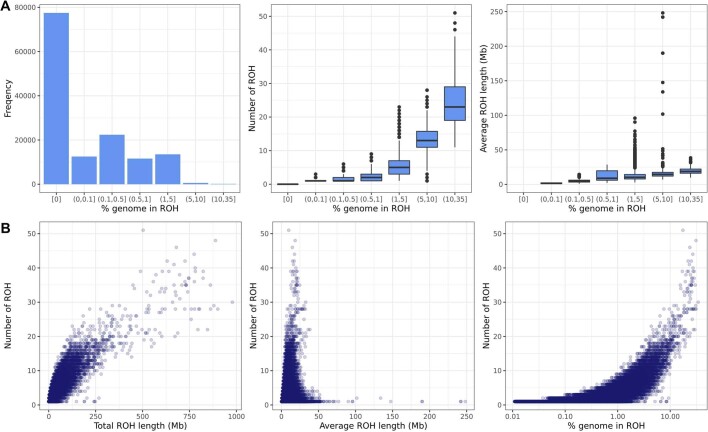
Distribution of ROH. **(a)** Histogram of the sample counts, distribution of the per-sample number of ROH segments, and distribution of per-sample average ROH segment length are given by fraction of genome in ROH for n = 138,200 samples. Data in box plots are presented with the median as the center, the box bounded by the 25^th^ and 75^th^ percentiles, whiskers extending from the box to values within 1.5*IQR (Interquartile Range), and outlying values such as minima/maxima as bpoints. **(b)** For each individual, the total length, average length, and fraction of genome in ROH is given by number of ROH.

Increased levels of homozygosity were indicated by both the relatedness analysis, which highlighted parent–offspring pairs with increased levels of sharing two alleles IBD genome-wide (Fig. 1a), and the exome variant survey, which highlighted high counts of homozygous pLOF variants compared with the UK Biobank exome dataset (Supplementary Table 4). We assessed homozygosity by estimating ROH from the phased array dataset using hap-IBD (Methods), which produced a mean homozygosity of 0.34% for all MCPS individuals. There were 60,722 MCPS participants (43.9%) who had at least one ROH segment 4 centiMorgan (cM) or longer, for which the mean homozygosity was 0.78% (Supplementary Table 29 and Extended Data Fig. 5). By comparison, for the UK Biobank data, the mean homozygosity was 0.07%, and 0.59% among the 55,206 (11.3%) participants who had at least one ROH segment ≥4 cM.

We observed that the total length and number of ROH segments were positively correlated with the proportion of ancestry native to Mexico (Supplementary Fig. 16). Overall, 79.0% of ROH segments could be assigned to Indigenous Mexican ancestry when overlaid with inferred local ancestry (Methods), which exceeded the 66.3% average amount of Indigenous Mexican ancestry in the sample. Conversely, we observed a depleted proportion of European and African ancestry in ROH segments (19.10% and 1.9%, respectively) compared with the average amount in the sample (30.2% and 3.5%, respectively), which was consistent with previous findings^23^.

The mean number of rare homozygous pLOFs (rhLOF; allele frequency of <0.1%) and the proportion of rhLOFs in ROH correlated with the proportion of the genome in ROH segments (Supplementary Fig. 17). We identified 3,763 rhLOF genotypes at 2,646 variants in 2,169 different protein-coding genes in 3,519 individuals, and 52.2% of these were found within ROH segments. Consistent with the rate of rhLOF variants and assignment of ROH segments to Indigenous American ancestry (Supplementary Table 4), segments of Indigenous Mexican ancestry accounted for 62.6% of rhLOFs, a result indicative of an ancestry-specific trend (Supplementary Table 30).

## An MCPS imputation reference panel

We created a phased haplotype imputation reference panel (MCPS10k) from the 9,950 WGS individuals utilizing sequencing reads, pedigrees and a phased array haplotype scaffold (Methods). Using the WGS trios, we estimated that haplotypes were phased with a switch error rate of 0.0024 (Methods and Supplementary Fig. 18) and observed that the switch error rate depended on ancestry proportion (Supplementary Fig. 19).

We assessed the utility of the MCPS10k reference panel for genotype imputation by imputing chromosome 2 using the phased array dataset of 67,079 MCPS individuals not included in the reference panel and pruned for relationships up to the first degree. For comparison, we also imputed the MCPS dataset using the diverse TOPMed reference panel that includes 47,159 European, 24,267 African and 17,085 admixed American genomes (Methods).

**Extended Data Fig. 6:**
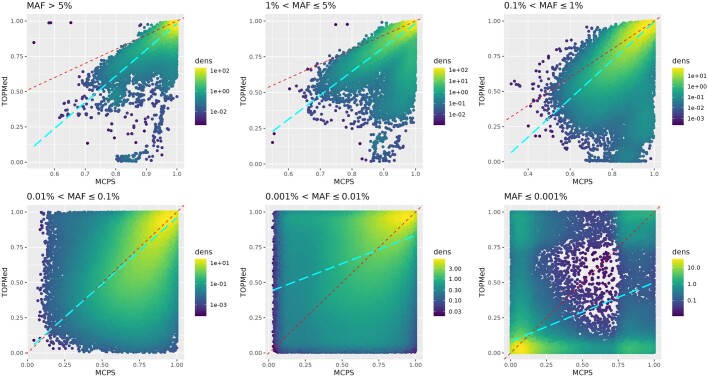
Comparison of MCPS10k and TOPMed imputation. Plots show imputation info scores from MCS10k and TOPMed imputed variants in 67,079 MCPS samples at 6,473,872 variants on chromosome 2. Each plot uses a different MAF bin. The red line is Y=X. The blue dashed line is the regression line.

MCPS10k and TOPMed imputation produced a set of 9,801,290 and 9,437,266 variants, respectively, on chromosome 2, with an imputation information score of >0.3. However, the information scores (a well-calibrated measure of accuracy) for an overlapping set of 6,473,872 variants were generally higher using MCPS10k than TOPMed for MAF bins greater than 0.01% (Extended Data Fig. 6).

**Fig. 4:**
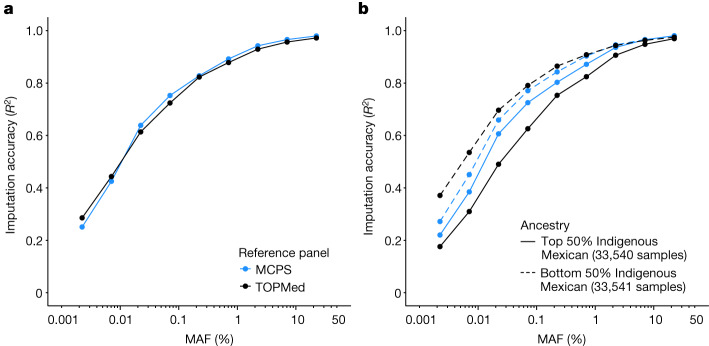
Imputation accuracy using the MCPS10k and TOPMed imputation panels. **a**,**b**, Accuracy was measured using the *R*^2^ between the imputed variants and 128,728 variants measured using exome sequencing on chromosome 2 in 67,079 MCPS samples not in (or related to) the MCPS reference panel samples. Results are stratified by allele frequency (*x* axis on log_10_ scale), reference panel and into two populations (top and bottom 50% of Indigenous Mexican ancestry shown by solid and dashed lines). **a**, Results for all samples. **b**, Results stratified by the amount of Indigenous Mexican estimated in each sample.

We compared the MCPS10k and TOPMed imputed genotypes to the exome-sequencing data at 128,728 sites on chromosome 2. Figure 4 shows the results of the imputation accuracy stratified by allele frequency, reference panel and degree of Indigenous Mexican ancestry (defined as two groupings with individuals split above and below the median proportion of Indigenous Mexican ancestry). The results showed that MCPS10k had a comparable performance with TOPMed across the entire frequency range. However, the MCPS10k panel provided the greatest imputation benefits for the samples with high proportions of Indigenous Mexican ancestry.

Finally, we assessed the imputation performance in Mexican Americans from Los Angeles (MXL) in 1000 Genomes and found that TOPMed provided improved imputation performance compared with MCPS10k (Supplementary Figs. 20 and 21). This result is probably driven by MXL samples having substantially higher European ancestry and less ancestry from central, southern and southeast Mexico than in the MCPS cohort (Supplementary Fig. 22). Similarly, the TOPMed panel provided the best performance in individuals with Peruvian ancestry from Lima (PEL), Colombian ancestry from Medellin (CLM) and Puerto Rican ancestry from Puerto Rico (PUR) from the 1000 Genomes study compared with MCPS10k (Supplementary Figs. 20 and 21). These results emphasize the value of closely matching the ancestry of imputation reference panels to the samples being studied. Although our panel provided improved imputation for individuals of Mesoamerican Mexican ancestry, additional panels may be required to provide similar benefits for other Latin American populations with admixture from different Indigenous American ancestral populations.

## Polygenic risk score transferability

**Extended Data Fig. 7:**
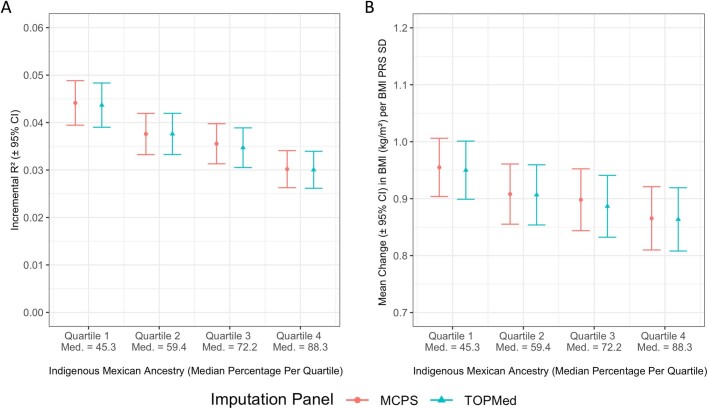
Portability of a UK Biobank (n=443,145) derived BMI PRS to MCPS (N = 119,864) individuals across imputation reference panels. In panel A, the UK Biobank PRS accuracy is assessed using the incremental R^2^ between the BMI PRS and measured BMI (kg/m^2^) in MCPS individuals, divided into quartiles (N = 29,966 per quartile) by proportion of Indigenous Mexican Ancestry. Results are also stratified by the reference panel used to impute genotype dosages in MCPS (red = MCPS, blue = TOPMed). The R^2^ measures are denoted by a circle (MCPS) or triangle (TOPMed), with vertical bars denoting the 95% confidence interval. The BMI values used in PRS derivation were transformed (RINT by sex, ancestry PCs) and adjusted for age and age^2^. Panel B displays the change in BMI per BMI PRS standard deviation (SD), with mean change represented by a circle (MCPS) or triangle (TOPMed) and vertical bars denoting the 95% confidence interval. BMI regression models were adjusted for sex, age, age^2^, and ancestry PCs. The median proportion of Indigenous Mexican ancestry in each quartile is also shown in both panel A and B.

**Extended Data Fig. 8:**
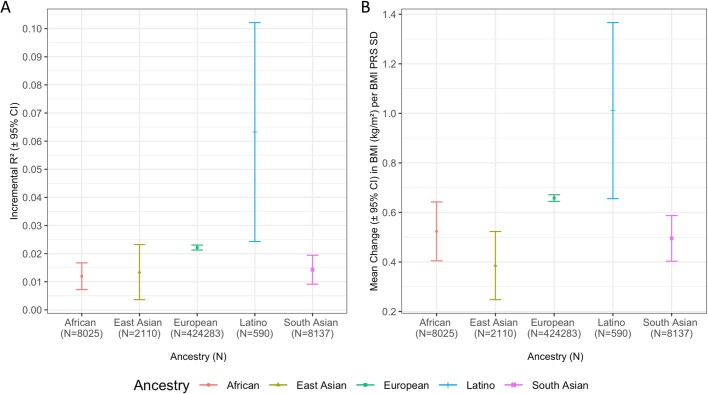
Portability of an MCPS (N=119,864) derived BMI PRS to UKB (N = 443,145) individuals. In panel A, the MCPS PRS accuracy is measured using the incremental R^2^ between the BMI PRS and measured BMI (kg/m^2^) in UK Biobank individuals, stratified by 1000 Genomes-based continental ancestry (red = African [N = 8025], lime green = East Asian [N = 2110], green = European [N = 424,283], blue = Latino [N = 590], purple = South Asian [N = 8137]). The R^2^ measures are denoted by a circle (African), triangle (East Asian), rectangle (European), dash (Latino), and dotted rectangle (South Asian), with vertical bars denoting the 95% confidence interval. The BMI values used in PRS derivation were transformed (RINT by sex, ancestry PCs) and adjusted for age and age^2^. Panel B displays the change in BMI per BMI PRS standard deviation (SD) using the same color scheme based on 1000 Genomes-based continental ancestry, with the shapes denoting the mean BMI change (circle [African], triangle [East Asian], rectangle [European], dash [Latino], and dotted rectangle [South Asian] and vertical bars denoting the 95% confidence interval. BMI Regression models were adjusted for sex, age, age^2^, and ancestry PCs.

The polygenic risk score (PRS) uses genome-wide association study (GWAS) results for a disease or trait in a given population to build an individual-level predictive score, but may not perform as well when applied to individuals from a different population^9,24^. We evaluated the performance of a body mass index (BMI) PRS derived using the UK Biobank dataset applied to MCPS participants. Extended Data Fig. 7 shows that PRS performance (measured using incremental *R*^2^) ranged from 0.044 to 0.033 in individuals in the lowest quartile to the highest quartile of Indigenous Mexican ancestry, respectively. In agreement with previous publications^25^, there was a linear relationship between ancestry and PRS accuracy (Extended Data Fig. 7a), and the gradient for the change in BMI per PRS standard deviation (Extended Data Fig. 7b) was similar to the incremental *R*^2^ findings. Of note, incremental *R*^2^ estimates for PRS based on the MCPS reference panel and TOPMed reference panels were highly similar, which aligns with the imputation findings shown in Fig. 4. Imputation accuracy in the MCPS and TOPMed panels were similar for variants with alternative allele frequency values of ≥1%, which is the threshold typically applied to summary statistics included in PRS analyses. We also quantified the performance of a BMI PRS derived using the MCPS dataset applied to UK Biobank individuals within five broad ancestral groups. Extended Data Fig. 8 demonstrates the power of the MCPS cohort to improve PRS accuracy in individuals of Latino ancestry. Although the number of individuals of Latino ancestry in the UK Biobank is small (*n* = 590), the incremental *R*^2^ (95% confidence interval) was 0.063 (0.024–0.102).

## Ancestry-specific allele frequencies

**Fig. 5:**
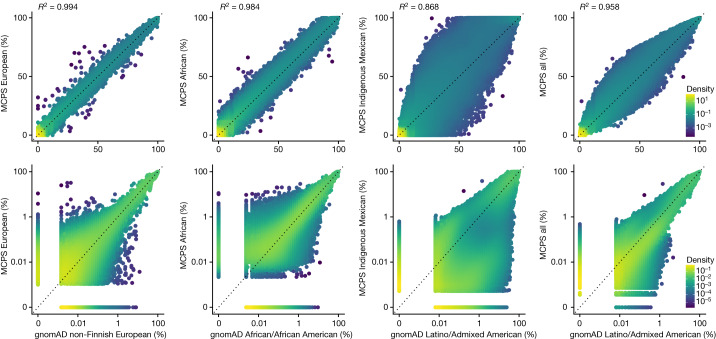
Allele frequency comparison between MCPS WES and gnomAD. Allele frequencies on linear (top) and log (bottom) scale. The comparisons from left to right are MCPS European versus gnomAD non-Finnish European, MCPS African versus gnomAD African/African American, MCPS Indigenous Mexican versus gnomAD Latino/Admixed American, and MCPS all versus gnomAD Latino/Admixed American.

**Extended Data Fig. 9:**
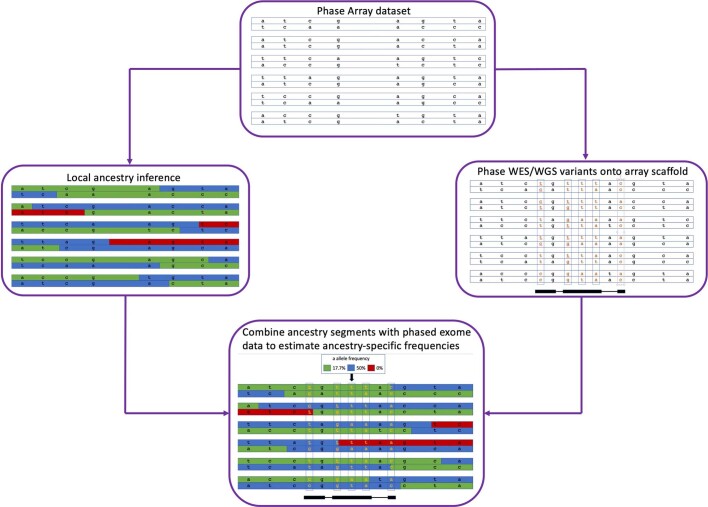
Schematic of ancestry-specific allele frequency estimation. The estimation proceeds in 4 stages. To start with the array dataset is phased to produce a scaffold of common variants (top). Then local ancestry inference (LAI) is applied to the phased array dataset (left). In parallel, the WES and WGS variants are phased onto the phased array scaffold (right). Then finally the phased exome variant dataset is overlayed onto the local ancestry estimates to assign ancestry to every allele in the WES and WGS datasets (bottom). The process is probabilistic and interpolates the ancestry probabilities at the WES and WGS sites from the flanking array sites.

**Extended Data Fig. 10:**
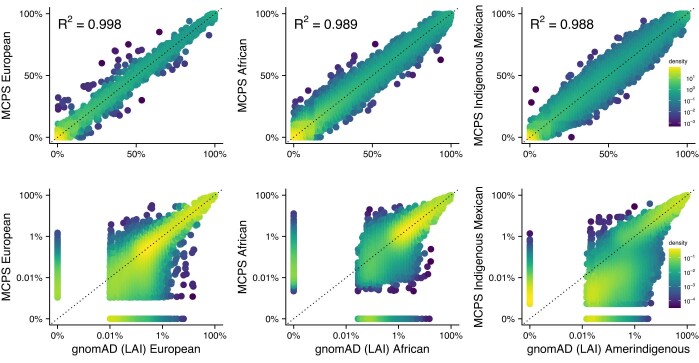
Allele frequency comparison between MCPS WES and gnomAD LAI estimates. Allele frequencies on linear (top) and log (bottom) scale. The comparisons from left to right are MCPS European vs gnomAD (LAI) European, MCPS African vs gnomAD (LAI) African, MCPS Indigenous Mexican vs gnomAD Amerindigenous. The gnomAD (LAI) refers to an extension to the gnomAD v3 database with local ancestry resolved allele frequency estimates for Latino/Admixed American samples in gnomAD (see URLs). The number of high-quality variants overlapped between MCPS WES and gnomAD (LAI) is 241,307, 211,105 and 201,624 for European, African and Amerindigenous ancestries, respectively.

We combined the LAI results with the phased WES and WGS datasets to estimate Indigenous Mexican, African and European allele frequencies at 141,802,412 genetic variants, increasing by tenfold the number of LAI-resolved frequencies currently available in the gnomAD browser (see Methods and schematic in Extended Data Fig. 9). These frequencies are available in a public browser (https://rgc-mcps.regeneron.com/). The median effective sample sizes across sites (Methods) for estimation of Indigenous Mexican, African and European ancestry were 91,856, 4,312 and 42,009, respectively, for WES variants, and 6,549, 341 and 3,058, respectively, for WGS variants. For comparison, the median sample sizes in gnomAD v.3.1 are 7,639, 20,719 and 34,014 for Latino/Admixed American, African and non-Finnish European ancestries, respectively. Figure 5 compares WES allele frequency estimates using our deconvolution approach in the MCPS dataset to the more direct approach used in gnomAD v.3.1. European allele frequencies showed substantial agreement (*r*^2^ = 0.994), whereas African allele frequencies only showed slightly less agreement (*r*^2^ = 0.987), despite greater heterogeneity in African ancestry populations and the lower median African sample size in the MCPS cohort. Supplementary Fig. 23 compares MCPS WGS and gnomAD allele frequencies. Extended Data Fig. 10 and Supplementary Fig. 24 show high concordance between MCPS WES and WGS frequencies and gnomAD LAI-resolved frequencies (https://gnomad.broadinstitute.org).

The estimated frequencies used all MCPS samples, as restricting to unrelated individuals resulted in an 8.6% reduction in the number of polymorphic variants. We developed a new method to compute relatedness-corrected allele frequencies using identical-by-descent (IBD) segments (Methods and Supplementary Fig. 25) and found little difference between the relatedness-corrected frequencies and those estimated using all individuals, and allele frequencies of variants observed in an unrelated subset (Supplementary Figs. 26 and 27). We provide all three versions of allele frequencies in variant call files (VCFs) that are downloadable from the MCPS browser.

Extended Data Table 2 shows the allele frequencies at 46 loci previously reported to show trait associations in contemporary Mexican or other Latin American populations. For example, the top SNP associated with type 2 diabetes at the *SLC16A11* locus^7^ (rs75493593) had an overall frequency of 36% but ancestry-specific allele frequencies of 0.1%, 0.7% and 53% in African, European and Indigenous Mexican populations, respectively. This result is in agreement with previous estimates reported by the SIGMA Type 2 Diabetes Consortium^7^. Another notable example occurs at the *IGF2* locus, where the pLOF splice acceptor variant rs149483638 that confers protection against type 2 diabetes^26^ had an overall frequency of 23% but ancestry-specific allele frequencies of 0.06%, 0.05% and 35% in African, European and Indigenous Mexican populations, respectively. Moreover, the rare *MC4R* missense variant rs79783591 associated with obesity^27^ is absent from the gnomAD browser but had an overall frequency of 1.1% in the MCPS, with an inferred Indigenous Mexican frequency of 1.6%, and African and European frequencies of less than 0.05%.

We used the three-way LAI segments to further decompose the annotated variants into three continental populations. Across all variant classes, the highest levels of variation were found in African segments and lower levels in Indigenous Mexican and European segments, a result consistent with the demographic history of these populations (Supplementary Table 31). For example, the estimated mean number of pLOF variants in Indigenous Mexican, European and African genomes were 347, 361 and 427, respectively, although rare homozygous pLOF variants were more frequent among longer ROHs of Indigenous American ancestry (as shown above).

## Discussion

The MCPS genetic data resources described in this study represent one of the largest in Mexico so far. The data also represent one of the most extensive sequencing studies in individuals of non-European ancestry and a major contribution towards the goal of increasing the diversity of genetic collections. Through scalable genotype and haplotype-based approaches to characterize fine-scale population structure and admixture, we traced the Indigenous American component of ancestry within MCPS individuals to predominantly Mesoamerican Indigenous populations from central, southern and southeastern Mexico. Many Indigenous populations within southern Mexico belong to the Oto-mangue linguistic family (for example, Mixteco, Zapoteco and Ixcateco), whereas most Indigenous populations from southeastern Mexico belong to the Maya linguistic family (Maya, Chuj, Ixil and Awakateco). Genetic analyses in Mexico have previously shown that Indigenous populations in these regions share extensive genetic similarly that closely aligns with linguistic family membership^3,28^. Meanwhile, Indigenous populations in the central region of Mexico (for example, Otomi and Nahuatl) show pronounced genetic similarity (that is, low measures of pairwise *F*_st_) despite spanning distinct linguistic families (for example, Oto-mangue, Yuto-nahua and Totonaco-tepehua). By contrast, ancestry from Aridoamerican Indigenous populations in the northern most regions of the country and from Mesoamerican populations in the northwest state of Nayarit (Cora, Tepehuano, Mexicanero and Huichol) was underrepresented in the MCPS dataset. Moreover, there was evidence of sex imbalance on the X chromosome^28^. The higher proportion of Mesoamerican ancestry on chromosome X is consistent with sex-biased gene flow resulting from predominantly male European colonization of the Americas^29^ and may have implications for health disparities between men and women in light of the longer ROH, and rarer pLOF variants, that tracked with Mesoamerican ancestry. Such health disparities may also be compounded by the assortative mating observed in the MCPS, which has been well-documented elsewhere^30^. Furthermore, IBD-based analyses revealed extensive and complex patterns of relatedness among participants within Coyoacán and Iztapalapa, which largely reflected the household-based recruitment strategy of the study. Together, our analyses have characterized the MCPS cohort as one of largest genetic studies with both high levels of admixture and relatedness compared with other large genetic datasets such as the UK Biobank.

We developed a new approach for estimating ancestry-specific allele frequencies that leverages local ancestry information and interpolated ancestry at called variants in the MCPS WES and WGS datasets. This increased (by tenfold) both the number of variants with ancestry-specific allele frequencies and the Indigenous Mexican effective sample size used for estimating allele frequencies from WES data. Without a suitable reference dataset of ancestry-specific allele frequencies, efforts to diagnose and interpret genomic variants in the context of rare disorders are encumbered as it is difficult to distinguish previously unreported or undersampled ancestry-specific variants from potentially pathogenic variants. Our study expands the availability of such allelic information, which is made accessible to the genomics research community through the MCPS Variant browser to facilitate future discoveries.

The MCPS WES and WGS datasets substantially add to the global survey of characterized genomic variants by more than 31 million variants. Additionally, we uncovered increased levels of homozygosity and homozygous pLOF variants attributable to Indigenous Mexican ancestry, which indicates a role for future studies of admixed Mexicans as a previously untapped resource for the study of homozygous loss-of-function alleles in humans. Comparing WGS and WES datasets in the same set of 9,950 samples, we found that the WGS dataset led to a 2.3% absolute increase in the amount of coding variation when using the canonical gene transcript to annotate variants. Further quantitative comparisons in larger datasets, such as the UK Biobank, will be needed to examine the overall utility of WGS over WES and imputation for new causal variant discovery.

From our investigations, we found that the imputation accuracy with MCPS10k was comparable to the TOPMed reference panel across the entire frequency range. Moreover, MCPS10K provided the highest imputation accuracy for individuals with high proportions of Mesoamerican ancestry. In theory, a combination of the MCPS10k and TOPMed reference panels should result in improved imputation performance than using either reference panel alone. There are, however, significant challenges in bringing together large WGS datasets across studies for imputation, which motivates the need for new approaches that can combine imputation results from different panels. The results from our study highlight the need for large diverse WGS datasets from many different populations and the potential for a single worldwide reference panel to increase representation and parity in imputation accuracy across ancestries.

With the increasing availability of large-scale genetic data from biobanks and cohort studies, PRS values are becoming more widely used for predicting genetic risk of diseases and quantitative traits in clinical settings^31^. PRS values, however, have largely been constructed using European-ancestry GWAS results, and recent studies^32^ have shown that Eurocentric bias in PRS can result in reduced performance in non-European ancestry populations. In this study, we evaluated the performance and portability of PRS across ancestries using individuals from the MCPS and UK Biobank. We found that PRS values for BMI constructed using European ancestry individuals from the UK Biobank resulted in prediction accuracy that increased linearly with proportional European ancestry in the MCPS, in which the lowest utility of PRS was among MCPS participants with high Indigenous American ancestry. The prediction accuracy of a MCPS-derived PRS was also highest in the Latino ancestry group in the UK Biobank among five 1000-Genomes-based continental ancestry groups. These results reaffirm the importance of constructing PRS values using samples with ancestry that closely match the target population. Increasing the genetic ancestry diversity of participants in future genetic studies will be essential to advancing the utility of PRS across global populations, and we demonstrate the potential for the MCPS dataset to be a valuable resource for advancing polygenic prediction in admixed Latino populations.

The publicly available MCPS genetic resources, particularly the allele frequency and imputation databases, will contribute to future studies and serve as a major asset for understanding the genetic basis of diseases across Mexico and in the United States, where there is a large population of individuals of Mexican descent. In addition, our study can serve as a blueprint for obtaining new insight into the complex genetic architecture of other diverse populations. The utility of the MCPS genetic resource has recently been demonstrated through its contribution to the discovery of loss-of-function variation in *GPR75* and *INHBE* that are protective against obesity^27^ and type 2 diabetes^33^ respectively, and in the replication of the *MAP3K15* association with lower glycosylated haemoglobin and diabetes^34^. Moreover, the analysis of MCPS exomes was instrumental in estimating that *MC4R* heterozygous deficiency is more than seven times greater in Mexico than in the United Kingdom^27^. Future studies will link genetic variation to other disease traits through cross-cohort meta-analyses, increase the resolution of fine-mapping, further explore the construction and portability of PRS in the Mexican population, leverage admixture, relatedness and household information to potentially boost the power of discovery in association studies and utilize Mendelian randomization to uncover causal relationships between modifiable exposures and disease.

## Methods

### Recruitment of study participants

The MCPS was established in the late 1990s following discussions between Mexican scientists at the National Autonomous University of Mexico (UNAM) and British scientists at the University of Oxford about how best to measure the changing health effects of tobacco in Mexico. These discussions evolved into a plan to establish a prospective cohort study that could investigate not only the health effects of tobacco but also those of many other factors (including factors measurable in the blood)^1^. Between 1998 and 2004, more than 100,000 women and 50,000 men 35 years of age or older (mean age 50 years) agreed to take part, were asked questions, had physical measurements taken, gave a blood sample and agreed to be tracked for cause-specific mortality. More women than men were recruited because the study visits were predominantly made during working hours when women were more likely to be at home (although visits were extended into the early evenings and at weekends to increase the proportion of men in the study).

Participants were recruited from randomly selected areas within two contiguous city districts (Coyoacán and Iztapalapa). These two districts have existed since the pre-Hispanic period and are geographically close to the ancient Aztec city of Tenochtitlan. Originally, Indigenous populations settled there, but over the centuries, the population dynamics have substantially changed. Many people from Spain, including the conqueror Hernán Cortés, resided in Coyoacán while the capital of New Spain was being built over the ruins of Tenochtitlan. The modern populations of Coyoacán and Iztapalapa derive largely from the development of urban settlements and migrations from the 1950s to the 1970s. Over this period, both districts, but particularly Iztapalapa, received large numbers of Indigenous migrants from the central (Nahuas, Otomies and Purepechas), south (Mixtecos, Zapotecos and Mazatecos) and southeast (Chinantecos, Totonacas and Mayas) regions of the country.

### Blood sample collection, processing and storage, and DNA extraction

At recruitment, a 10-ml venous EDTA blood sample was obtained from each participant and transferred to a central laboratory using a transport box chilled (4–10 °C) with ice packs. Samples were refrigerated overnight at 4 °C and then centrifuged (2,100*g* at 4 °C for 15 min) and separated the next morning. Plasma and buffy-coat samples were stored locally at −80 °C, then transported on dry ice to Oxford (United Kingdom) for long-term storage over liquid nitrogen. DNA was extracted from buffy coat at the UK Biocentre using Perkin Elmer Chemagic 360 systems and suspended in TE buffer. UV-VIS spectroscopy using Trinean DropSense96 was used to determine yield and quality, and samples were normalized to provide 2 μg DNA at 20 ng μl^–1^ concentration (2% of samples provided a minimum 1.5 µg DNA at 10 ng µl^–1^ concentration) with a 260:280 nm ratio of >1.8 and a 260:230 nm ratio of 2.0–2.2.

### Exome sample preparation and sequencing and QC

Genomic DNA samples were transferred to the Regeneron Genetics Center from the UK Biocentre and stored in an automated sample biobank at –80 °C before sample preparation. DNA libraries were created by enzymatically shearing DNA to a mean fragment size of 200 bp, and a common Y-shaped adapter was ligated to all DNA libraries. Unique, asymmetric 10 bp barcodes were added to the DNA fragment during library amplification to facilitate multiplexed exome capture and sequencing. Equal amounts of sample were pooled before overnight exome capture, with a slightly modified version of IDT’s xGenv1 probe library; all samples were captured on the same lot of oligonucleotides. The captured DNA was PCR amplified and quantified by quantitative PCR. The multiplexed samples were pooled and then sequenced using 75 bp paired-end reads with two 10 bp index reads on an Illumina NovaSeq 6000 platform on S4 flow cells. A total of 146,068 samples were made available for processing. We were unable to process 2,628 samples, most of which failed QC during processing owing to low or no DNA being present. A total of 143,440 samples were sequenced. The average 20× coverage was 96.5%, and 98.7% of the samples were above 90%.

Of the 143,440 samples sequenced, 2,394 (1.7%) did not pass one or more of our QC metrics and were subsequently excluded. Criteria for exclusion were as follows: disagreement between genetically determined and reported sex (*n* = 1,032); high rates of heterozygosity or contamination (VBID > 5%) (*n* = 249); low sequence coverage (less than 80% of targeted bases achieving 20× coverage) (*n* = 29); genetically identified sample duplicates (*n* = 1,062 total samples); WES variants discordant with the genotyping chip (*n* = 8); uncertain linkage back to a study participant (*n* = 259); and instrument issue at DNA extraction (*n* = 6). The remaining 141,046 samples were then used to compile a project-level VCF (PVCF) for downstream analysis using the GLnexus joint genotyping tool. This final dataset contained 9,950,580 variants.

### Whole genome sample preparation and sequencing and QC

Approximately 250 ng of total DNA was enzymatically sheared to a mean fragment size of 350 bp. Following ligation of a Y-shaped adapter, unique, asymmetric 10 bp barcodes were added to the DNA fragments with three cycles of PCR. Libraries were quantified by quantitative PCR, pooled and then sequenced using 150 bp paired-end reads with two 10 bp index reads on an Illumina NovaSeq 6000 platform on S4 flow cells. A total of 10,008 samples were sequenced. This included 200 mother–father–child trios and 3 more extended pedigrees. The rest of the samples were chosen to be unrelated to third degree or closer and enriched for parents of nuclear families. The average mean coverage was 38.5× and 99% of samples had mean coverages of >30×, and all samples were above 27×.

Of the 10,008 samples that were whole-genome sequenced, 58 (0.6%) did not pass one or more of our QC metrics and were subsequently excluded. Reasons for exclusion were as follows: disagreement between genetically determined and reported sex (*n* = 16); high rates of heterozygosity or contamination (VBID > 5%) (*n* = 10); genetically identified sample duplicates (*n* = 19 total samples); and uncertain linkage back to a study participant (*n* = 14). The remaining 9,950 samples were then used to compile a PVCF for downstream analysis using the GLnexus joint genotyping tool. This final dataset contained 158,464,363 variants.

### Variant calling

The MCPS WES and WGS data were reference-aligned using the OQFE protocol^35^, which uses BWA MEM to map all reads to the GRCh38 reference in an alt-aware manner, marks read duplicates and adds additional per-read tags. The OQFE protocol retains all reads and original quality scores such that the original FASTQ is completely recoverable from the resulting CRAM file. Single-sample variants were called using DeepVariant (v.0.10.0) with default WGS parameters or custom exome parameters^35^, generating a gVCF for each input OQFE CRAM file. These gVCFs were aggregated and joint-genotyped using GLnexus (v.1.3.1). All constituent steps of this protocol were executed using open-source software.

### Identification of low-quality variants from sequencing using machine learning

Similar to other recent large-scale sequencing efforts, we implemented a supervised machine-learning algorithm to discriminate between probable low-quality and high-quality variants^8,12^. In brief, we defined a set of positive control and negative control variants based on the following criteria: (1) concordance in genotype calls between array and exome-sequencing data; (2) transmitted singletons; (3) an external set of likely ‘high quality’ sites; and (4) an external set of likely ‘low quality’ sites. To define the external high-quality set, we first generated the intersection of variants that passed QC in both TOPMed Freeze 8 and gnomAD v.3.1 genomes. This set was additionally restricted to 1000 genomes phase 1 high-confidence SNPs from the 1000 Genomes project^36^ and gold-standard insertions and deletions from the 1000 Genomes project and a previous study^37^, both available through the GATK resource bundle (https://gatk.broadinstitute.org/hc/en-us/articles/360035890811-Resource-bundle). To define the external low-quality set, we intersected gnomAD v3.1 fail variants with TOPMed Freeze 8 Mendelian or duplicate discordant variants. Before model training, the control set of variants were binned by allele frequency and then randomly sampled such that an equal number of variants were retained in the positive and negative labels across each frequency bin. A support vector machine using a radial basis function kernel was then trained on up to 33 available site quality metrics, including, for example, the median value for allele balance in heterozygote calls and whether a variant was split from a multi-allelic site. We split the data into training (80%) and test (20%) sets. We performed a grid search with fivefold cross-validation on the training set to identify the hyperparameters that returned the highest accuracy during cross-validation, which were then applied to the test set to confirm accuracy. This approach identified a total of 616,027 WES and 22,784,296 WGS variants as low-quality (of which 161,707 and 104,452 were coding variants, respectively). We further applied a set of hard filters to exclude monomorphs, unresolved duplicates, variants with >10% missingness, ≥3 mendel errors (WGS only) or failed Hardy–Weinberg equilibrium (HWE) with excess heterozgosity (HWE *P* < 1 × 10^–30^ and observed heterozygote count of >1.5× expected heterozygote count), which resulted in a dataset of 9,325,897 WES and 131,851,586 WGS variants (of which 4,037,949 and 1,460,499 were coding variants, respectively).

### Variant annotation

Variants were annotated as previously described^38^. In brief, variants were annotated using Ensembl variant effect predictor, with the most severe consequence for each variant chosen across all protein-coding transcripts. In addition, we derived canonical transcript annotations based on a combination of MANE, APPRIS and Ensembl canonical tags. MANE annotation was given the highest priority followed by APPRIS. When neither MANE nor APPRIS annotation tags were available for a gene, the canonical transcript definition of Ensembl was used. Gene regions were defined using Ensembl release 100. Variants annotated as stop gained, start lost, splice donor, splice acceptor, stop lost or frameshift, for which the allele of interest was not the ancestral allele, were considered predicted loss-of-function variants. Five annotation resources were utilized to assign deleteriousness to missense variants: SIFT; PolyPhen2 HDIV and PolyPhen2 HVAR; LRT; and MutationTaster. Missense variants were considered ‘likely deleterious’ if predicted deleterious by all five algorithms, ‘possibly deleterious’ if predicted deleterious by at least one algorithm and ‘likely benign’ if not predicted deleterious by any algorithm.

### Genotyping

Samples were genotyped using an Illumina Global Screening Array (GSA) v.2 beadchip according to the manufacturer’s recommendations. A total of 146,068 samples were made available for processing, of which 145,266 (99.5%) were successfully processed. The average genotype call rate per sample was 98.4%, and 98.4% of samples had a call rate above 90%. Of the 145,266 samples that were genotyped, 4,435 (3.1%) did not pass one or more of our QC metrics and were subsequently excluded. Reasons for exclusion were as follows: disagreement between genetically determined and reported sex (*n* = 1,827); low-quality samples (call rates below 90%) (*n* = 2,276); genotyping chip variants discordant with exome data (*n* = 44); genetically identified sample duplicates (*n* = 1,063 total samples); uncertain linkage back to a study participant (*n* = 268); and sample affected by an instrument issue at DNA extraction (*n* = 6). The remaining 140,831 samples were then used to compile a PVCF for downstream analysis. This dataset contained 650,380 polymorphic variants.

### Genotyping QC

The input array data from the RGC Sequencing Laboratory consisted of 140,831 samples and 650,380 variants and were passed through the following QC steps: checks for consistency of genotypes in sex chromosomes (steps 1–4); sample-level and variant-level missingness filters (steps 5 and 6); the HWE exact test applied to a set of 81,747 third-degree unrelated samples, which were identified from the initial relatedness analysis using Plink and Primus (step 7); setting genotypes with Mendelian errors in nuclear families to missing (step 8); and a second round of steps 5–7 (step 9). Plink commands associated with each step are displayed in column 2 (Supplementary Table 9). The final post-QC array data consisted of 138,511 samples and 559,923 variants.

### Array phasing

We used Shapeit (v.4.1.3; https://odelaneau.github.io/shapeit4) to phase the array dataset of 138,511 samples and 539,315 autosomal variants that passed the array QC procedure. To improve the phasing quality, we leveraged the inferred family information by building a partial haplotype scaffold on unphased genotypes at 1,266 trios from 3,475 inferred nuclear families identified (randomly selecting one offspring per family when there was more than one). We then ran Shapeit one chromosome at a time, passing the scaffold information with the --scaffold option.

### Exome and whole genome phasing

We separately phased the support-vector-machine-filtered WES and WGS datasets onto the array scaffold. The phased WGS data constitute the MCPS10k reference panel. For the WGS phasing, we used WhatsHap (https://github.com/whatshap/whatshap) to extract phase information in the sequence reads and from the subset of available trios and pedigrees, and this information was fed into Shapeit (v.4.2.2; https://odelaneau.github.io/shapeit4) through the --use-PS 0.0001 option. Phasing was carried out in chunks of 10,000 and 100,000 variants (WES and WGS, respectively) and using 500 SNPs from the array data as a buffer at the beginning and end of each chunk. The use of the phased scaffold of array variants meant that chunks of phased sequencing data could be concatenated together to produce whole chromosome files that preserved the chromosome-wide phasing of array variants. A consequence of this process is that when a variant appeared in both the array and sequencing datasets, the data from the array dataset were used.

To assess the performance of the WGS phasing process, we repeated the phasing of chromosome 2 by removing the children of the 200 mother–father–child trios. We then compared the phase of the trio parents to that in the phased dataset that included the children. We observed a mean switch error rate of 0.0024. Without using WhatsHap to leverage phase information in sequencing reads, the mean switch error rate increased to 0.0040 (Supplementary Fig. 23).

### Relatedness, pedigree reconstruction and network visualization

The relatedness-inference criteria and relationship assignments were based on kinship coefficients and probability of zero IBD sharing from the KING software (https://www.kingrelatedness.com). We reconstructed all first-degree family networks using PRIMUS (v.1.9.0; https://primus.gs.washington.edu/primusweb) applied to the IBD-based KING estimates of relatedness along with the genetically derived sex and reported age of each individual. In total, 99.3% of the first-degree family networks were unambiguously reconstructed. To visualize the relationship structure in the MCPS, we used the software Graphviz (https://graphviz.org) to construct networks such as those presented in Supplementary Fig. 5. We used the sfdp layout engine which uses a ‘spring’ model that relies on a force-directed approach to minimize edge length.

### Measuring IBD segments and homozygosity

To identify IBD segments and to measure ROH, we ran hap-ibd (v.1.0; https://github.com/browning-lab/hap-ibd) using the phased array dataset of 138,511 samples and 538,614 sites from autosomal loci. Hap-ibd was run with the parameter min-seed=4, which looks for IBD segments that are at least 4 cM long. We filtered out IBD segments in regions of the genome with fourfold more or fourfold less than the median coverage along each chromosome following the procedure in IBDkin (https://github.com/YingZhou001/IBDkin), and filtered out segments overlapping regions with fourfold less than the median SNP marker density (Supplementary Fig. 28). For the homozygosity analysis, we intersected the sample with the exome data to evaluate loss-of-function variants, which resulted in a sample of 138,200. We further overlaid the ROH segments with local ancestry estimates, and assigned ancestry where the ancestries were concordant between haplotypes and posterior probability was >0.9, assigning ancestry to 99.8% of the ROH.

### PCA

We used the workflow implemented in the R package bigsnpr (https://privefl.github.io/bigsnpr). In brief, pairwise kinship coefficients were estimated using Plink (v.2.0) and samples were pruned for first-degree and second-degree relatedness (kinship coefficient < 0.0884) to obtain a set of unrelated individuals. LD clumping was performed with a default LD *r*^*2*^ threshold of 0.2, and regions with long-range LD were iteratively detected and removed using a procedure based on evaluating robust Mahalanobis distances of PC loadings. Sample outliers were detected using a procedure based on *K-*nearest neighbours. PC scores and loadings for the first 20 PCs were efficiently estimated using truncated singular value decomposition (SVD) of the scaled genotype matrix. After removal of variant and sample outliers, a final iteration of truncated SVD was performed to obtain the PCA model. The PC scores and loadings from this model were then used to project withheld samples, including related individuals, into the PC space defined by the model using the online augmentation, decomposition and procustes algorithm. For each PC analysis in this study, variants with MAF < 0.01 were removed.

### Admixture analysis

Admixture (v.1.3.0; https://dalexander.github.io/admixture) was used to estimate ancestry proportions in a set of 3,964 reference samples representing African, European, East Asian, and American ancestries from a dataset of merged genotypes. This included 765 samples of African ancestry from 1000 Genomes (*n* = 661) and HGDP (*n* = 104), 658 samples of European ancestry from 1000 Genomes (*n* = 503) and HGDP (*n* = 155), 727 samples of East Asian ancestry from 1000 Genomes (*n* = 504) and HGDP (*n* = 223), and 1,814 American samples, including 716 Indigenous Mexican samples from the MAIS study, 64 admixed Mexican American samples from MXL, 21 Maya and 13 Pima samples from HGDP, and 1,000 unrelated Mexican samples from the MCPS. Included SNPs were limited to variants present on the Illumina GSA v.2 genotyping array for which TOPMed-imputed variants in the MAIS study had information *r*^*2*^ ≥ 0.9 (*m* = 199,247 SNPs). To select the optimum number of ancestry populations (*K*) to include in the admixture model, fivefold cross validation was performed for each *K* in the set 4 to 25 with the –cv flag. To obtain ancestry proportion estimates in the remaining set of 137,511 MCPS samples, the population allele frequencies (*P*) estimated from the analysis of reference samples were fixed as parameters so that the remaining samples could be projected into the admixture model. Projection was performed for the *K* = 4 model and for the *K* = 18 model that produced the lowest cross-validation error, and point estimation was attained using the block relaxation algorithm.

### External datasets used in genetic analyses

The MAIS genotyping datasets were obtained from L. Orozco from Insituto Nacional de Medicina Genómica. For 644 samples, genotyping was performed using an Affymetrix Human 6.0 array (*n* = 599,727 variants). An additional 72 samples (11 ancestry populations) were genotyped using an Illumina Omni 2.5 array (*n* = 2,397,901 variants). The set of 716 Indigenous samples represent 60 of out the 68 recognized ethnic populations in Mexico^3^. Per chromosome, VCFs for each genotyping array were uploaded to the TOPMed imputation server (https://imputation.biodatacatalyst.nhlbi.nih.gov) and imputed from a multi-ethnic reference panel of 97,256 whole genomes. Phasing and imputation were performed using the programs eagle and MiniMac, respectively. The observed coefficient of determination (*r*^*2*^) for the reference allele frequency between the reference panel and the genotyping array was 0.696 and 0.606 for the Affymetrix and Illumina arrays, respectively.

Physical positions of imputed variants were mapped from genome build GRCh37 to GRCh38 using the program LiftOver, and only variant positions included on the Affymetrix GSA v.2 were retained. After further filtering out variants with imputation information *r*^*2*^ < 0.9, the following QC steps were performed before merging of the MAIS Affymetrix and Illumina datasets: (1) removal of ambiguous variants (that is, A/T and C/G polymorphisms); (2) removal of duplicate variants; (3) identifying and correcting allele flips; and (4) removal of variants with position mismatches. Merging was performed using the --bmerge command in Plink (v.1.9).

We used publicly available genotypes from the HGDP (*n* = 929) and the 1000 Genomes project (*n* = 2,504). To obtain a combined global reference dataset for downstream analyses of population structure, admixture and local ancestry, the HGDP and 1000 Genomes datasets were merged. The resulting merged public reference dataset was subsequently merged with the MAIS dataset and MCPS genotyping array dataset. Each merge was performed using the –bmerge function in Plink (v.1.9; https://www.cog-genomics.org/plink) after removing ambiguous variants, removing duplicate variants, identifying and correcting allele flips, and removing variants with position mismatches. The combined global reference dataset comprised 199,247 variants and 142,660 samples.

### LAI

To characterize genetic admixture within the MCPS cohort, we performed a seven-way LAI analysis with RFMix (v.2.0; https://github.com/slowkoni/rfmix) that included reference samples from the HGDP and 1000 Genomes studies, and Indigenous samples from the MAIS study. This merged genotyping dataset of samples across these studies with the 138,511 MCPS participants included 204,626 autosomal variants and 5,363 chromosome X variants.

To identify reference samples with extensive admixture to exclude from LAI, we performed admixture analysis with the program TeraSTRUCTURE (https://github.com/StoreyLab/terastructure) on a merged genotyping dataset (*n* = 3,274) that included African (AFR), European (EUR) and American (AMR) samples from the HGDP, 1000 Genomes and MAIS studies, and 1,000 randomly selected unrelated MCPS samples. Following the recommended workflow in the TeraSTRUCTURE documentation (https://github.com/StoreyLab/terastructure), we varied the rfreq parameter from the set of {0.05, 0.10, 0.15, 0.20} of autosomal variants with *K* = 4 and selected the value that maximized the validation likelihood (20% of autosomal variants; rfreq = 45,365). We then varied the *K* parameter and ran it in triplicate to identify the value that attained a maximal average validation likelihood (*K* = 18). Each of the estimated *K* ancestries was assigned to a global ‘superpopulation’ (that is, AFR, EUR and AMR), and the cumulative *K* ancestry proportion was used as an ancestry score for selecting reference samples. Using an ancestry score threshold of ≥0.9, 666 AFR, 659 EUR and 616 AMR samples were selected as reference samples. The AMR samples used for seven-way LAI comprised 98 Mexico_North, 42 Mexico_Northwest, 185 Mexico_Central, 128 Mexico_South and 163 Mexico_Southeast individuals.

Reference samples were phased using Shapeit (v.4.1.2; https://odelaneau.github.io/shapeit4) with default settings, and the phasing of the 138,511 MCPS participants was performed as described above (see the section ‘Array phasing’). Seven-way LAI was performed using RFMix (v.2.0), with the number of terminal nodes for the random forest classifier set to 5 (-n 5), the average number of generations since expected admixture set to 15 (-G 15), and ten rounds of expectation maximization (EM) algorithm (-e 10). Global ancestry proportion estimates were derived by taking the average per-chromosome *Q* estimates (weighted by chromosome length) for each of the seven ancestries (that is, AFR, EUR, Mexico_North, Mexico_Northwest, Mexico_Central, Mexico_South and Mexico_Southeast). Inferred three-way global ancestry proportion estimates were obtained by combining proportions for each of the five Indigenous Mexican populations into a single ‘AMR’ category.

To delineate local ancestry segments for use in the estimation of ancestry-specific allele frequencies (see the section ‘Ancestry-specific allele frequency estimation’), we performed a three-way LAI analysis using a merged genotyping dataset that excluded the MAIS samples as this afforded greater genotyping density (493,036 autosomal variants and 12,798 chromosome X variants). Before LAI analysis, reference samples were selected using the same workflow for TeraSTRUCTURE as described above, with modifications being the inclusion of 10,000 unrelated MCPS participants and an ancestry threshold of 0.95. RFMix was applied as described above, with modifications being the use of 753 AFR, 649 EUR and 91 AMR reference samples, specification of 5 rounds of EM (-e 5), and use of the --reanalyze-reference option, which treated reference haplotypes as if they were query haplotypes and updated the set of reference haplotypes in each EM round.

To measure the correlation in ancestry between partner pairs, we used a linear model to predict ancestry of each partner using the ancestry of their spouse, education level (four categories) and district (Coyoacán and Iztapalapa) of both partners.

### Testing departures from global ancestry proportions

We averaged local ancestry dosages (estimated using RFMix at 98,012 positions along the genome) from 78,833 unrelated MCPS samples and performed a per-ancestry scan testing for deviation of local ancestry proportion from the global ancestry proportion^19^. The test is based on assumptions of binomial sampling and normal approximation for the sample mean. The global ancestry proportion for each ancestry was estimated as a robust average over local ancestry using the Tukey’s biweight robust mean. The scan was performed in all autosomes separately for African, European and Indigenous Mexican ancestries with the significance threshold 1.7 × 10^–7^ = 0.05/(98, 012 × 3), which accounts for the number of local ancestry proportions tested and the three ancestries.

### Fine-scale population structure based on IBD sharing

IBD segments from hapIBD were summed across pairs of individuals to create a network of IBD sharing represented by the weight matrix $W\in {{\mathbb{R}}}_{\ge 0}^{n\times n}$ for *n* samples. Each entry ${w}_{{ij}}\in W$ gives the total length in cM of the genome that individuals *i* and *j* share identical by descent. We sought to create a low-dimensional visualization of the IBD network. We used a similar approach to that described in ref. ^14^, which used the eigenvectors of the normalized graph Laplacian as coordinates for a low-dimensional embedding of the IBD network. Let *D* be the degree matrix of the graph with ${d}_{{ii}}=\sum _{{j}}{w}_{{ij}}$ and 0 elsewhere. The normalized (random walk) graph Laplacian is defined to be $L=I-{D}^{-1}W$, where *I* is the identity matrix.

The matrix *L* is positive semi-definite, with eigenvalues $0={\lambda }_{0}\le {\lambda }_{1}\le \cdots \le {\lambda }_{n-1}$. The multiplicity of eigenvalue 0 is determined by the number of connected components in the IBD network. If *L* is fully connected, the eigenvector associated with eigenvalue 0 is constant, whereas the remaining eigenvectors can be used to compute a low-dimensional representation of the IBD network. If *p* is the desired dimension, and *u*_1_,…, *u*_*p*_ the bottom 1…*p* eigenvectors of *L* (indexed from 0), the matrix $U\in {{\mathbb{R}}}^{n\times p}$ with columns *u*_1_,…, *u*_*p*_ define a low-dimensional representation of each individual in the IBD network^39^. In practice, we solved the generalized eigenvalue problem to obtain *u*_1_,…, *u*_*p*_.

$${Wu}=\mu {Du}$$

If *u* is an eigenvector of *L* with eigenvalue *λ*, then *u* solves the generalized eigenvalue problem with eigenvalue 1 – *λ*.

To apply to the IBD network of the MCPS cohort, we first removed edges with weight >72 cM as previously done^14^. We did this to avoid the influence on extended families on the visualization. We next extracted the largest connected component from the IBD network, and computed the bottom *u*_1_,…, *u*_20_ eigenvectors of the normalized graph Laplacian.

### Fine-scale population structure based on haplotype sharing

To examine fine-scale population structure using haplotype sharing, we calculated a haplotype copying matrix *L* using Impute5 (https://jmarchini.org/software/#impute-5) with entries *L*_*ij*_ that are the length of sequence individual *i* copies from individual *j*. Impute5 uses a scalable imputation method that can handle very large haplotype reference panels. At its core is an efficient Hidden Markov model that can estimate the local haplotype sharing profile of a ‘target’ haplotype with respect to a ‘reference’ set of haplotypes. To avoid the costly computations of using all the reference haplotypes, an approach based on the PBWT data structure was used to identify a subset of reference haplotypes that led to negligible loss of accuracy. We leveraged this methodology to calculate the copying matrix *L*, using array haplotypes from a set of 58,329 unrelated individuals as both target and reference datasets, and used the --ohapcopy –ban-repeated-sample-names flags to ban each target haplotype being able to copy itself. SVD on a scaled centred matrix was performed using the bigstatsr package (https://cran.r-project.org/web/packages/bigstatsr/index.html) to generate 20 PCs. This is equivalent to an eigen-decomposition of the variance-covariance matrix of recipients’ shared segment lengths.

### Imputation experiments

We imputed the filtered array dataset using both the MCPS10k reference panel and the TOPMed imputation server. For TOPMed imputation, we used Plink2 to convert this dataset from Plink1.9 format genotypes to unphased VCF genotypes. For compatibility with TOPMed imputation server restrictions, we split the samples in this dataset into six randomly assigned subsets of about 23,471 samples, and into chromosome-specific bgzipped VCF files. Using the NIH Biocatalyst API (https://imputation.biodatacatalyst.nhlbi.nih.gov), we submitted these six jobs to the TOPMed imputation server. Following completion of all jobs, we used bcftools merge to join the resulting dosage VCFs spanning all samples. For the MCPS10k imputation, we used Impute5 (v.1.1.5). Each chromosome was split into chunks using the imp5Chunker program with a minimum window size of 5 Mb and a minimum buffer size of 500 kb. Information scores were calculated using qctool (https://www.well.ox.ac.uk/~gav/qctool_v2/).

The 1000 Genomes WGS genotype VCF files were downloaded (http://ftp.1000genomes.ebi.ac.uk/vol1/ftp/data_collections/1000G_2504_high_coverage/working/20201028_3202_phased/) and filtered to remove sites that are multi-allelic sites, duplicated, have missingness >2%, Hardy–Weinberg *P* < 1 × 10^–8^ in any subpopulation and MAF < 0.1% in any subpopulation. We used only those 490 AMR samples in the MXL, CLM, PUR and PEL subpopulations. We constructed two subsets of genotypes on chromosome 2 from the Illumina HumanOmniExpressExome (8.v1-2) and Illumina GSA (v.2) arrays, and these were used as input to the TOPMed and MCPS10k imputation pipelines.

We measured imputation accuracy by comparing the imputed dosage genotypes to the true (masked) genotypes at variants not on the arrays. Markers were binned according to the MAF of the marker in 490 AMR samples. In each bin, we report the squared correlation (*r*^2^) between the concatenated vector of all the true (masked) genotypes at markers and the vector of all imputed dosages at the same markers. Variants that had a missing rate of 100% in the WGS dataset before phasing were removed from the imputation assessment.

### Ancestry-specific allele frequency estimation

The LAI results consist of segments of inferred ancestry across each haplotype of the phased array dataset. As the WES and WGS alleles were phased onto the phased array scaffold, we inferred the ancestry of each exome allele using interpolation from the ancestry of the flanking array sites. For each WES and WGS variant on each phased haplotype, we determined the RFMix ancestry probability estimates at the two flanking array sites and used their relative base-pair positions to linearly interpolate their ancestry probabilities. For a given site, if ${p}_{{ijk}}$ is the probability that the *j*th allele of the *i*th individual is from population *k*, and *G*_*ij*_ is the 0/1 indicator of the non-reference allele for the *j*th allele of the *i*th individual then the weighted allele count (AC_*k*_), the weight allele number (AN_*k*_) and the allele frequency (*θ*_*k*_) of the *k*th population is given by

$${{\rm{AC}}}_{k}=\mathop{\sum }\limits_{i=1}^{n}\mathop{\sum }\limits_{j=1}^{2}{p}_{ijk}{G}_{ij},\,{{\rm{AN}}}_{k}=\mathop{\sum }\limits_{i=1}^{n}\mathop{\sum }\limits_{j=1}^{2}{p}_{ijk},\,{\theta }_{k}=\frac{{{\rm{AC}}}_{k}}{{{\rm{AN}}}_{k}}$$

An estimate of the effective sample size for population *k* at the site is ${n}_{k}={{\rm{AN}}}_{k}/2$. Singleton sites can be hard to phase using existing methods. Family information and phase information in sequencing reads was used in the WGS phasing, and this helped to phase a proportion of the singleton sites. In the WES dataset, we found that 46% of exome singletons occurred in stretches of heterozygous ancestry. For these variants, we gave equal weight to the two ancestries when estimating allele frequencies.

To validate the MCPS allele frequencies, we downloaded the gnomAD v.3.1 reference dataset (https://gnomad.broadinstitute.org) and retained only high-quality variants annotated as passed QC (FILTER=”PASS”), SNVs, outside low-complexity regions and with the number of called samples greater than 50% of the total sample size (*n* = 76,156). We additionally overlapped gnomAD variants with TOPMed Freeze 8 high-quality variants (FILTER=”PASS”) (https://bravo.sph.umich.edu/freeze8/hg38). We further merged gnomAD variants and MCPS exome variants by the chromosome, position, reference allele and alternative allele names and excluded MCPS singletons, which were heterozygous in ancestry. This process resulted in 2,249,986 overlapping variants available for comparison with the MCPS WES data. Median sample sizes in gnomAD non-Finish Europeans, African/Admixed African and Admixed American populations were 34,014, 20,719 and 7,639, respectively.

### Using IBD segments to compute relatedness-corrected allele frequencies

To investigate the effect of relatedness on allele frequency estimates, we implemented a method to compute relatedness-corrected allele frequencies using identical-by-descent (IBD) segments. This method computes allele frequencies at a locus by clustering alleles inherited IBD from a common ancestor, then counting alleles once per common ancestor rather than once per sample. Because IBD sharing is affected by both demography and relatedness, we limited IBD sharing to segments between third-degree relatives or closer. Conceptually, this is equivalent to tracing the genealogy of a locus back in time across all samples until no third-degree relatives remain, then computing allele frequencies in the ancestral sample.

We estimated allele frequencies in two steps. First, we constructed a graph based on IBD sharing at a locus. Second, we estimated allele counts and allele numbers by counting the connected components of the IBD graph. Our approach is similar to the DASH haplotype clustering approach^40^. However, we make different assumptions about how errors affect the IBD graph and additionally compute ancestry-specific frequencies using local ancestry inference estimates.

To construct the IBD graph, suppose we have genotyped and phased *N* diploid samples at *L* biallelic loci. For each locus *l* we construct an undirected graph *G*_*l*_ = (*V*_*l*_,*E*_*l*_) describing IBD sharing among haplotypes. Let the tuple (*i*, *j*)_*l*_ represent haplotype *j* of sample *i* at locus *l*, and let ${h}^{{\left(i,j\right)}_{l}}\in \{\mathrm{0,1}\}$ be the allele itself. Define

$$\begin{array}{l}{V}_{l}\,=\,\{{(i,j)}_{l}:{\rm{for}}\,1\le j\le 2\,{\rm{and}}\,1\le i\le N\}\\ {E}_{l}\,=\,\{({(i,j)}_{l},{(s,t)}_{l}):{h}^{{(i,j)}_{l}}\,{\rm{and}}\,{h}^{{(s,t)}_{l}}\,{\rm{are}}\,{\rm{IBD}}\}.\end{array}$$

In words, the set of vertices *V* constitute all haplotypes at locus *l*. Each edge in *E* is between a pair of haplotypes that fall on the same IBD segment (Supplementary Fig. 25).

If IBD segments are observed without error, then each maximal clique of *G*_*l*_ represents a set of haplotypes descended from a common ancestor. In practice, edges will be missing owing to errors in IBD calling. Thus, what we observe are sets of connected components rather than maximal cliques. Because we limited edges to pairs of third-degree relatives or closer, we assumed missing edges in connected components are false negatives and included them. We additionally removed edges between haplotypes for which the observed alleles conflicted.

Given an IBD graph *G*_*l*_ = (*V*_*l*_, *E*_*l*_) for a locus *l*, we estimated alternative allele counts and allele numbers by counting the connected components of the graph. Let *C*_*l*1_,…,*C*_*lm*_ be the connected components of *G*_*l*_. Let *C*_ALT_ = {*C*_*im*_: haplotypes in *C*_*im*_ have the ALT allele} and *C*_REF_ = {*C*_im_: haplotypes in *C*_*im*_ have the REF allele}

Then

$$\begin{array}{l}AC=| {C}_{{\rm{ALT}}}| \\ AN=| {C}_{{\rm{ALT}}}| +| {C}_{{\rm{REF}}}| \\ AF=AC\,/\,AN\end{array}$$

We additionally used LAI estimates to compute ancestry-specific frequencies. Let ${p}^{{(i,j)}_{l}}\in {{\mathbb{R}}}^{K}$ be the vector of probabilities that an allele on haplotype *j* from sample *i* at locus *l* comes from one of *K* populations. For each connected component, we averaged local ancestry estimates

$${\bar{p}}_{{C}_{im}}=\frac{1}{|{C}_{lm}|}{\sum }_{{(i,j)}_{l}\in {C}_{lm}}{p}^{{(i,j)}_{l}}$$

We computed a vector of weighted allele counts *W* and allele numbers *N* by

$$\begin{array}{l}W={\sum }_{C\in {C}_{{\rm{ALT}}}}{\bar{p}}_{C}\\ N={\sum }_{C\in {C}_{{\rm{ALT}}}}{\bar{p}}_{C}+{\sum }_{C\in {C}_{{\rm{REF}}}}{\bar{p}}_{C}\end{array}$$

Ancestry-specific frequencies were estimated by dividing each component of *W* by the corresponding component of *N*.

For singletons for which the phasing of haplotypes was unknown, we averaged local ancestry estimates from haplotypes in the sample.

### Generation of PRS values for BMI

To generate source datasets for assessing trans-ancestry portability of BMI PRS, whole genome regression was performed using Regenie (https://rgcgithub.github.io/regenie/) in individuals in the MCPS and in a predominantly European-ancestry cohort from the UK Biobank. Individuals with type 2 diabetes (ICD10 code E11 or self-reported) were excluded. BMI values underwent rank-based inverse normal transformation (RINT) by sex and ancestry; models were additionally adjusted for age, age^2^ and technical covariates (UK Biobank). The Regenie summary statistics from the UK Biobank were used to generate a BMI PRS in MCPS; conversely, MCPS summary statistics were applied to UK Biobank statistics.

To avoid overfitting with respect to selection of a PRS algorithm and its associated tuning parameters, LDpred (https://github.com/bvilhjal/ldpred) with *ρ* value of 1 was chosen from a recent publication of BMI and obesity^27^. Summary statistics were restricted to HapMap3 variants and followed existing filtering recommendations. In the MCPS, two PRS values were generated; imputed variants were obtained from the MCPS10k reference panel or the TOPMed panel. In the UK Biobank data, PRS values were calculated separately by continental ancestry (African, East Asian, European, Latino, South Asian), determined from a likelihood-based inference approach^8^ in a merged dataset of variants from UK Biobank and the 1000 Genomes project.

To evaluate PRS performance, BMI values were transformed (RINT) by sex and ancestry and regressed on PRS, age and age^2^. As for the generation of summary statistics, individuals with diabetes were excluded from the analysis. PRS accuracy was assessed by incremental *R*^2^ (proportional reduction in regression sum of squares error between models with and without BMI PRS). Additionally, raw BMI values with PRS, age, age^2^, sex and ancestry were modelled to obtain per BMI PRS standard deviation effect-size estimates. The impact of ancestry differences on source summary statistics compared to target PRS was assessed with two approaches. For the MCPS, individuals were divided into quantiles by estimated Indigenous Mexican Ancestry using the LAI approach described above. For the UK Biobank, metrics were calculated within each 1000 Genomes-based continental ancestry.

### Ethics and inclusion

The MCPS represents a long-standing scientific collaboration between researchers at the National Autonomous University of Mexico and the University of Oxford, who jointly established the study in the mid-1990s and have worked together on it ever since. Blood sample collection and processing were funded by a Wellcome Trust grant to the Mexican and Oxford investigators. However, at the time, no funding was requested to create an appropriate long-term sample storage facility in Mexico City. Therefore, the Mexican investigators agreed for the samples to be shipped to Oxford where they could be stored in a liquid-nitrogen sample storage archive (funded by the UK Medical Research Council and Cancer Research UK) that had previously been established by the Oxford team, and only on the understanding that control of the samples remained with the Mexican investigators. The shipping of blood samples from Mexico to the United Kingdom was approved by the Mexican Ministry of Health, and the study was approved by scientific and ethics committees within the Mexican National Council of Science and Technology (0595 P-M), the Mexican Ministry of Health and the Central Oxford Research Ethics Committee (C99.260). Although appropriate facilities in Mexico City now exist to store the samples, the Mexican investigators have decided that the costs of sending them back to Mexico exceed the benefits of having closer access to them. Study participants gave signed consent in keeping with accepted ethical practices at the time for observational cohort studies. The baseline consent form stated that their blood samples would be stored and used in the future for unspecified research purposes (with a specific statement that this would include future analysis of genetic factors) and that it would probably be many years before such blood analyses were done. The MCPS consent form also stated that the research was being done in collaboration with the University of Oxford and that the purpose of the study was to benefit future generations of Mexican adults. In 2019, the Mexican and Oxford investigators jointly agreed to allow the extracted DNA to be sent to the Regeneron Genetics Center after they had offered to genotype and exome sequence the entire cohort—thereby creating the resource now available for future research by Mexican scientists (see the ‘Data Availability’ section)—in exchange for sharing the other data with them for the purpose of performing joint collaborative genetic analyses. Formal approval to share MCPS data with commercial institutions was sought and obtained from the Medical Ethics Committee of the National Autonomous University of Mexico (FMED/CEI/MHU/001/2020). Major discoveries from the study have been disseminated through open-access scientific publications, local and international scientific meetings, press releases, social media and local television, but direct communication of study results to the original study participants is unfortunately not practical as no information on telephone numbers or email addresses was collected at recruitment. As in other prospective cohort studies (such as the UK Biobank), it was agreed that there would be no feedback of individual blood results to participants, as it has been shown that such feedback can do more harm than good (whereas no feedback ensures that that is not the case).

Recruitment of individuals in the MAIS cohort was done with approval of the leaders of the Indigenous communities and with the support of the National Commission for the Development of Indigenous Communities of Mexico (CDI), now the Instituto Nacional de los Pueblos Indígenas (INPI). All participants provided written informed consent, and authorities or community leaders participated as translators where necessary. The consent form described how findings from the study may have commercial value and be used by for-profit companies. Sample collection for MAIS was approved by the Bioethics and Research Committees of the Insituto Nacional de Medicina Genómica in Mexico City (protocol numbers 31/2011/I and 12/2018/I). Preliminary data from the MAIS cohort have been discussed with the Indigenous leaders and volunteer individuals included in the study, explaining the meaning of the findings on health or population’s history, and the potential use of the data in future collaborations.

### Reporting summary

Further information on research design is available in the Nature Portfolio Reporting Summary linked to this article.

## Online content

Any methods, additional references, Nature Portfolio reporting summaries, source data, extended data, supplementary information, acknowledgements, peer review information; details of author contributions and competing interests; and statements of data and code availability are available at 10.1038/s41586-023-06595-3.

### Supplementary information

Supplementary Figures Supplementary Figs. 1–28.

Reporting Summary

Supplementary Tables Supplementary Tables 1–31.

## Figures and Tables

**Extended Data Table 1:**
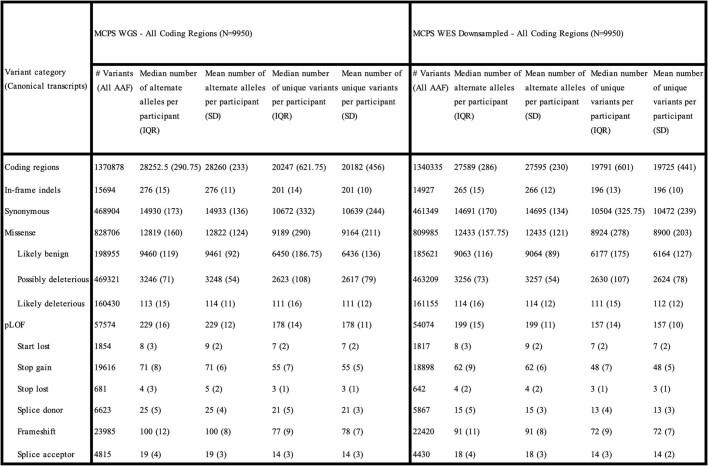
Comparison of WES and WGS datasets in coding genes

Variants were annotated with VEP. Predicted function is defined by canonical transcript consequence in Ensembl v100. Counts are restricted to the same set of 9,950 individuals with both WGS and WES available. All variants passed QC for the respective platform. AAF = Alternate Allele Frequency, IQR = Inter Quartile Range, SD = Standard Deviation.

**Extended Data Table 2:**
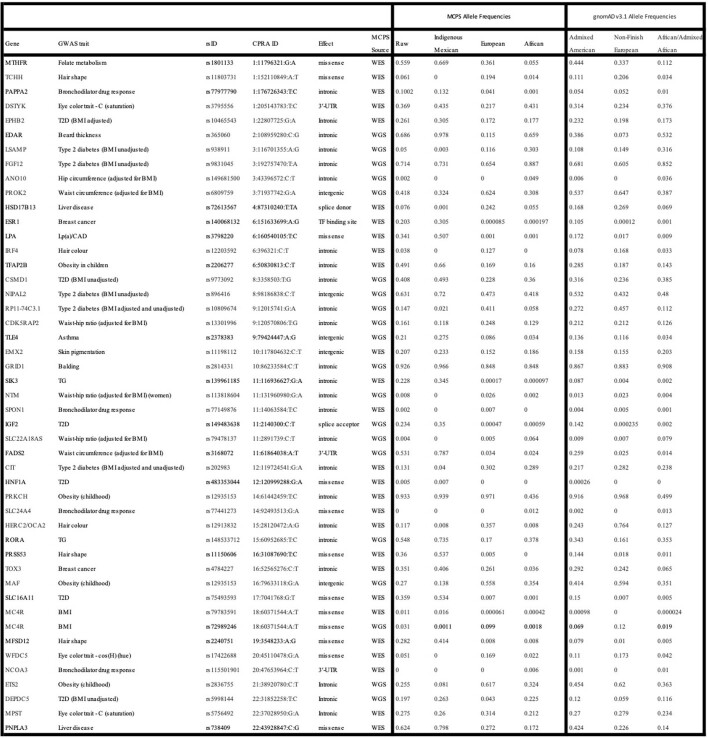
Ancestry-specific allele frequencies at GWAS loci previously reported in studies of Mexican and Latin/Central American populations

MCPS Indigenous Mexican, European and African allele frequencies, estimated in MCPS WES/WGS data using our deconvolution approach, are reported together MCPS Raw allele frequencies calculated directly on raw MCPS data. Allele frequencies for three relevant population groups available in gnomAD 3.1 are added for comparison.

## Data Availability

On 1 May 2023 the genetic data from the MCPS—including the genotype array data, TOPMed-imputed data, exome data, WGS data and MCPS imputation panel files—were made available for sharing with bona fide academic researchers in Mexico through access to a DNAnexus research analysis platform powered by Amazon Web Services. Researchers in Mexico who are interested in obtaining these and/or the non-genetic data for specific academic research purposes, or in collaborating with MCPS investigators on a specific research proposal, should first visit the study’s Oxford-hosted webpage (http://www.ctsu.ox.ac.uk/research/mcps) to download the Data and Sample Access Policy in English or Spanish. The non-genetic data available for sharing may be reviewed on the study’s online Data Showcase (https://datashare.ndph.ox.ac.uk/mexico). The Data and Sample Access Policy aims to promote equity in research by giving preferential access to researchers in Mexican institutions whereby such applicants have free access with a period of exclusivity over researchers in other parts of the world (although principal investigators in Mexico may still choose to collaborate with researchers in other parts of the world on their approved projects if they wish). Researchers in Mexican institutions are also provided with analysis ‘credits’ to cover the cost of running their analyses on the platform and downloading their results. For academic researchers in other parts of the world, the genetic data will be made available for open-access sharing only after the end of the exclusivity period for Mexican researchers (the duration of which is constantly reviewed but in no circumstances will exceed 2 years). Researchers in high-income countries will be required to pay a nominal data-access fee (to cover the administrative costs associated with processing data requests and maintaining the data analysis platform), but there will be no data access fee for researchers in low- or middle-income countries. The reason for giving Mexican researchers preferential access to the data generated in Mexico is to foster equity and provide an opportunity to develop local research capacity. Otherwise, given the disproportionate analytic capacity in, for example, North America and Western Europe, as compared with Mexico, there is a risk that future analyses of these data will be dominated by researchers from outside Mexico. The MCPS ancestry-specific allele frequencies are available in a public browser that includes options for direct download (https://rgc-mcps.regeneron.com/). The GRCh38 reference accession code is available from the NCBI website at https://www.ncbi.nlm.nih.gov/datasets/genome/GCF_000001405.26/.

## Code availability

Code developed for the analysis of this paper is available from two separate GitHub repositories at https://github.com/mcps-analysts/mcps-genetic-cohort-profile and https://github.com/rgcgithub/mcps_ibd_freq_calc.
