# Author Correction: Genotyping, sequencing and analysis of 140,000 adults from Mexico City

**DOI:** 10.1038/s41586-024-07051-6

**Published:** 2024-02-08

**Authors:** Andrey Ziyatdinov, Jason Torres, Jesús Alegre-Díaz, Joshua Backman, Joelle Mbatchou, Michael Turner, Sheila M. Gaynor, Tyler Joseph, Yuxin Zou, Daren Liu, Rachel Wade, Jeffrey Staples, Razvan Panea, Alex Popov, Xiaodong Bai, Suganthi Balasubramanian, Lukas Habegger, Rouel Lanche, Alex Lopez, Evan Maxwell, Marcus Jones, Humberto García-Ortiz, Raul Ramirez-Reyes, Rogelio Santacruz-Benítez, Abhishek Nag, Katherine R. Smith, Amy Damask, Nan Lin, Charles Paulding, Mark Reppell, Sebastian Zöllner, Eric Jorgenson, William Salerno, Slavé Petrovski, John Overton, Jeffrey Reid, Timothy A. Thornton, Gonçalo Abecasis, Jaime Berumen, Lorena Orozco-Orozco, Rory Collins, Regeneron Genetics Center, Gonçalo Abecasis, Adolfo Ferrando, Michael Cantor, Giovanni Coppola, Andrew Deubler, Aris Economides, Katia Karalis, Luca A. Lotta, Lyndon J. Mitnaul, John D. Overton, Jeffrey G. Reid, Alan Shuldiner, Katherine Siminovitch, Christina Beechert, Erin D. Brian, Laura M. Cremona, Hang Du, Caitlin Forsythe, Zhenhua Gu, Kristy Guevara, Michael Lattari, Alexander Lopez, Kia Manoochehri, Manasi Pradhan, Raymond Reynoso, Ricardo Schiavo, Maria Sotiropoulos Padilla, Chenggu Wang, Sarah E. Wolf, Amelia Averitt, Nilanjana Banerjee, Dadong Li, Sameer Malhotra, Justin Mower, Mudasar Sarwar, Deepika Sharma, Jeffrey C. Staples, Jay Sundaram, Sean Yu, Aaron Zhang, Mona Nafde, George Mitra, Sujit Gokhale, Andrew Bunyea, Janice Clauer, Krishna Pawan Punuru, Sanjay Sreeram, Gisu Eom, Benjamin Sultan, Vrushali Mahajan, Eliot Austin, Koteswararao Makkena, Sean O’Keeffe, Tommy Polanco, Ayesha Rasool, William J. Salerno, Lance Zhang, Boris Boutkov, Evan Edelstein, Alexander Gorovits, Ju Guan, Alicia Hawes, Olga Krasheninina, Adam J. Mansfield, Evan K. Maxwell, Suying Bao, Kathie Sun, Chuanyi Zhang, Manuel Allen Revez Ferreira, Kathy Burch, Adrian Campos, Lei Chen, Sam Choi, Liron Ganel, Sheila Gaynor, Benjamin Geraghty, Akropravo Ghosh, Salvador Romero Martinez, Christopher Gillies, Lauren Gurski, Joseph Herman, Michael Kessler, Jack Kosmicki, Adam Locke, Priyanka Nakka, Anthony Marcketta, Arden Moscati, Aditeya Pandey, Anita Pandit, Jonathan Ross, Carlo Sidore, Eli Stahl, Maria Suciu, Peter VandeHaar, Sailaja Vedantam, Scott Vrieze, Rujin Wang, Kuan-Han Wu, Bin Ye, Blair Zhang, Olivier Delaneau, Maya Ghoussaini, Jingning Zhang, Brian Hobbs, Jon Silver, William Palmer, Rita Guerreiro, Jan Freudenberg, Amit Joshi, Antoine Baldassari, Cristen Willer, Sarah Graham, Jonas Bille Nielsen, Mary Hass, Niek Verweij, George Hindy, Jonas Bovijn, Tanima De, Parsa Akbari, Luanluan Sun, Olukayode Sosina, Arthur Gilly, Peter Dornbos, Juan Rodriguez-Flores, Moeen Riaz, Manav Kapoor, Gannie Tzoneva, Momodou W. Jallow, Anna Alkelai, Ariane Ayer, Veera Rajagopal, Sahar Gelfman, Vijay Kumar, Jacqueline Otto, Neel Parikshak, Aysegul Guvenek, Jose Bras, Silvia Alvarez, Jessie Brown, Jin He, Hossein Khiabanian, Marcus B. Jones, Esteban Chen, Jaimee Hernandez, Michelle G. LeBlanc, Jason Mighty, Nirupama Nishtala, Nadia Rana, Jennifer Rico-Varela, Mexico City Prospective Study, Jonathan R. Emberson, Richard Peto, Abraham Garduño-Martinez, Abril Garcia-Lopez, Adrian Abarca-Cardoso, Adriana Caballero-Mondragon, Adriana Gutierrez-Parra, Adriana Leticia Diaz-Avila, Alan Emiliano Bautista-Hernandez, Alberto Méndez-Villalba, Aldo Shaid Ramos-Hernandez, Alejandra Alejo-Salazar, Alejandra Angelica Perez-Moncada, Alejandra Martinez, Alejandra Peralta-Gallardo, Alejandro Flores-Magana, Alfa Izamar Benitez-Garcia, Alicia González-Castillo, Alicia Villegas-Esparza, Alma Delia Morales-Bravo, Alma Fernanda Mora-Negrete, Alma Hernandez-Galicia, Alma Rosa Arenas-García, Alma Rosa Valentin-Martinez, Amalia Paredes-Rojas, Ambar Nayeli Flores-Sanchez, Amelia Ortiz-Jaen, America Juarez-Salazar, América Victoria Cervantes-Torres, Amparo Luviano-Martínez, Ana del Carmen Alejandro-Perez, Ana Dominguez-Alvarado, Ana Isabel Fuentes-Alvarado, Ana Karen Arreola-Olvera, Ana Laura Bautista-Sanchez, Ana Lilia Enríquez-Álvarez, Ana Lilia Reynoso-Valverde, Ana María Isidro-Cid, Ana Montserrat Lechuga-Mendoza, Andrea Esquivel-Mejía, Andrea Galvino-Antonio, Andrea Gomez-Luna, Andres Martinez-Martinez, Anel Aragón-Domínguez, Angelica Gamboa-Romero, Angelica Guerrero, Angelica Ruiz-Hernandez, Antonia González-María, Araceli Martínez-Santana, Araceli Rojas-Vásquez, Arcelia Rojas-Santamaría, Armida Sánchez-Corral, Athzin Berenice Rosas-Avila, Beatriz Cruz-Acevedo, Beatriz Gonzalez-Ibañes, Beatriz Rojas, Beatriz Velázquez-Mancilla, Belen Escalona-Franco, Bernardo Ochoa-Morales, Braulio Rivera-Cortés, Brenda Castañeda-Gazpar, Brenda J. Calderon-Garcia, Brenda Jimena Jimenez-Gutierrez, Brian Orlando Sanchez-Martin, Carlos Alberto Toxqui-Rico, Carlos Antonio Clemente-Montano, Carlos Daniel Jimenez-Gutierrez, Casandra Alvarez-Meneses, Catalina Gasca-Velázquez, Cecilia Luna-Barroso, César Marín-Pérez, Cinthia Calderon-Camacho, Cinthia Hernandez-Perez, Cinthia Xóchitl Hernández-Peralta, Clarinet Castillo-Rioja, Claudia Bustamante-Durán, Claudia Elizabeth Espinosa-Quintana, Claudia Lilia Galicia-Flores, Claudia Lizbeth Villagomez-Piña, Cynthya Berenice Sierra-Martinez, Daniel Fernández-Corona, Daniel Ordaz-Jiménez, Daniela Oreli Hernandez-Castillo, Daniela Ramirez-Aranda, Dante Zazhil Lopez-Guzman, Diana del Monte-Homobono, Diana Isabel Gonzalez-Enciso, Diana Laura Bolanos-Hernandez, Edith Elizabeth Valdez-Solano, Edith Gonzalez-Torres, Edson Alfonso Mercado-Hernández, Eduardo Alvarado-Valle, Elisa Morales-Martinez, Elizabet Gonzalez, Elsa Yadira Díaz-Martínez, Elvia Isabel Vázquez-Torres, Elvira Ramos-Mendoza, Emiliano del Rio-Gonzalez, Erika Alpizar-Flores, Erika García-García, Erika Pérez-Romero, Esmeralda Sanchez-Martinez, Estefania Perez-Perez, Estela Beatriz López-García, Estela Elisabeth Moran-De Los Santos, Esther Jerónimo-Hernández, Eva María Estefes-Hernández, Evelin Sanchez-Alvarez, Felipe de Jesus Ramirez-Tinajero, Felipe Rivera-Cortés, Francisca Ana Yetzy Lopez-Tellez, Francisco Barajas-Soto, Francisco Javier Garcia-Gonzalez, Francisco Javier Ruvalcaba-López, Gabriel Enrique Jimenez-Vasquez, Gabriela López-Villaseca, Gabriela Paredes-Cruz, Gabriela Rivera-Arredondo, Gardenia Nieto-Valenciano, Genaro Balderas-Martinez, Genoveva Limon, Gerardo Álvarez-Mancilla, Gerardo Fernando Gómez-Dorantes, Gladis Villegas-Ramirez, Gloria Cruz-Angeles, Gloria Hernández-Buendía, Grecia Jimenez-Perez, Guadalupe América Juárez-Salazar, Guadalupe Garduño-Loyola, Hector Hugo Villaseñor-Flores, Hector M. Velasco, Hector Valentin Villanueva-Cervantes, Hectorchavez Mendiola, Hilda Nelly Rodríguez-Neria, Hipatia Lobato-Garcia, Hortencia Torres-Morales, Idith Fabiola Hernández-Peralta, Ingrid Alejandra Ochoa-Ramos, Irais Morales-Casillas, Irene Abuhatab, Irma Garduño-Medina, Irma Palacios-Rivas, Irving Hernandez-Machuca, Irving Israel Ramirez-Ramirez, Isabel Dominguez-Ursula, Isamar Prado-Morales, Israel Adrian Barrios-Custodia, Ivan Abrajan-García, Ivonne Jazmín Aguilar-Flores, Jaime Alfonso Rodriguez-Castro, Jaime Lee Alvarado-Lopez, Jaqueline Guadarrama-Fernández, Jaqueline Lopez-Lopez, Jaredhia Nathaly Pablo-Bautista, Jedini Paola Martinez-Ramirez, Jennifer Mendoza-Mendoza, Jessica Elena Vázquez-Bustamamnte, Joaquín Edmundo Ramírez-Gonzalez, Jorge Hernández-Arellano, Jorge L. Ocana-Monroy, Jorge Ricardo Medina-Torres, Jose Alberto Zavala-Barrera, Jose Cristian Alexis Lemus-Enciso, José Juan Barajas-Gónzalez, José Juan Castañeda-Dorantes, Jose Luis Ocana-Monroy, Josefina Alvarado-Calderón, Josefina Sanchez-Escudero, Joselyn Adali Garcia-Pantoja, Juan Adan Hernandez-Salinas, Juan Carlos Cruz-Hernandez, Juan Carlos Medina-Hernández, Juan Carlos Rodríguez-Ramírez, Juan Gabriel Pérez-Álvarez, Juan Pablo Hernandez-Canales, Juan Rubén Marines-Álvarez, Juana Patricia Romero-Becerril, Julio César Gómez-Dorantes, Julio Ortiz-Sanchez, Karina Adriana Ramos-Perez, Karina Ayala-Escamilla, Karina Sánchez-Ramírez, Karla Patricia Zárate-Barrios, Laura Arroyo-Garfias, Laura Cordoba-Barrios, Laura Limon-Espinoza, Laura Magallón-Nava, Lesley Geraldine Rodriguez-Camacho, Leslie Andrea Avendano-Baltierra, Leslie Nancy Rubio-Rojas, Leticia Cruz-Castañeda, Leticia Martínez-Morales, Lezly Fernanda Arias-Lezama, Lilia Reséndiz-Galván, Liliana Rodríguez-Ayala, Liliana Solano-Vazquez, Lina Velazco-Valdez, Lizbeth Armendáriz-Zahuantitla, Lizbeth Castro, Lucía Torres-Vázquez, Luis Antonio Loa-Orellana, Luis Arturo Vazquez-Padilla, Luis Brandon Toriz-Nava, Luis Ivan Salcedo-Sandoval, Luis Manuel Valdez-Rivera, Luz Xochiquetzalit Morales-Torres, Maciel Areli Camacho-Estrella, Macrina Tapia-Gómez, Magali Abigail Caballero-Sanchez, Magaly Lizbeth Martínez-López, Magdalena Sánchez-Salinas, Marco Antonio Gonzalez-Carranza, Marco Antonio Montes-Mérida, Marco Antonio Salazar-Giron, Margarita Mirta Torres-Rodríguez, María Alejandra Meléndez-Hernández, María Alejandra Ramos-Mendoza, Maria Alexandra Dominguez-Romero, María Antonia-González, María Aurora Pérez-Vargas, María Beatriz Rojas-Aguilar, María Cristina Ruiz-Flores, Maria de los A ngeles Chavez-Corona, María del Carmen Montiel-Pérez, María del Carmen Novelo-Aguilar, María Elena Espinoza-Pérez, María Elena González-Ruiz, María Estela Maya-Colin, Maria Fernanda Kennedy-Vazquez, Maria Hernandez-Soler, María Isabel Medina-Torales, María Olvera-González, Maria Priscila Hernandez-Melendez, María Teresa Villa-Botello, Mariana Andrea Labastida-Luna, Mariana Bolanos-Orduna, Maribel Rodríguez-Ledezma, Marisol Gomez-Collado, Marisol López-Arredondo, Marissa Villa-Ayala, Martha Alvarez-Marin, Martha Decimo-Canales, Martha Flores-Hernández, Martin Flores-Ortiz, Martin Linas-Sanchez, Mauricio Marin-Sanchez, Mayeli Salado-Bazán, Mayra Chagolla-Reyes, Mayranni Marquez-Jimenez, Miguel Angel Martinez-Medina, Miguel Salgado-Martinez, Misael Olivos-Rivera, Moisés Sánchez-Cejudo, Mónica Ernestina Gónzalez-Ramos, Monica Gomez-Abad, Mónica Irineo-Ugarte, Mónica Martínez, Mónica Martínez-Márquez, Nancy Abigail Castillo-Ramos, Nancy Patricia Hernández-Galicia, Natalia Guadalupe Elizarraras-Torres, Natalia Tinoco-Hernandez, Neri Reyna-Salgado, Noé Velázquez-Mandujano, Noemí Zurita-Morán, Norma Alicia Esteban-Cruz, Norma Angelica Orbe-Sierra, Norma Patricia Solís-Calvillo, Oliverio Rivera-Cortez, Omar Santiago-Perez, Oswaldo Hernandez-Camacho, Oswaldo Israel Gómez-Dorantes, Patricia Andrés-Gutiérrez, Patricia Cuarenta-Medina, Patricia Rez, Patricio Marquez-Espino, Paula Morales-Godinez, Paulina Monserrat Montano-Rojas, Ramses Alejandro Bravo-Juarez, Reyna Aurora Garza-Zepeda, Reyna Margarita Contreras-Hernández, Ricardo Manuel Ruiz-Zepeda, Ricardo Marquez-Nunez, Roberto Fabian Pelaez-Granados, Roberto Solera-Calvo, Rocío Hernández-López, Rosalinda García-Anaya, Rosario Dafne Lujan-Velazquez, Rosario Pérez Rul-Rivero, Rosaura Vazquez-Reyes, Rubén Espinoza-Peña, Ruperto García-Pérez, Salomón González-Garrido, Samantha Nayeli-De la Rosa Rodríguez, Sandra Lizbet Colon-Serrano, Sanjuana García-Hernández, Santiago Olvera-Arriaga, Santos Pérez-Gallardo, Sara Heras-Santiago, Sara Yazmin Flores-Jimenez, Sarahi Montiel-Sanchez, Sérgio César Bruno-Baltazar, Sheila Cruz-Martinez, Sibyl Nadir Luna-Ramírez, Silvia Ávila-Jaen, Silvia Cervantes-Saldívar, Socrates Cardenas-Valencia, Sonia Angélica Saldívar-Sánchez, Tania Michelle Sanchez-Damiz, Tomás Dorantes-Rosas, Vera Lopez-Sanchez, Verónica Colín-Hernández, Veronica Perez-Elizalde, Veronica Sanchez-Ortega, Verónica Santos-Sánchez, Veronica Velasco-Nava, Vianey Hernandez-Piña, Violeta Flores-Ramírez, Viridiana Ruiz-Gonzalez, Xiadani Paulina Mejia-Villegas, Xóchitl Cano-Goméz, Yacquelín Mondragón-Martínez, Yamili Evaristo-Montes, Yaquelinne Carcia-Muñoz, Yaxum Mendoza-Rocafuerte, Yazmin Parra-Ortega, Yeni Guadalupe Guadarrama-Fernández, Yojahira Martinez-Morales, Zaira Rebeca Martinez-Vite, Zoraida Lucio-Olmedo, Fernando Rivas-Reyes, Raúl Ramírez-Reyes, Adrián Garcilazo-Ávila, Carlos Gonzáles-Carballo, Diego Aguilar-Ramírez, Doreen Zhu, Eirini Trichia, Erwin Chiquete, Fiona Bragg, Gary Whitlock, Louisa Gnatiuc Friedrichs, Natalie Staplin, Omar Yaxmehen Bello-Chavolla, Richard Haynes, Robert Clarke, Sarah Lewington, William Herrington, Alejandra Vergara, Elizabeth Barrera-Sánchez, Georgina Del Vecchyo-Tenorio, Margarita González-Ruiz, Paulina Baca-Peynado, Tianshu Liu, Yunhe Wang, Adriana Lucrecia Wong y. Wong, Clementina Magos, Fredrik Romer, Herendira Zambrano Martínez, James Wheeler, Kathleen Emmens, Linda Youngman, Martin Radley, Martha Solano Sanchez, Matthew Lacey, Michael R. Hill, Nigel Plunkett, Paul Taylor, Richard Shellard, Sarah Clark, Tim Williams, Gustavo Olaiz Fernandez, Lisa Holland, Malaquias López Cervantes, Aris Baras, Michael R. Hill, Jonathan R. Emberson, Jonathan Marchini, Pablo Kuri-Morales, Roberto Tapia-Conyer

**Affiliations:** 1 Regeneron Genetics Center, Tarrytown, NY USA; 2 Clinical Trial Service Unit and Epidemiological Studies Unit, Nuffield Department of Population Health, University of Oxford, Oxford, UK; 3 MRC Population Health Research Unit, Nuffield Department of Population Health, University of Oxford, Oxford, UK; 4 Experimental Research Unit from the Faculty of Medicine (UIME), National Autonomous University of Mexico (UNAM), Mexico City, Mexico; 5 Oxford Kidney Unit, Churchill Hospital, Oxford, UK; 6 Instituto Nacional de Medicina Genómica, Tlalpan, Mexico City, Mexico; 7 Centre for Genomics Research, Discovery Sciences, Research and Development Biopharmaceuticals, AstraZeneca, Cambridge, UK; 8 AbbVie, North Chicago, IL USA; 9 Department of Biostatistics, University of Michigan, Ann Arbor, MI USA; 10 Instituto Tecnológico y de Estudios Superiores de Monterrey, Monterrey, Mexico; 11 Faculty of Medicine, National Autonomous University of Mexico, Mexico City, Mexico; 12 Instituto Nacional de Ciencias Médicas y de la Nutrición, Salvador Zubirán Hospital, Mexico City, Mexico; 13 Research Division, Instituto Nacional de Geriatría, Mexico City, Mexico

**Keywords:** Genetic variation, DNA sequencing, Evolutionary genetics, Genetic association study

Correction to: *Nature* 10.1038/s41586-023-06595-3 Published online 11 October 2023

In the version of the article initially published, the surname of Peter Dornbos appeared incorrectly (as Dombos) and has now been corrected in the HTML and PDF versions of the article.

