# Supplementary material for: Genotyping, sequencing and analysis of 140,000 adults from Mexico City
Source: Nature. 2023 Oct 11;622(7984):784–93. doi: 10.1038/s41586-023-06595-3 (PMC10600010; doi:10.1038/s41586-023-06595-3)
Supplement: Supplementary file 1 — Supplementary Figs. 1–28. [file 41586_2023_6595_MOESM1_ESM.pdf]

nature portfolio

https://doi.org/10.1038/s41586-023-06595-3

Supplementary information

Genotyping, sequencing and analysis of 140,000 adults from Mexico City

In the format provided by the authors and unedited

Genotyping, sequencing and analysis of 140,000 adults from Mexico City

SUPPLEMENTARY INFORMATION

Andrey Ziyatdinov[1]*, Jason Torres[2,3]*†, Jesús Alegre-Díaz[4]*, Joshua Backman[1], Joelle Mbatchou[1], Michael Turner[2,5], Sheila M. Gaynor[1], Tyler Joseph[1], Yuxin Zou[1], Daren Liu[1], Rachel Wade[2,3], Jeffrey Staples[1], Razvan Panea[1], Alex Popov[1], Xiaodong Bai[1], Suganthi Balasubramanian[1], Lukas Habegger[1], Rouel Lanche[1], Alex Lopez[1], Evan Maxwell[1], Marcus Jones[1], Humberto García-Ortiz[6], Raul Ramirez-Reyes[4], Rogelio Santacruz-Benítez[4], Abhishek Nag[7], Katherine R. Smith[7], Amy Damask[1], Nan Lin[1], Charles Paulding[1], Mark Reppell[8], Sebastian Zöllner[9], Eric Jorgenson[1], William Salerno[1], Slavé Petrovski[7], John Overton[1], Jeffrey Reid[1], Timothy Thornton[1], Goncalo Abecasis[1], Jaime Berumen[4], Lorena Orozco-Orozco[6], Rory Collins[2], Regeneron Genetics Center, Mexico City Prospective Study, Aris Baras[1], Michael R Hill[2,3], Jonathan R Emberson[2,3], Jonathan Marchini[1], Pablo Kuri-Morales[10,11], Roberto Tapia-Conyer[11]

[1] Regeneron Genetics Center, Tarrytown, NY, USA.

[2] Clinical Trial Service Unit & Epidemiological Studies Unit, Nuffield Department of Population Health, University of Oxford, Oxford, UK

[3] MRC Population Health Research Unit, Nuffield Department of Population Health, University of Oxford, Oxford, UK

[4] Experimental Research Unit from the Faculty of Medicine (UIME), National Autonomous University of Mexico (UNAM)

[5] Oxford Kidney Unit, Churchill Hospital, Oxford, UK

[6] Instituto Nacional de Medicina Genómica, Tlalpan, Mexico City, Mexico

[7] Centre for Genomics Research, Discovery Sciences, Research and Development Biopharmaceuticals, AstraZeneca, Cambridge, UK

[8] AbbVie Inc, North Chicago, Illinois

[9] Department of Biostatistics, University of Michigan, Ann Arbor, USA.

[10] Instituto Tecnológico y de Estudios Superiores de Monterrey, Mexico

[11] Faculty of Medicine, National Autonomous University of Mexico, Mexico City, Mexico

Table of Contents

Supplementary Figure 1: Comparison of hap-IBD and KING pairwise kinship estimates from IBD segments. ... 3
Supplementary Figure 2: Histogram of size of connected components in the 3rd degree relatedness graph. .. 4
Supplementary Figure 3: Summary of first-degree family networks. 5
Supplementary Figure 4: Selected PC scatterplots from a PCA of 500 MCPS samples. 6
Supplementary Figure 5: Selected PC scatterplots from a PCA of 58,051 unrelated MCPS samples................. 7
Supplementary Figure 6: PC SNP loadings from a PCA of 58,051 unrelated MCPS samples, using a LD r^2 threshold of 0.2 for SNP clumping. 8
Supplementary Figure 7: PC SNP loadings from a PCA of 58,051 unrelated MCPS samples, using a LD r^2 threshold of 0.01 for SNP clumping. 9
Supplementary Figure 8: PC SNP loadings from a PCA of 58,051 unrelated MCPS samples , using a LD r^2 threshold of 0.005 for SNP clumping. 10
Supplementary Figure 9 : Low-dimension visualization of IBD sharing.. 11
Supplementary Figure 10 : Low-dimension visualization of haplotype sharing... 12
Supplementary Figure 11: Karyograms showing genome segments from local ancestry inference................ 13
Supplementary Figure 12 : Five Indigenous Mexican ancestry components are compared to overall Indigenous Mexican global ancestry proportions. 14
Supplementary Figure 13 : Correlation in ancestry between spouses. 15
Supplementary Figure 14 : Genome-wide scan for deviation of local ancestry proportions from the global (genome-wide) ancestry proportion. 16
Supplementary Figure 15 : Chromosome 6 distribution of local ancestry proportions. 17
Supplementary Figure 16 : ROH segments by ancestry. 18
Supplementary Figure 17 : Loss of function variants by ROH. 19
Supplementary Figure 18 : Phasing accuracy of WGS dataset. 20
Supplementary Figure 19 : Phasing accuracy of WGS dataset stratifies by ancestry....................................... 21
Supplementary Figure 20 : Imputation accuracy using the MCPS10k and TOPMed imputation panels applied to 1000 Genomes samples. 22
Supplementary Figure 21 : Imputation accuracy using the MCPS10k and TOPMed imputation panels applied to 1000 Genomes samples. 23
Supplementary Figure 22 : Comparison of ancestry proportions between MCPS and MXL samples. 24
Supplementary Figure 23 : Allele frequency comparison between MCPS WGS and gnomAD.......................... 25
Supplementary Figure 24 : Allele frequency comparison between MCPS WGS and gnomAD LAI estimates. .. 26
Supplementary Figure 25: Schematic depicting the IBD graph construction at a single locus.......................... 27
Supplementary Figure 26: Comparison of methods for allele frequency estimation. 28
Supplementary Figure 27: Comparison of methods for allele frequency estimation. 29
Supplementary Figure 28 : IBD segment coverage from 10K MCPS samples before (left) and after (right) filtering. 30

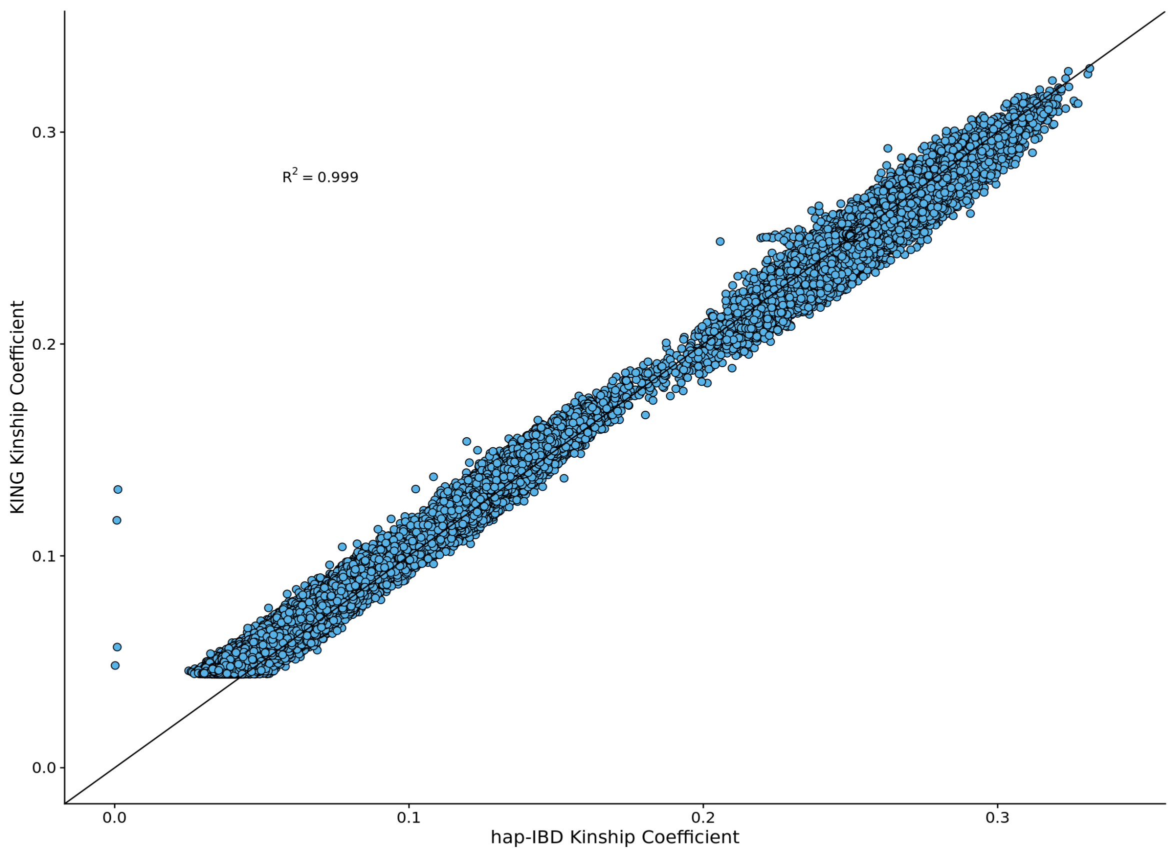

Supplementary Figure 1: Comparison of hap-IBD and KING pairwise kinship estimates from IBD segments. The estimates are highly correlated; four outliers are observed where coefficients were estimated to be zero by hap-IBD, likely due to phasing or genotyping error introducing breaks in detecting IBD segments.

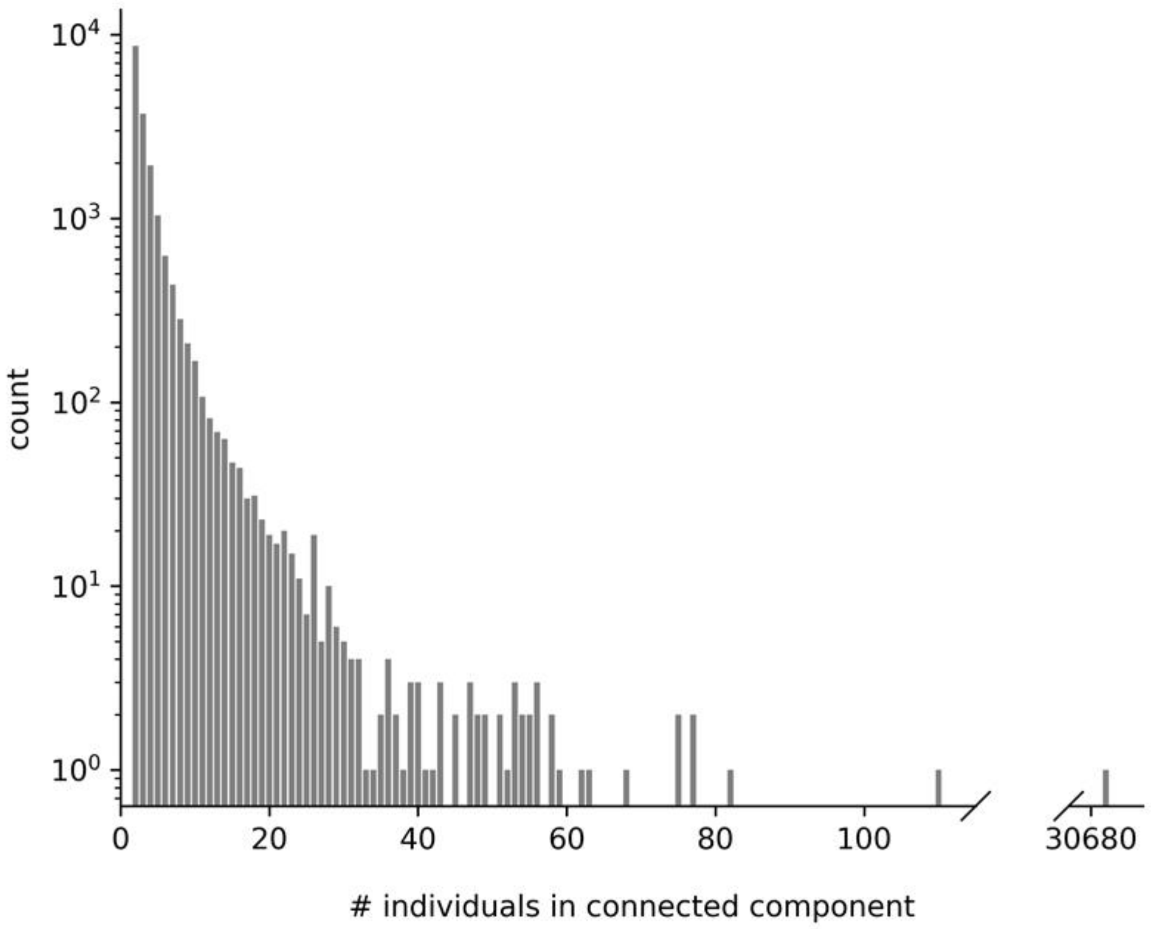

Supplementary Figure 2: Histogram of size of connected components in the 3rd degree relatedness graph. The largest connected component has size 30,680 and is shown on the histogram using a split axis.

A

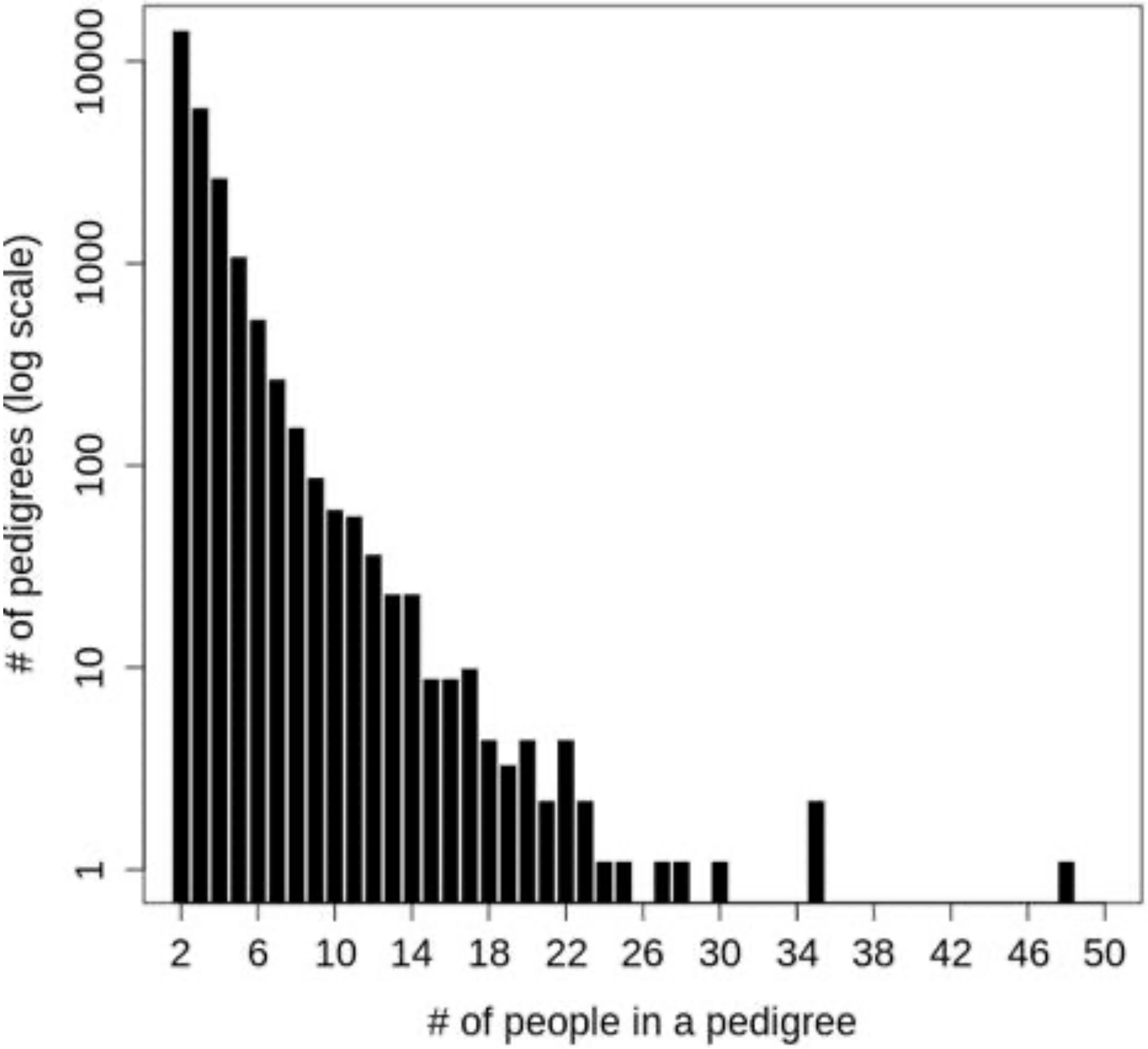

B

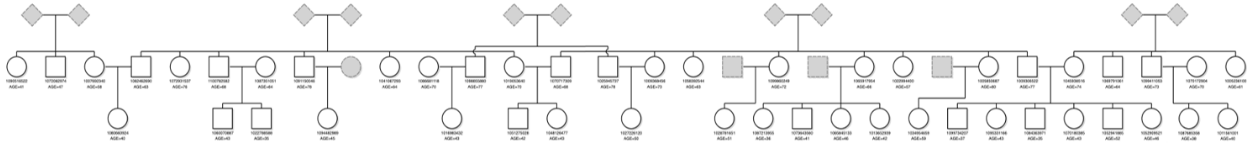

Supplementary Figure 3: Summary of first-degree family networks. (A) distribution of family network sizes and (B) largest first-degree family network of 48 individuals.

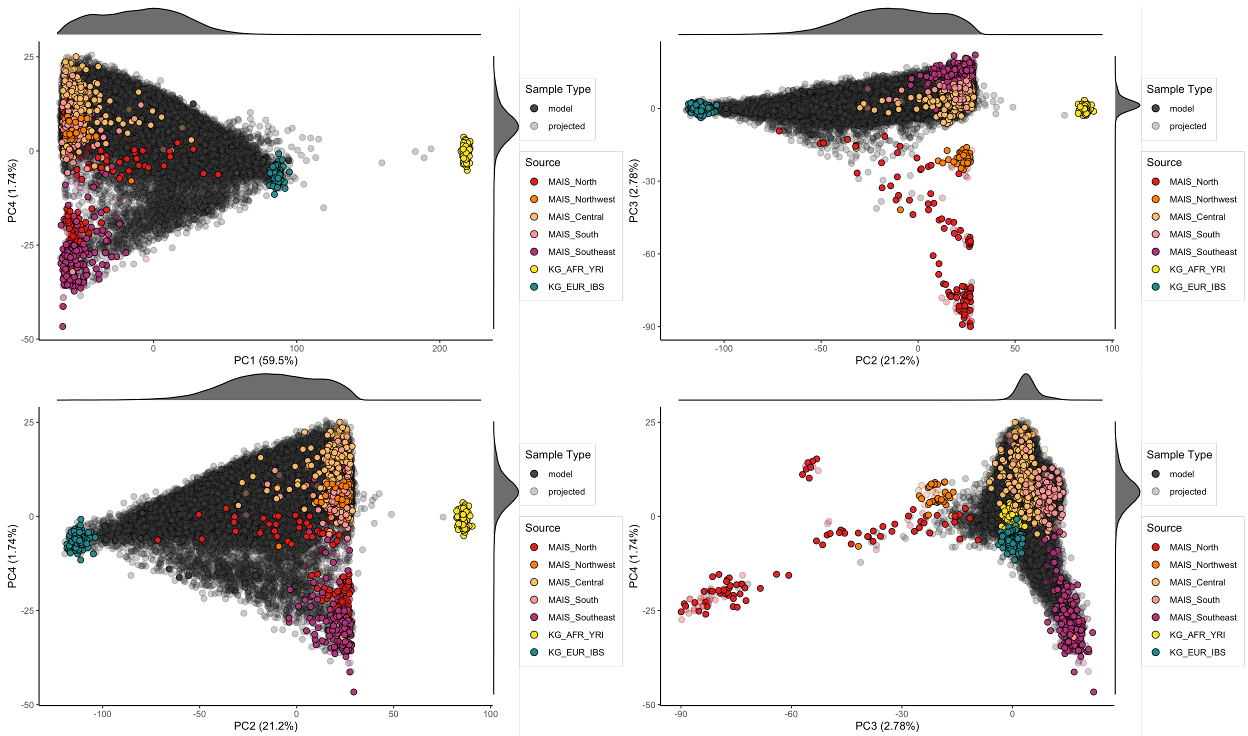

Supplementary Figure 4: Selected PC scatterplots from a PCA of 500 MCPS samples. Remaining MCPS samples and reference samples from the 1000 Genomes Project (Yoruba – YRI; Iberian – IBS) and MAIS (Indigenous samples from Mexico) projected onto the PC axes. Figure 2 shows PC1 vs PC2 and PC1 vs PC3.

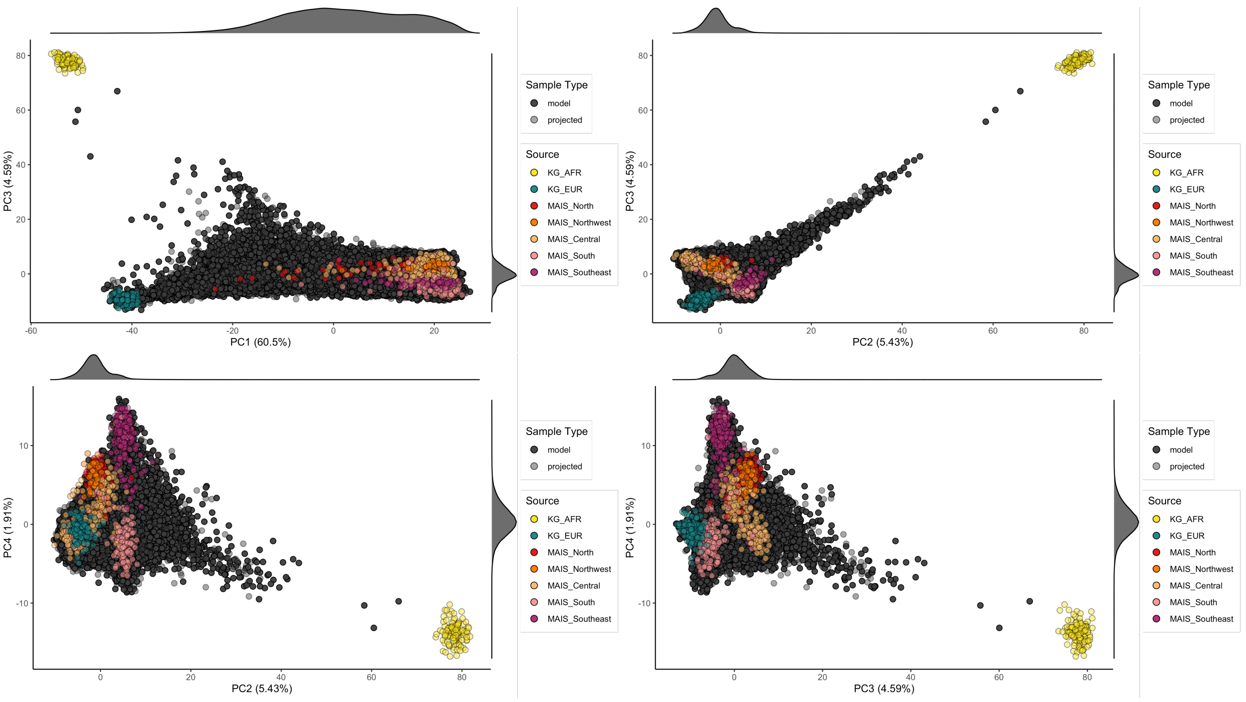

Supplementary Figure 5: Selected PC scatterplots from a PCA of 58,051 unrelated MCPS samples. Remaining MCPS samples and reference samples from the 1000 Genomes Project (Yoruba – YRI; Iberian – IBS) and MAIS (Indigenous samples from Mexico) projected onto the PC axes. Figure 2 shows PC1 vs PC2 and PC1 vs PC4.

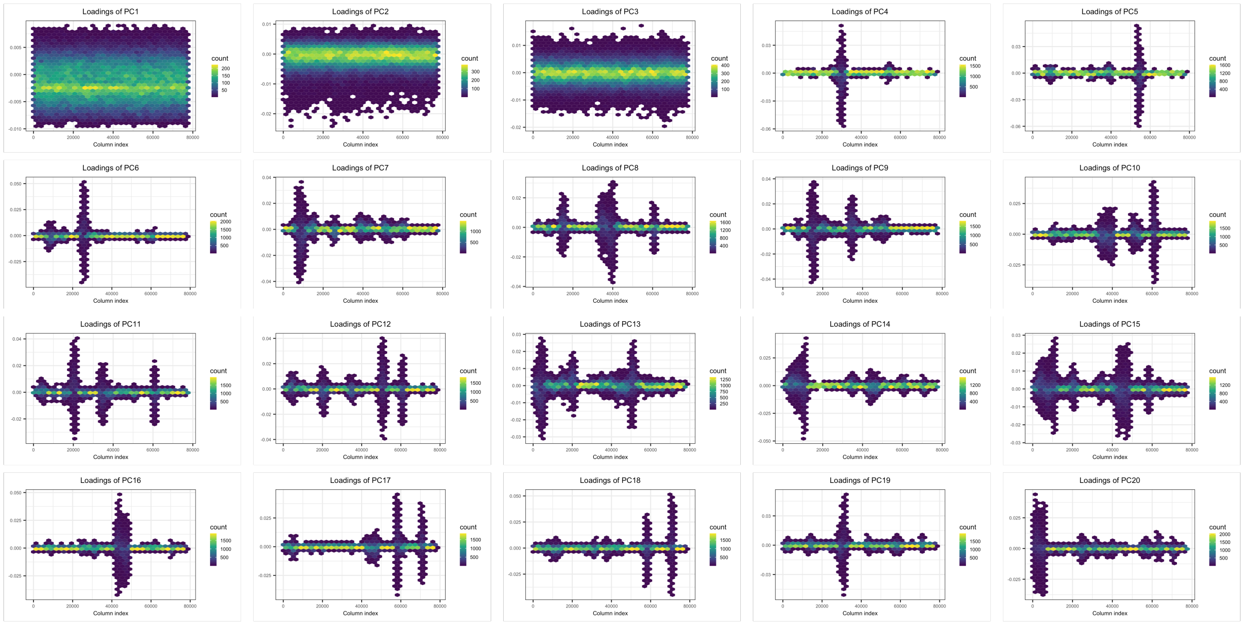

Supplementary Figure 6: PC SNP loadings from a PCA of 58,051 unrelated MCPS samples, using a LD r^2 threshold of 0.2 for SNP clumping.

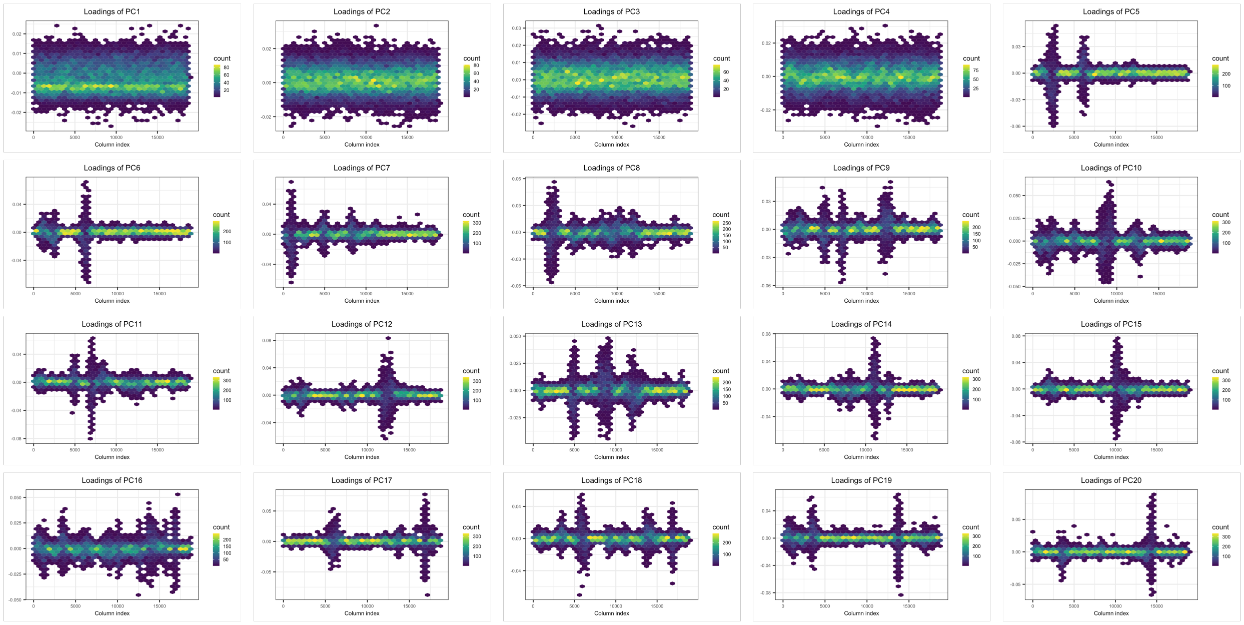

Supplementary Figure 7: PC SNP loadings from a PCA of 58,051 unrelated MCPS samples, using a LD r^2 threshold of 0.01 for SNP clumping.

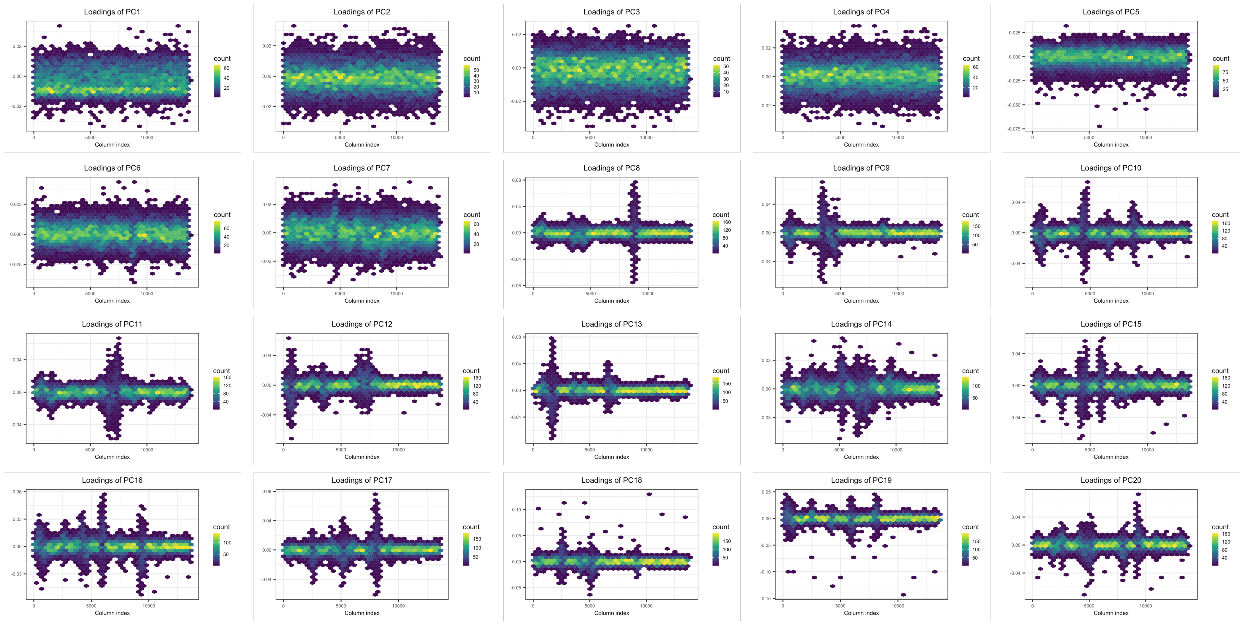

Supplementary Figure 8: PC SNP loadings from a PCA of 58,051 unrelated MCPS samples , using a LD r^2 threshold of 0.005 for SNP clumping.

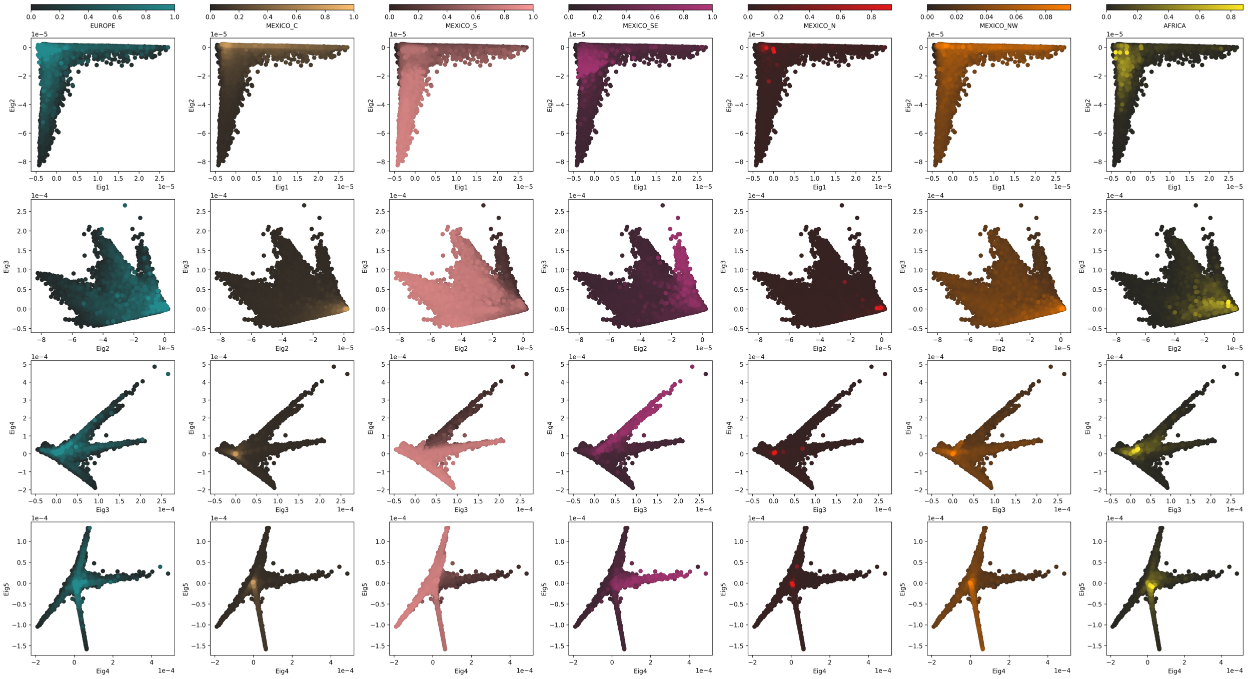

Supplementary Figure 9 : Low-dimension visualization of IBD sharing. Rows are different pairs of eigenvector scores. Columns show points coloured by seven different ancestry proportions obtained from the results of local ancestry inference with RFMix.

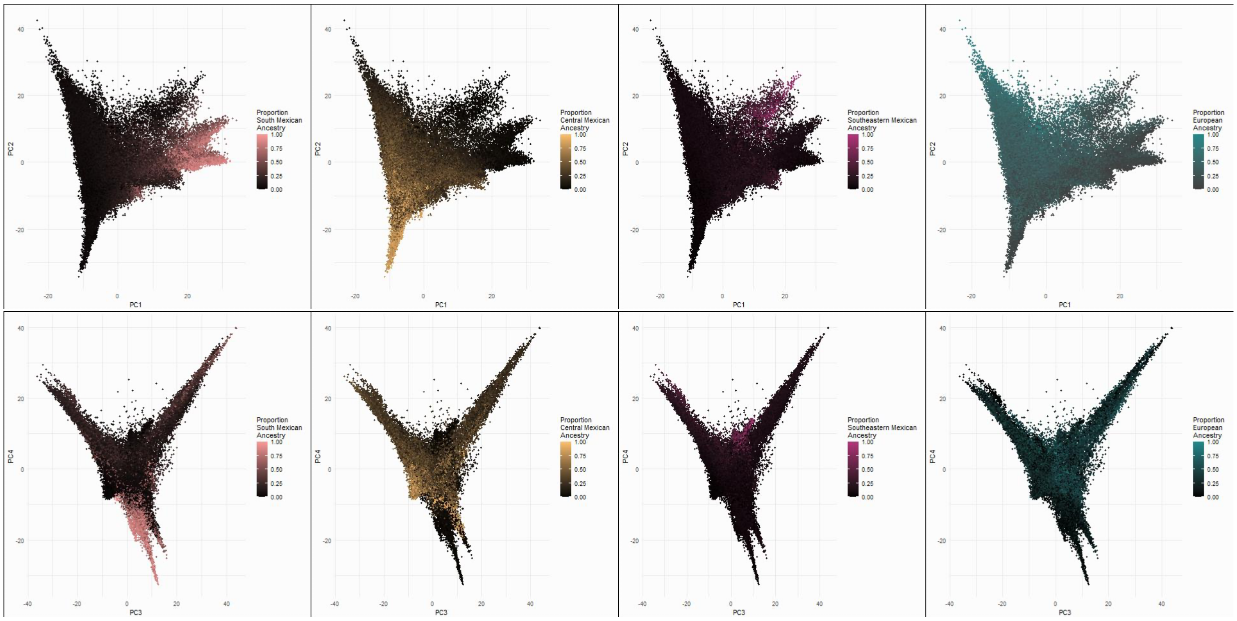

Supplementary Figure 10 : Low-dimension visualization of haplotype sharing. PC1 vs PC2 (Row 1) and PC3 vs PC4 (Row 2) . Columns are coloured using different ancestry proportions from the RFMix analysis.

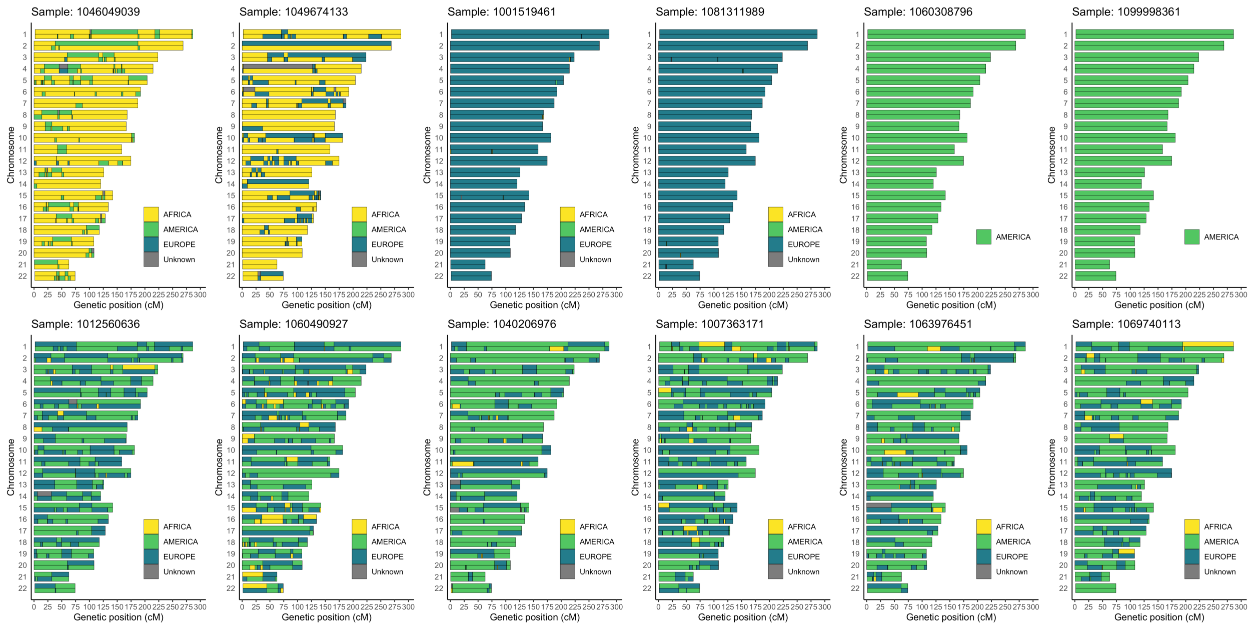

Supplementary Figure 11: Karyograms showing genome segments from local ancestry inference. LAI of a three-way admixture model was performed using a random-forest based method implemented in *RFMix*. Haplotypes with inferred genome segments derived from ancestral African (yellow), European (blue), and Indigenous American (green) populations are shown for 12 MCPS samples. The top row shows samples with high proportions of African, European and Indigenous American ancestry. The bottom row shows more typical admixed samples.

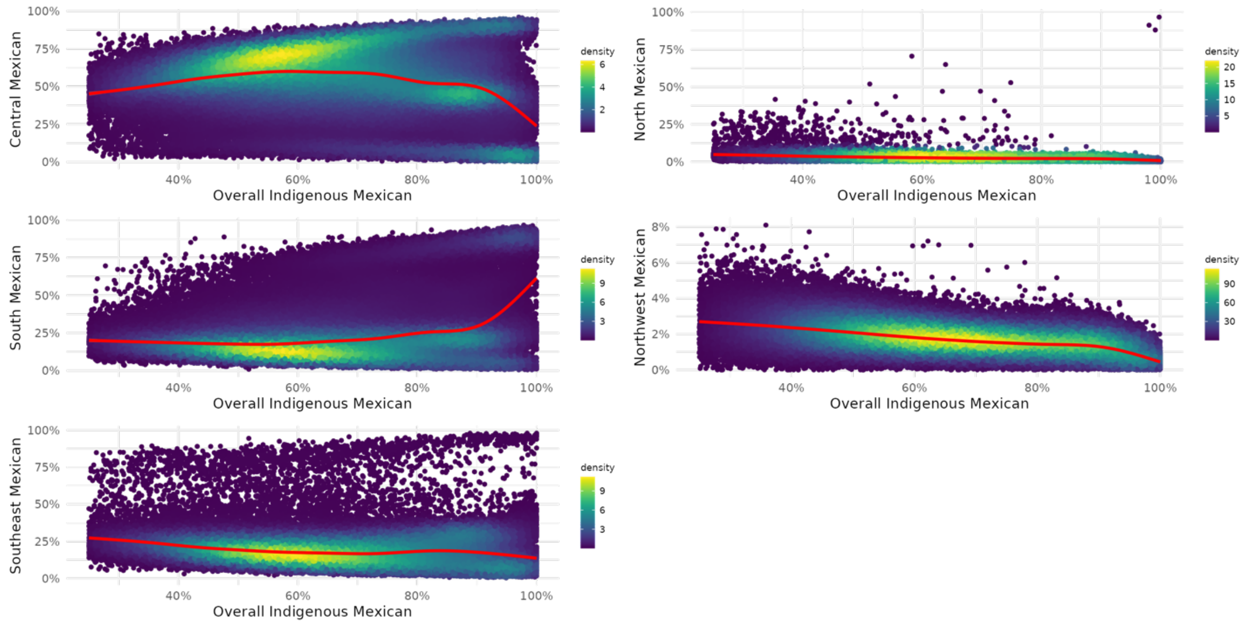

Supplementary Figure 12 : Five Indigenous Mexican ancestry components are compared to overall Indigenous Mexican global ancestry proportions. Each scatterplot shows individual-level ancestry proportions for 136,935 out of 138,511 MCPS samples with overall Indigenous Mexican proportion > 25%. The point color underlines the estimated 2D density, and the red line is cubic-spline regression fit.

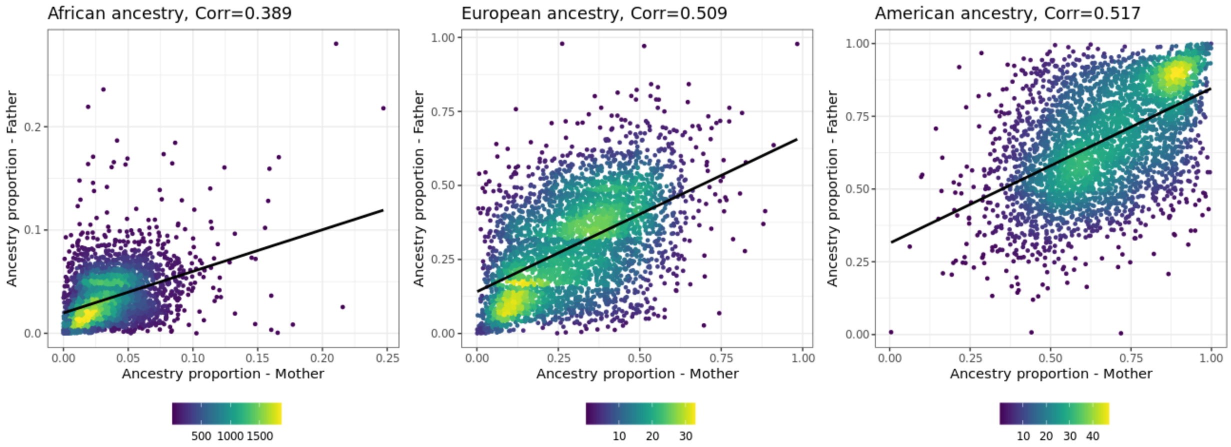

Supplementary Figure 13 : Correlation in ancestry between spouses. The plots show the proportion of ancestry of the mother and father of 3,595 parent couples inferred from the genetic relatedness analysis. Ancestry from the 3-way RFMix analysis was used to determine the proportions of African (left), European (middle) and Indigenous American (right) ancestry.

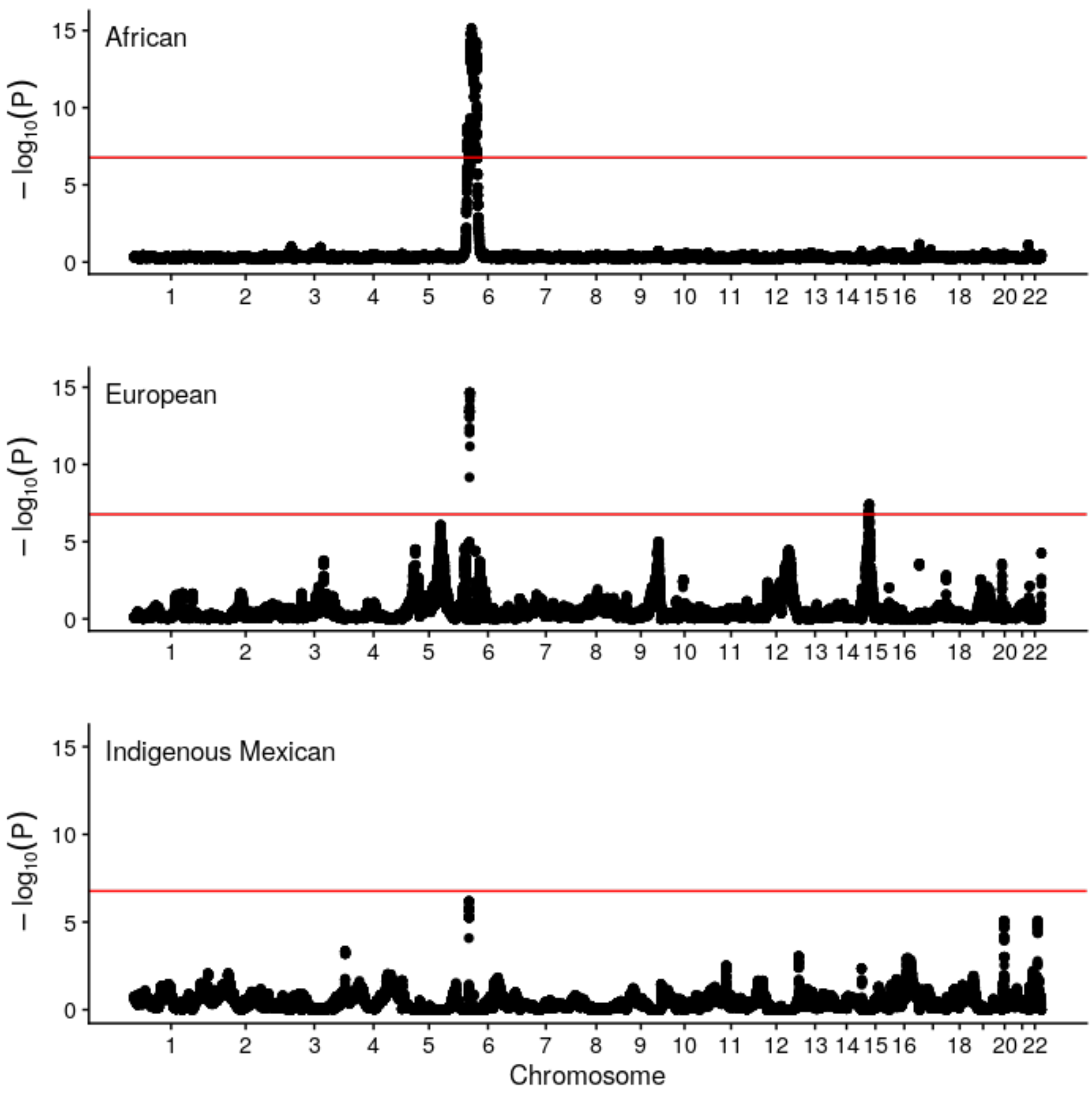

Supplementary Figure 14 : Genome-wide scan for deviation of local ancestry proportions from the global (genome-wide) ancestry proportion. The local ancestry dosages are tested for difference from the global ancestry proportion, separately for each of African, European, and Indigenous Mexican ancestries. Two genome-wide significant loci on Chromosomes 6 and 15 replicate previously reported selection signals in the MHC region (the immune system) and the *SLC24A5* gene (skin pigmentation).

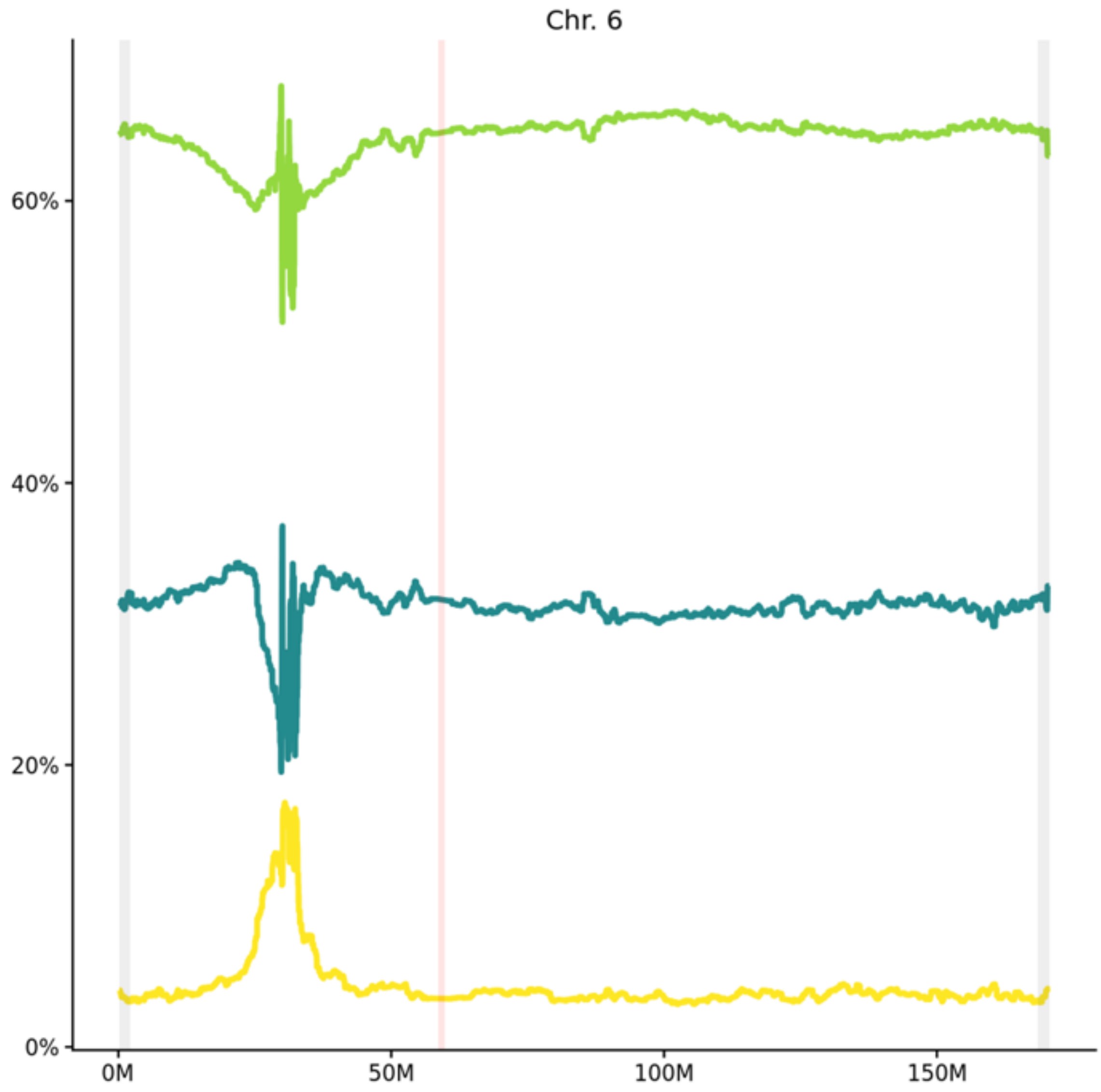

Supplementary Figure 15 : Chromosome 6 distribution of local ancestry proportions. The ancestry dosages inferred by RFMix are averaged across 78,833 unrelated MCPS samples and plotted along chromosome 6. The two gray bars denote terminal 2Mbp-length regions (of analyzed sites) at the beginning and end of the chromosome, while the red bar denotes the centromere region. The peak of 17.3% African ancestry encompasses a region on chromosome 6 between 2.82 and 3.29 Mb. Under assumption of binomial sampling and normal approximation for sample mean, we obtain a p-value 2.9e-14 for African ancestry to exceed 17%. These results replicate findings previously reported in Guan et al 2014

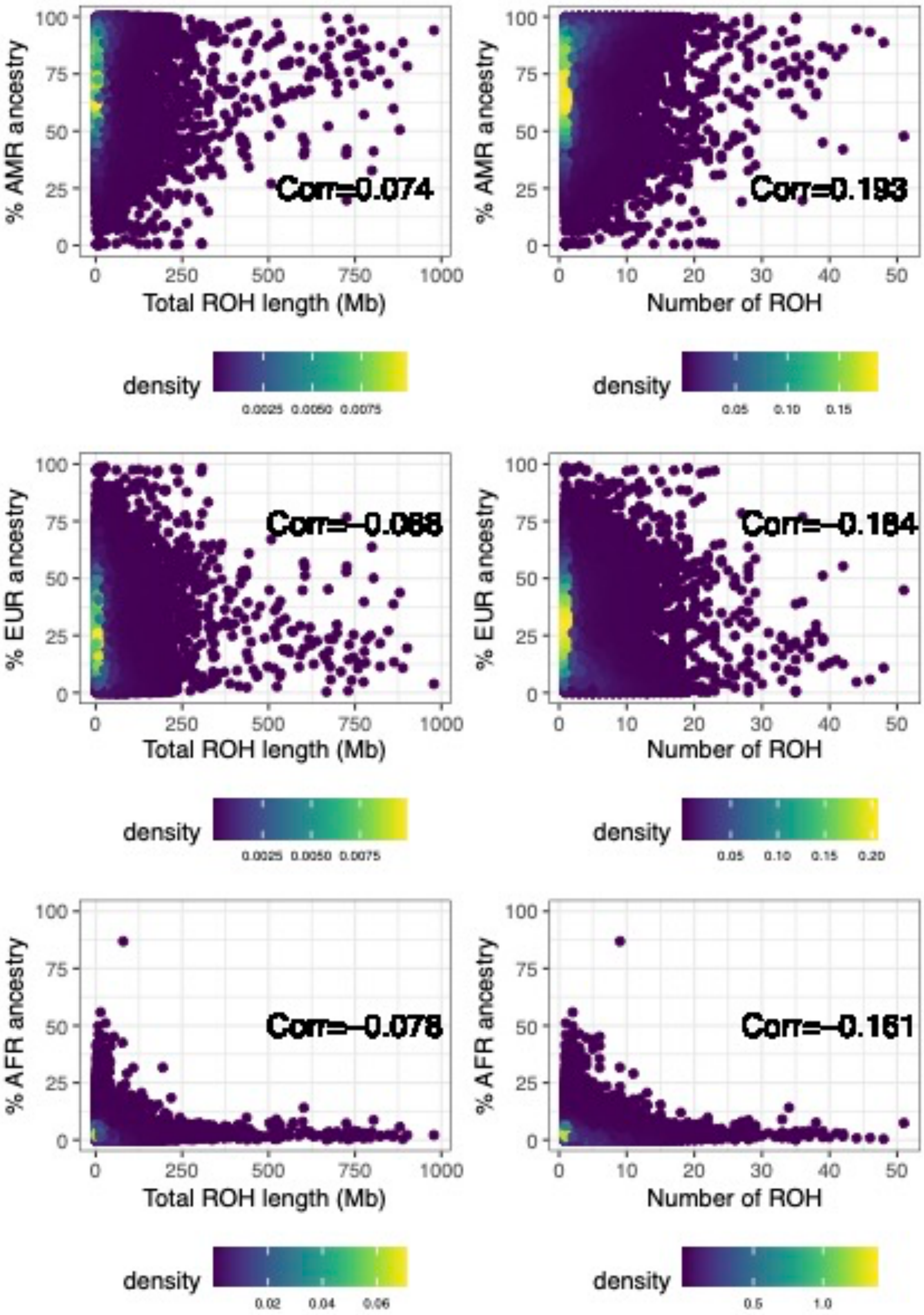

Supplementary Figure 16 : ROH segments by ancestry. The fraction of ancestry attributed as Indigenous Mexican, African, and European for each individual is given by total ROH length and number of ROH segments.

A

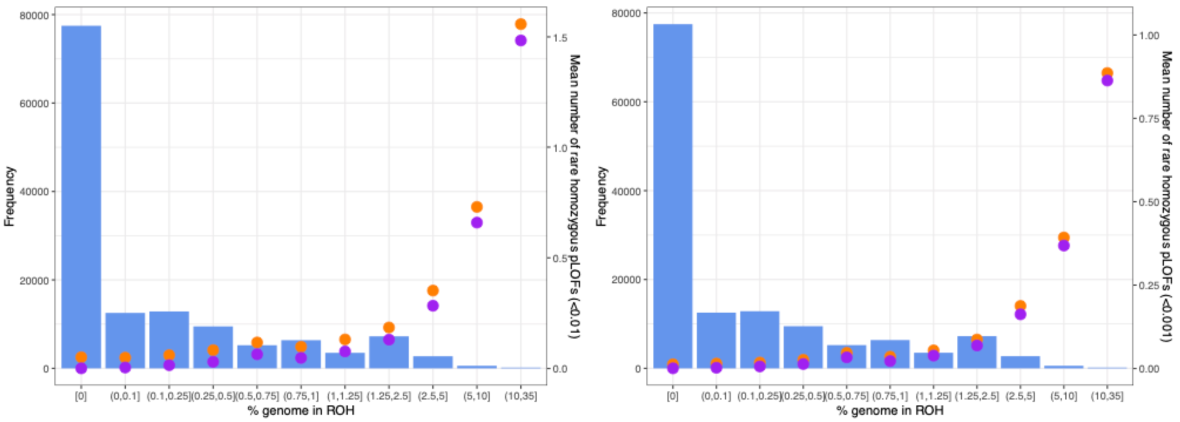

B

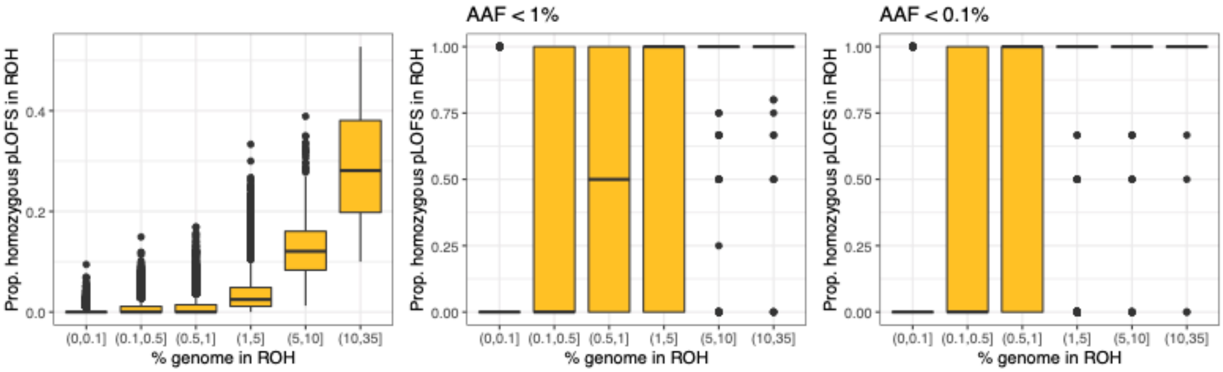

Supplementary Figure 17 : Loss of function variants by ROH. **(A)** The number of individuals and average number of rhLOFs within ROH (purple) and overall (orange) are given by the fraction of the genome in ROH. **(B)** The per-sample proportion of homozygous pLOFs falling within ROH (for n=138,200 samples) are given by fraction of the genome in ROH, for all frequencies, AAF<1%, and AAF<0.1%. Data are presented with the median as the center, the box bounded by the 25th and 75th percentiles, whiskers extending from the box to values within 1.5*IQR (Interquartile Range), and outlying values such as minima/maxima as black points.

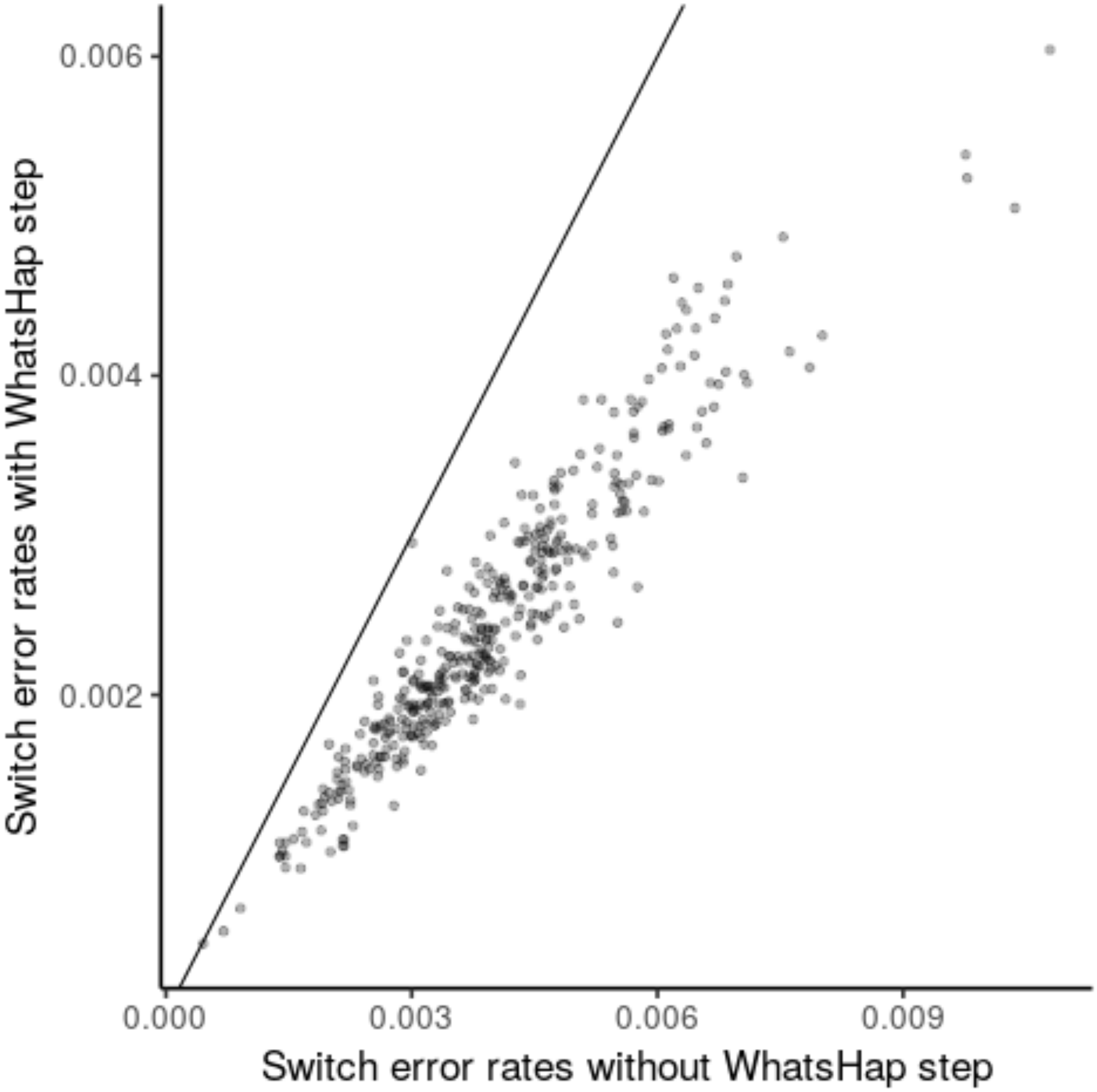

Supplementary Figure 18 : Phasing accuracy of WGS dataset. The phasing switch error for each of 392 individuals phased with (y-axis) and without (x-axis) using the the WhatsHap method to leverage phase information in sequencing reads. The 392 individuals are parents of mother-father-child trios that were phased without including the children.

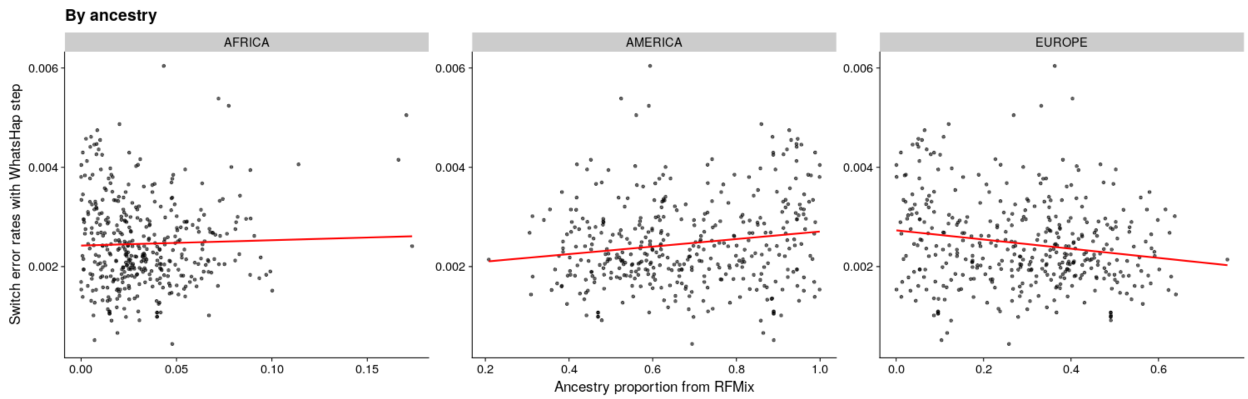

Supplementary Figure 19 : Phasing accuracy of WGS dataset stratifies by ancestry. The phasing switch error (y-axis) for each of 392 individuals plotted against estimated proportion (x-axis) of African ancestry (left), American ancestry (middle) and European ancestry (right). The p-values for the test that the regression line being different from 0 was 0.0016, 0.0004 and 0.53 for proportion of American, European and African ancestry respectively. The 392 individuals are parents of mother-father-child trios that were phased without including the children.

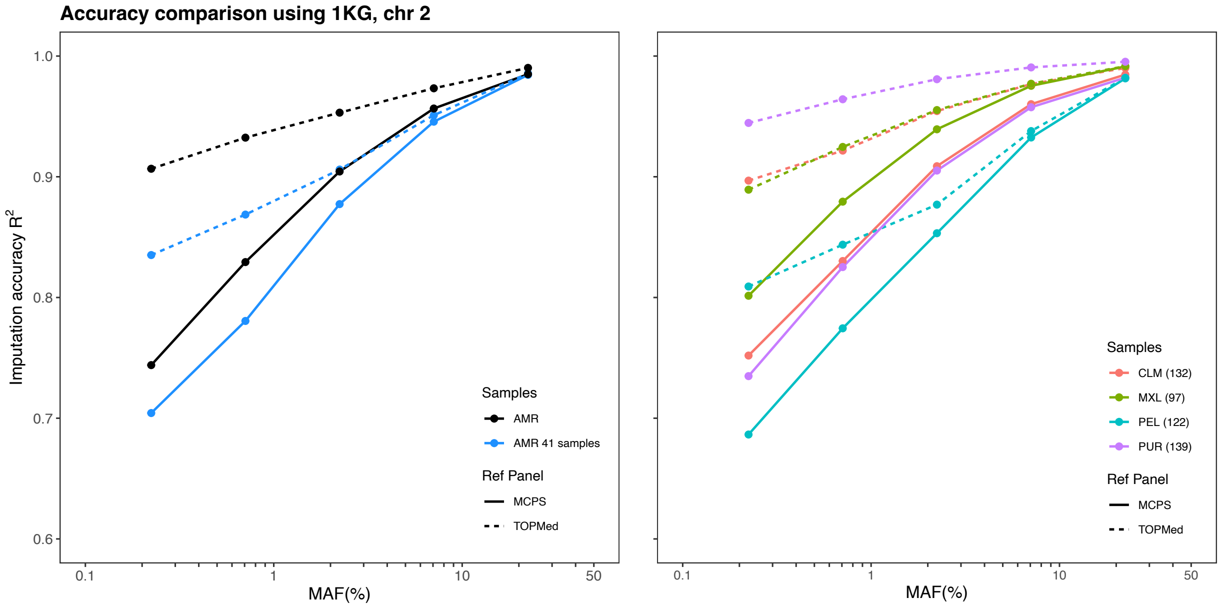

Supplementary Figure 20 : Imputation accuracy using the MCPS10k and TOPMed imputation panels applied to 1000 Genomes samples. Accuracy is measured using the R^2 between the imputed variants and 1,610,207 variants from the 1000 Genomes WGS dataset. Imputation was based on genotypes at SNPs on the Illumina HumanOmniExpressExome-8v1-2_A array. Results are stratified by allele frequency in the 490(x-axis on log10 scale), reference panel (solid = MCPS, dotted = TOPMed) and by populations. In the left hand plot shows results at on all samples with American ancestry (AMR) and on the 41 samples with >90% American ancestry. The right hand plot shows the results stratified by the four groups : Mexican ancestry from Los Angeles (MXL), Peruvian ancestry from Lima (PEL), Colombian ancestry from Medellin (CLM) and Puerto Rican ancestry from Puerto Rico (PUR) .

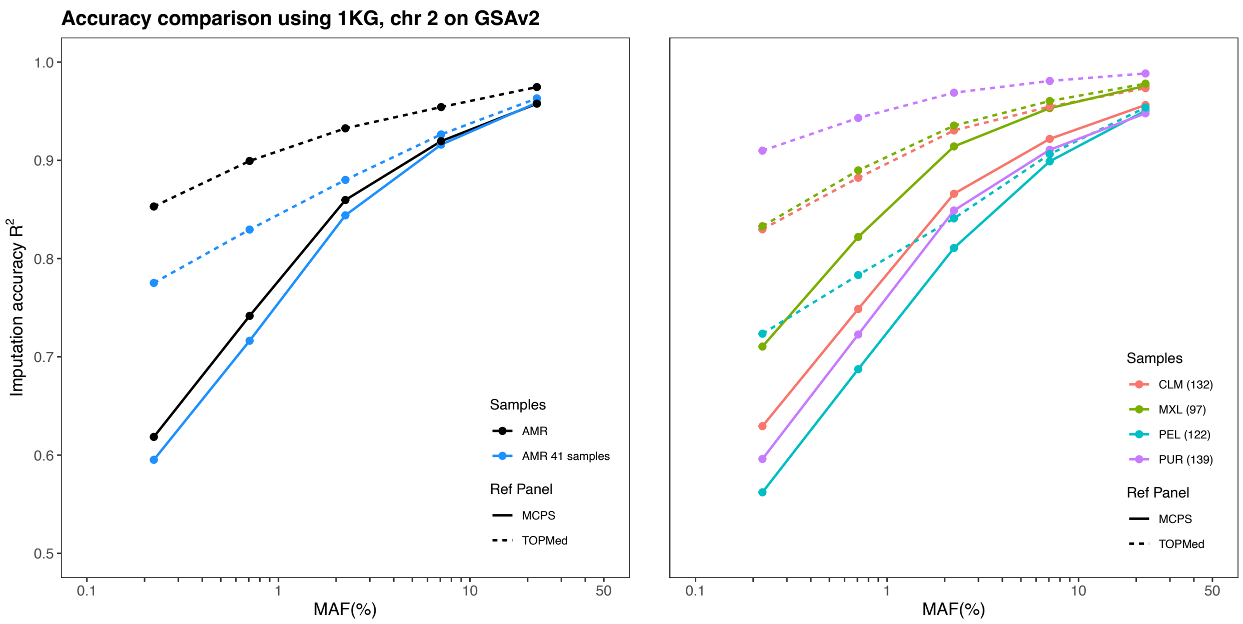

Supplementary Figure 21 : Imputation accuracy using the MCPS10k and TOPMed imputation panels applied to 1000 Genomes samples. Accuracy is measured using the R^2 between the imputed variants and 1,610,207 variants from the 1000 Genomes WGS dataset. Imputation was based on genotypes at SNPs on the Illumina Global Screening Array (GSA v2). Results are stratified by allele frequency in the 490(x-axis on log10 scale), reference panel (solid = MCPS, dotted = TOPMed) and by populations. In the left hand plot shows results at on all samples with American ancestry (AMR) and on the 41 samples with >90% American ancestry. The right hand plot shows the results stratified by the four groups : Mexican ancestry from Los Angeles (MXL), Peruvian ancestry from Lima (PEL), Colombian ancestry from Medellin (CLM) and Puerto Rican ancestry from Puerto Rico (PUR) .

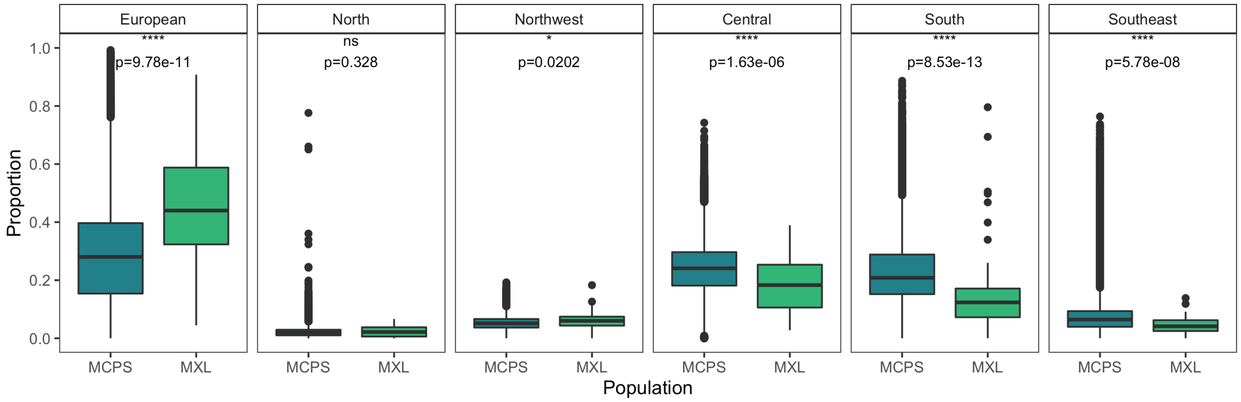

Supplementary Figure 22 : Comparison of ancestry proportions between MCPS and MXL samples. Ancestry proportions correspond to inferred ancestries from the ADMIXTURE analysis that had the highest average proportions among Indigenous Mexican populations from five major geographic regions (as delineated in García-Ortiz et al. 2021.) and among European populations. The sample sizes were n=138,511 participants from MCPS and n=64 Mexican American participants from Los Angeles (MXL) from the 1000 Genomes Project v3. Differences in ancestry proportions between cohorts were assessed with two-sided Mann Whitney U tests. Box plots indicate median and interquartile range (IQR) of ancestry proportions with Tukey-style whiskers corresponding to 1.5 x IQR beyond the box boundaries. Samples with values outside of the whisker boundaries are shown as black circles.

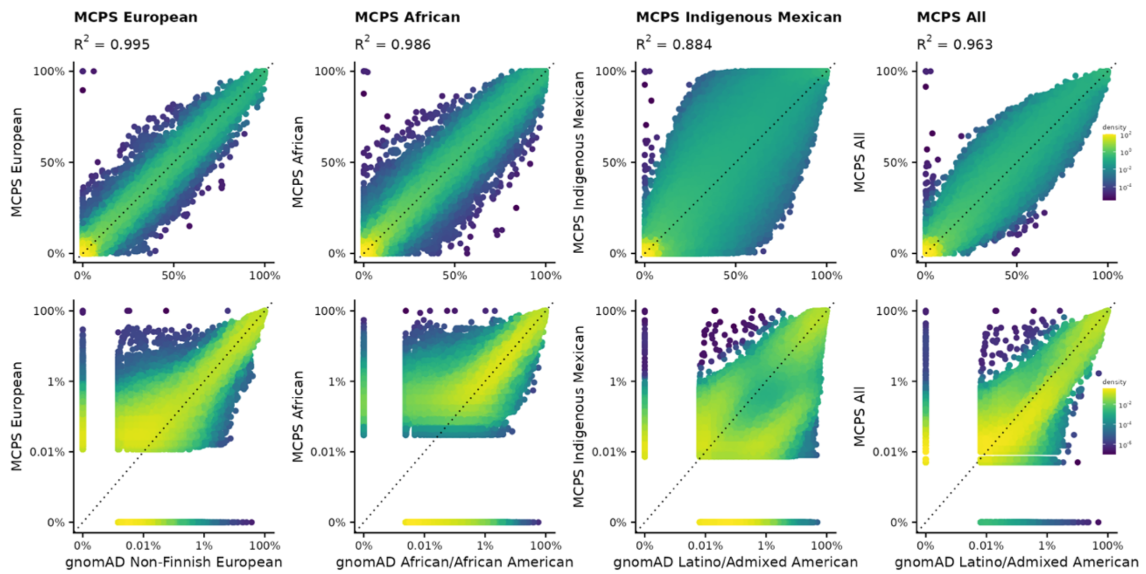

Supplementary Figure 23 : Allele frequency comparison between MCPS WGS and gnomAD. Allele frequencies on linear (top) and log (bottom) scale. The comparisons from left to right are MCPS European vs gnomAD Non-Finnish European, MCPS African vs gnomAD African, MCPS Indigenous Mexican vs gnomAD Latino/Admixed American and overall MCPS vs gnomAD Latino/Admixed American. The number N in each title represents the estimated effective sample size for the MCPS allele frequency estimation.

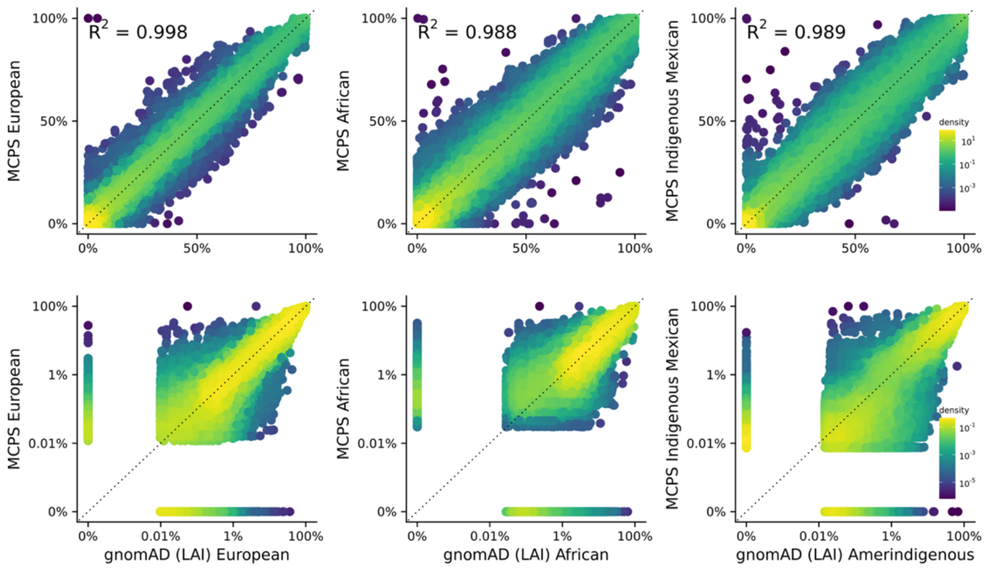

Supplementary Figure 24 : Allele frequency comparison between MCPS WGS and gnomAD LAI estimates. Allele frequencies on linear (top) and log (bottom) scale. The comparisons from left to right are MCPS European vs gnomAD (LAI) European, MCPS African vs gnomAD (LAI) African, MCPS Indigenous Mexican vs gnomAD Amerindigenous. The gnomAD (LAI) refers to an extension to the gnomAD v3 database with local ancestry resolved allele frequency estimates for Latino/Admixed American samples in gnomAD (see URLs). The number of high-quality variants overlapped between MCPS WGS and gnomAD (LAI) is 11,154,612, 10,262,648 and 9,385,266 for European, African and Amerindigenous ancestries, respectively.

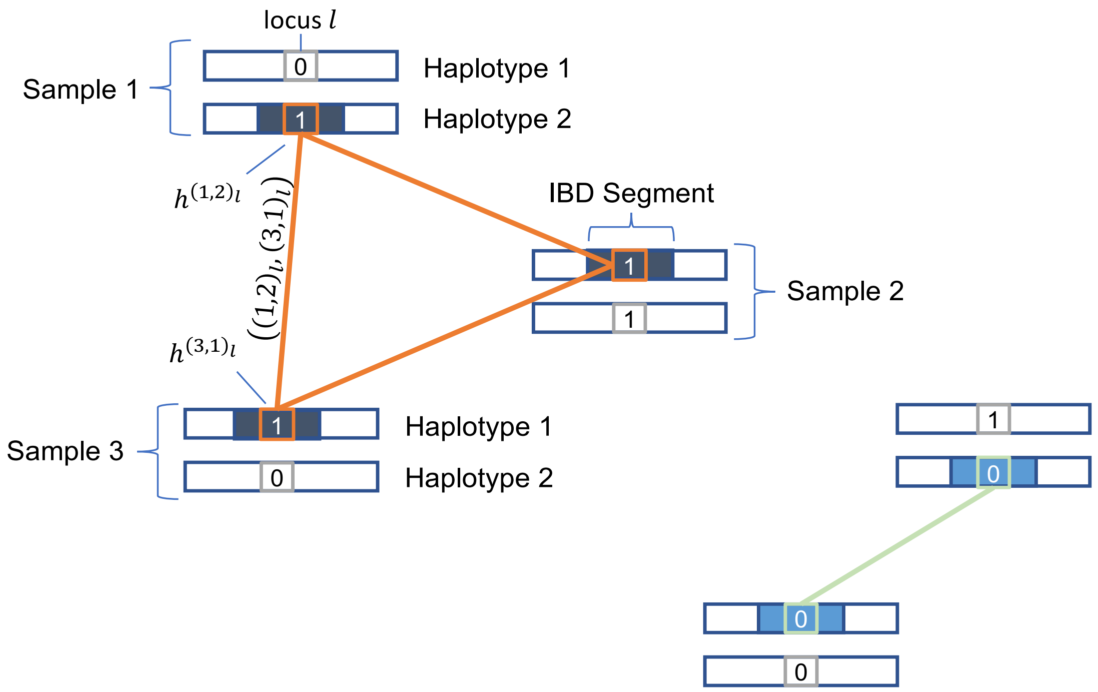

Supplementary Figure 25: Schematic depicting the IBD graph construction at a single locus. Each sample contributes 2 haplotypes (pairs of large rectangles) to the IBD graph. Haplotypes carrying the alternate allele at the locus (small squares) are labeled 1, and haplotypes carrying the reference allele are labeled 0. Two haplotypes are connected by an edge (lines) if they are inherited IBD (solid small rectangles). An example of the IBD graph's mathematical definition is illustrated among one edge of the orange connected component between samples 1, 2, and 3 (see Methods for additional details). The alternate allele count (AC) is computed by counting connected components of haplotypes carrying the alternate allele. The allele number (AN) is computed by counting the total number of connected components. In the figure, the IBD corrected AC is 3 while the uncorrected AC is 5; the IBD corrected AN is 7 while the uncorrected AN is 10.

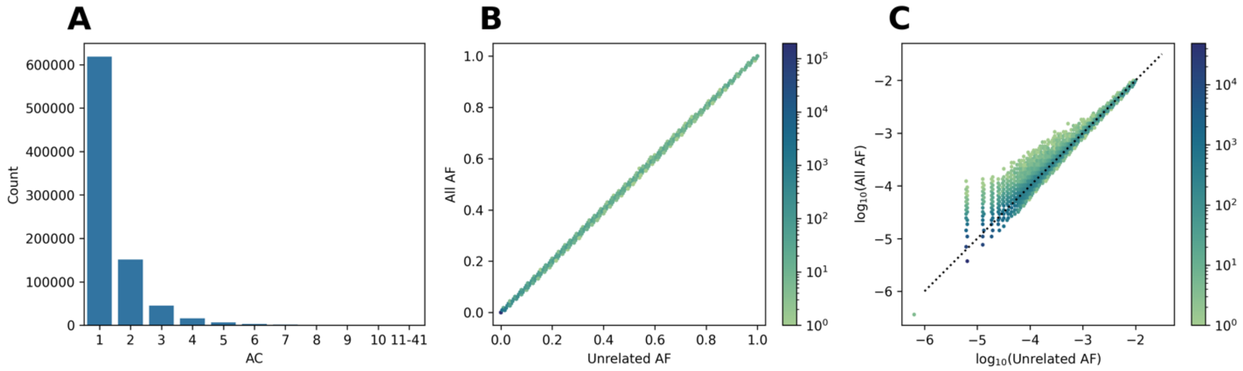

Supplementary Figure 26: Comparison of methods for allele frequency estimation. **(A)** Histogram of the alternate allele count of variants missing from the unrelated subset computed from the combined WES and array dataset for all chromosomes. **(B)** Hexbin plot of allele frequencies computed using the unrelated subset (*x*-axis) and all samples (*y*-axis) and for chromosome 22. **(C)** Hexbin plot of $\log_{10}$ allele frequencies of rare variants (AAF<0.01) on chromosome 22.

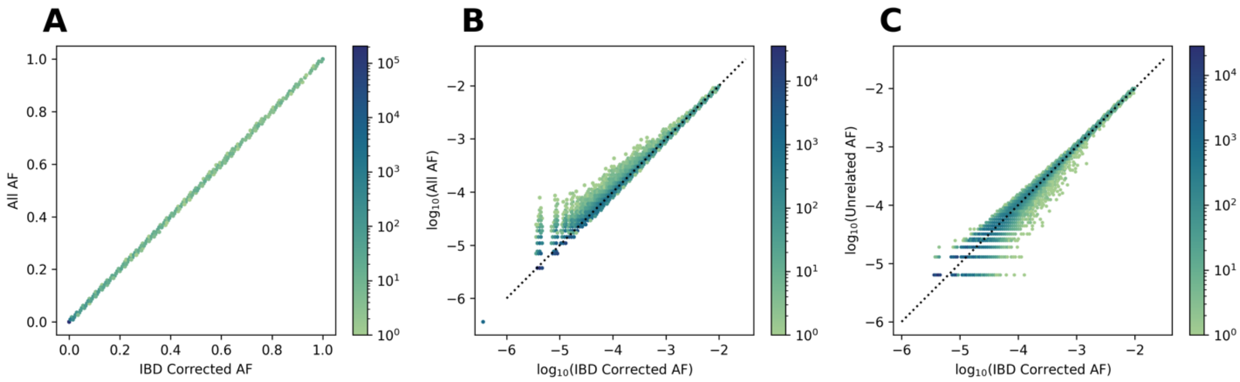

Supplementary Figure 27: Comparison of methods for allele frequency estimation. **(A)** Alternate allele frequencies computed for chromosome 22 correcting for IBD (*x*-axis) and computed from all samples (*y*-axis). **(B)** Log_{10} alternate allele frequencies for rare variants (AAF<0.01) computed for chromosome 22 correcting for IBD (*x*-axis) and computed from all samples (*y*-axis). **(C)** Log_{10} alternate allele frequencies for rare variants (AAF<0.01) computed for chromosome 22 correcting for IBD (*x*-axis) and computed from unrelated samples (*y*-axis).

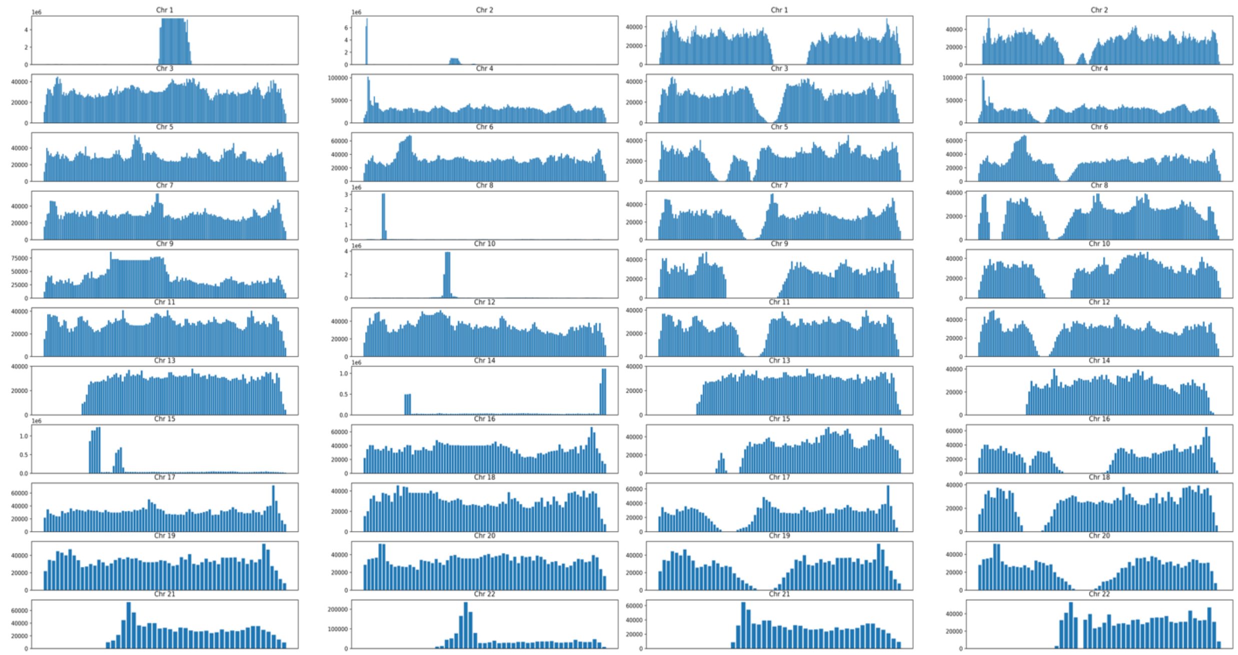

Supplementary Figure 28 : IBD segment coverage from 10K MCPS samples before (left) and after (right) filtering. IBD segments were filtered out if they intersected a 1Mb bin with either i) fourfold more than the median IBD coverage along a chromosome, or ii) fourfold fewer than the median number of SNP array markers.
