# Supplementary material for: Genotyping, sequencing and analysis of 140,000 adults from Mexico City
Source: Nature. 2023 Oct 11;622(7984):784–93. doi: 10.1038/s41586-023-06595-3 (PMC10600010; doi:10.1038/s41586-023-06595-3)
Supplement: Supplementary file 2 — Reporting Summary [file 41586_2023_6595_MOESM2_ESM.pdf]

nature portfolio

Corresponding author(s): Jonathan Marchini

Last updated by author(s): Aug 11, 2023

Reporting Summary

Nature Portfolio wishes to improve the reproducibility of the work that we publish. This form provides structure for consistency and transparency in reporting. For further information on Nature Portfolio policies, see our Editorial Policies and the Editorial Policy Checklist.

Statistics

For all statistical analyses, confirm that the following items are present in the figure legend, table legend, main text, or Methods section.

n/a	Confirmed	
☐	☒	The exact sample size (*n*) for each experimental group/condition, given as a discrete number and unit of measurement
☐	☒	A statement on whether measurements were taken from distinct samples or whether the same sample was measured repeatedly
☐	☒	The statistical test(s) used AND whether they are one- or two-sided<br>*Only common tests should be described solely by name; describe more complex techniques in the Methods section.*
☐	☒	A description of all covariates tested
☐	☒	A description of any assumptions or corrections, such as tests of normality and adjustment for multiple comparisons
☐	☒	A full description of the statistical parameters including central tendency (e.g. means) or other basic estimates (e.g. regression coefficient) AND variation (e.g. standard deviation) or associated estimates of uncertainty (e.g. confidence intervals)
☐	☒	For null hypothesis testing, the test statistic (e.g. *F*, *t*, *r*) with confidence intervals, effect sizes, degrees of freedom and *P* value noted<br>*Give P values as exact values whenever suitable.*
☒	☐	For Bayesian analysis, information on the choice of priors and Markov chain Monte Carlo settings
☐	☒	For hierarchical and complex designs, identification of the appropriate level for tests and full reporting of outcomes
☐	☒	Estimates of effect sizes (e.g. Cohen's *d*, Pearson's *r*), indicating how they were calculated

Our web collection on statistics for biologists contains articles on many of the points above.

Software and code

Policy information about availability of computer code

Data collection

No software was used for data collection.

Data analysis

SHAPEIT (v4.1.2, v4.1.3, v4.2.2) was used for haplotype estimation.
QCTOOL (v2) was used for calculating imputation info scores.
MakeScaffold was used for building a haplotype scaffold.
Hap-IBD (v1.0) was used to identify IBD segments
IBDkin (v2.8.7.8) was used for kinship estimation.
PLINK (v1.9 and v2) were used for data processing and quality control.
RFmix (v2) was used for local ancestry estimation
TeraSTRUCTURE was used for ancestry analysis.
IMPUTE5 (v1.1.5) was used for imputation experiments.
PRIMUS (v1.9.0) was used for family estimation.
GRAPHVIZ (v6.0.2) was used for relatedness visualization.
ADMIXTURE (v1.3.0) was used for ancestry analysis.
KING (v2.2.8) was used for kinship estimation.
DeepVariant (v0.10.0) was used for variant calling.
The R packages bigsnpr and bigstatsr were used for PCA.
REGENIE (v3.2.8) was used for association testing.
LDpred (v1.0.11) was used for PRS estimation.
EAGLE (v2.4.1) was used in the TOPMed server for phasing.
MINIMAC v4 was used in the TOPMed server for imputation.

GLnexus (v1.3.1) was used for joint genotype calling.
WhatsHap (v1.4) was used for read-base phasing.
PRIMUS was used for relatedness inference.

Code developed for the analysis of this paper is available from two separate Github repositories at
https://github.com/mcps-analysts/mcps-genetic-cohort-profile
https://github.com/rgcgithub/mcps_ibd_freq_calc

For manuscripts utilizing custom algorithms or software that are central to the research but not yet described in published literature, software must be made available to editors and reviewers. We strongly encourage code deposition in a community repository (e.g. GitHub). See the Nature Portfolio guidelines for submitting code & software for further information.

Data

Policy information about availability of data

All manuscripts must include a data availability statement. This statement should provide the following information, where applicable:

- Accession codes, unique identifiers, or web links for publicly available datasets
- A description of any restrictions on data availability
- For clinical datasets or third party data, please ensure that the statement adheres to our policy

The genetic data from MCPS – including the genotype array data, TOPMed-imputed data, exome data and whole genome sequence data – are available for sharing with bona fide academic researchers in Mexico through access to a DNAnexus research analysis platform powered by Amazon Web Services. Researchers in Mexico who are interested in obtaining these and/or the non-genetic data for specific academic research purposes, or in collaborating with MCPS investigators on a specific research proposal, should first visit the study's Oxford-hosted webpage (URLs) to download the Data and Sample Access Policy in English or Spanish. The non-genetic data available for sharing may be reviewed on the study's online Data Showcase (URLs). The Data and Sample Access Policy aims to promote equity in research by giving preferential access to researchers in Mexican institutions whereby such applicants have free access with a period of exclusivity over researchers in other parts of the world (though principal investigators in Mexico may still choose to collaborate with researchers in other parts of the world on their approved projects if they wish). Researchers in Mexican institutions are also provided with analysis 'credits' to cover the cost of running their analyses on the platform and downloading their results. For academic researchers in other parts of the world the genetic data will be made available for open access sharing only after the end of the exclusivity period for Mexican researchers (the duration of which is constantly reviewed but in no circumstances will exceed 2 years). Researchers in high-income countries will be required to pay a nominal data-access fee (to cover the administrative costs associated with processing data requests and maintaining the data analysis platform) but there will be no data access fee for researchers in low or middle-income countries. The reason for giving Mexican researchers preferential access to the data generated in Mexico is to foster equity and provide an opportunity to develop local research capacity. Otherwise, given the disproportionate analytic capacity in, for example, North America and Western Europe, as compared with Mexico, there is a risk that future analyses of these data will be dominated by researchers from outside Mexico. The MCPS ancestry specific allele frequencies are available in a public browser which includes options for direct download (see URLs). The MCPS10k imputation reference panel described in this manuscript will be made available for imputation through the University of Michigan Imputation server (see URLs). The GRCh38 reference accession code is https://www.ncbi.nlm.nih.gov/datasets/genome/GCF_000001405.26/.

Human research participants

Policy information about studies involving human research participants and Sex and Gender in Research.

Reporting on sex and gender	Sex of study participants was collected as part of the study, and is detailed in the paper Tapia-Conyer, R., et al (2006) Cohort Profile: The Mexico City Prospective Study, International Journal of Epidemiology, Volume 35, Issue 2, April 2006, Pages 243–249, https://doi.org/10.1093/ije/dyl042<br><br>Sex information was used in some of the analysis to infer family structure.<br><br>Two-thirds were women.
Population characteristics	The details of the population characteristics are described in the paper Tapia-Conyer, R., et al (2006) Cohort Profile: The Mexico City Prospective Study, International Journal of Epidemiology, Volume 35, Issue 2, April 2006, Pages 243–249, https://doi.org/10.1093/ije/dyl042<br><br>The mean age at survey was 53 (with 92% aged 35–74), and 60% lived in Iztapalapa.
Recruitment	The recruitment strategy is described in the paper Tapia-Conyer, R., et al (2006) Cohort Profile: The Mexico City Prospective Study, International Journal of Epidemiology, Volume 35, Issue 2, April 2006, Pages 243–249, https://doi.org/10.1093/ije/dyl042
Ethics oversight	Approval for the study was given by the Mexican Ministry of Health, the Mexican National Council of Science and Technology (0595 P-M) and the Central Oxford Research Ethics Committee (C99.260) and the Ethics and Research commissions from the Medicine Faculty at the National Autonomous University of Mexico (UNAM) (FMED/CI/SPLR/067/2015). All study participants provided written informed consent.

Note that full information on the approval of the study protocol must also be provided in the manuscript.

Field-specific reporting

Please select the one below that is the best fit for your research. If you are not sure, read the appropriate sections before making your selection.

☒ Life sciences ☐ Behavioural & social sciences ☐ Ecological, evolutionary & environmental sciences

For a reference copy of the document with all sections, see nature.com/documents/nr-reporting-summary-flat.pdf

Life sciences study design

All studies must disclose on these points even when the disclosure is negative.

Sample size	Sample size was not predetermined. Genotyping and sequencing was carried out on all the samples provided to the Regeneron Genetics Center from the UK Biocentre. The methods section details all QC performed on the genotype, exome sequencing and whole genome sequencing datasets.
Data exclusions	Of the 143,440 samples exome sequenced, 2,394 (1.7%) did not pass one or more of our QC metrics and were subsequently excluded. Criteria for exclusion were: disagreement between genetically-determined and reported sex (n=1,032); high rates of heterozygosity/contamination (VBID > 5%) (n=249); low sequence coverage (less than 80% of targeted bases achieving 20X coverage) (n=29); genetically-identified sample duplicates (n=1,062 total samples); WES variants discordant with genotyping chip (n=8); uncertain linkage back to a study participant (n=259); and instrument issue at DNA extraction (n=6). The remaining 141,046 samples were then used to compile a project-level VCF (PVCF) for downstream analysis, using the GLnexus joint genotyping tool. This final dataset contained 9,950,580 variants.<br><br>Of the 10,008 samples that were whole genome sequenced, 58 (0.6%) did not pass one or more of our QC metrics and were subsequently excluded. Reasons for exclusion were: disagreement between genetically-determined and reported sex (n=16); high rates of heterozygosity/contamination (VBID > 5%) (n=10); genetically-identified sample duplicates (n=19 total samples); and uncertain linkage back to a study participant (n=14). The remaining 9,950 samples were then used to compile a project-level VCF (PVCF) for downstream analysis, using the GLnexus joint genotyping tool. This final dataset contained 158,464,363 variants.
Replication	The paper described a population genetic study and describes the properties and uses of the data collected. As such there is no need to replicated the data collection.
Randomization	Individuals in the study were not being assigned to any experimental protocol or treatment and so randomization was not needed.
Blinding	Individuals in the study were not being assigned to any experimental protocol or treatment and so rblinding was not needed.

Reporting for specific materials, systems and methods

We require information from authors about some types of materials, experimental systems and methods used in many studies. Here, indicate whether each material, system or method listed is relevant to your study. If you are not sure if a list item applies to your research, read the appropriate section before selecting a response.

Materials & experimental systems

n/a	Involved in the study	
☒	☐	Antibodies
☒	☐	Eukaryotic cell lines
☒	☐	Palaeontology and archaeology
☒	☐	Animals and other organisms
☒	☐	Clinical data
☒	☐	Dual use research of concern

Methods

n/a	Involved in the study	
☒	☐	ChIP-seq
☒	☐	Flow cytometry
☒	☐	MRI-based neuroimaging
